# Generation and Functional Analysis of Defective Viral Genomes during SARS-CoV-2 Infection

**DOI:** 10.1128/mbio.00250-23

**Published:** 2023-04-19

**Authors:** Terry Zhou, Nora J. Gilliam, Sizhen Li, Simone Spandau, Raven M. Osborn, Sarah Connor, Christopher S. Anderson, Thomas J. Mariani, Juilee Thakar, Stephen Dewhurst, David H. Mathews, Liang Huang, Yan Sun

**Affiliations:** a Department of Immunology and Microbiology, University of Rochester Medical Center, Rochester, New York, USA; b Medical Scientist Training Program, University of Rochester School of Medicine and Dentistry, Rochester, New York, USA; c Translational Biomedical Sciences PhD Program, University of Rochester School of Medicine and Dentistry, Rochester, New York, USA; d School of Electrical Engineering & Computer Science, Oregon State University, Corvallis, Oregon, USA; e Department of Pediatrics and Center for Children’s Health Research, University of Rochester, Rochester, New York, USA; f Department of Biostatistics and Computational Biology, University of Rochester School of Medicine and Dentistry, Rochester, New York, USA; g Department of Biomedical Genetics, University of Rochester School of Medicine and Dentistry, Rochester, New York, USA; h Department of Biochemistry & Biophysics and Center for RNA Biology, University of Rochester Medical Center, Rochester, New York, USA; University of Maryland School of Medicine; Johns Hopkins Bloomberg School of Public Health

**Keywords:** defective viral genomes, SARS-CoV-2, recombination, secondary structure, type I/III IFN responses, human epithelial cells, RNA secondary structure

## Abstract

Defective viral genomes (DVGs) have been identified in many RNA viruses as a major factor influencing antiviral immune response and viral pathogenesis. However, the generation and function of DVGs in SARS-CoV-2 infection are less known. In this study, we elucidated DVG generation in SARS-CoV-2 and its relationship with host antiviral immune response. We observed DVGs ubiquitously from transcriptome sequencing (RNA-seq) data sets of *in vitro* infections and autopsy lung tissues of COVID-19 patients. Four genomic hot spots were identified for DVG recombination, and RNA secondary structures were suggested to mediate DVG formation. Functionally, bulk and single-cell RNA-seq analysis indicated the interferon (IFN) stimulation of SARS-CoV-2 DVGs. We further applied our criteria to the next-generation sequencing (NGS) data set from a published cohort study and observed a significantly higher amount and frequency of DVG in symptomatic patients than those in asymptomatic patients. Finally, we observed exceptionally diverse DVG populations in one immunosuppressive patient up to 140 days after the first positive test of COVID-19, suggesting for the first time an association between DVGs and persistent viral infections in SARS-CoV-2. Together, our findings strongly suggest a critical role of DVGs in modulating host IFN responses and symptom development, calling for further inquiry into the mechanisms of DVG generation and into how DVGs modulate host responses and infection outcome during SARS-CoV-2 infection.

## INTRODUCTION

Respiratory tract infection of severe acute respiratory syndrome coronavirus 2 (SARS-CoV-2) results in various immunopathologies underlying coronavirus disease 2019 (COVID-19). Its symptoms vary from asymptomatic infection to milder/moderate disease and further critical illness, including respiratory failure and death. Immune responses in COVID-19 patients of various disease severities have been studied (1–3). In general, the broad induction of interferon (IFN) responses and antiviral genes is associated with milder/moderate COVID-19, whereas severe COVID-19 is often characterized by blunt early IFN responses and elevated proinflammatory cytokine expression in nasopharyngeal mucosa (4–8). Investigation of how IFN responses are induced by SARS-CoV-2 infection, especially in early IFN stimulation in some patients, requires further study.

During SARS-CoV-2 infection, in addition to full-length viral genomes and single-nucleotide mutations, three major types of viral RNAs are generated from nonhomologous recombination that are critical for viral pathogenesis, including subgenomic mRNAs (sgmRNAs), structural variants (SVs), and defective viral genomes (DVGs). The viral replication-transcription complex performs recombination at specific transcription regulatory sequences (TRSs) to generate a set of sgmRNAs, which subsequently translate into viral structural proteins (9–12). SVs comprise small insertion/deletions that allow the variant genome to independently replicate and transmit. Numerous SVs have been described, including small deletions in viral spike protein that alter the fitness and virulence of SARS-CoV-2 isolates (13–16). Different from sgmRNAs and SVs, SARS-CoV-2 DVGs contain large internal deletions and have recombination positions distinct from TRSs while retaining 5′ and 3′ genomic untranslated regions (UTRs) (17).

There are two major types of DVGs, namely, deletion and copy-back, of which both are also known as defective viral or interfering RNAs (D-RNAs), and their replication relies on viral machinery provided by coinfected homologous full-length viruses (18–20). When accumulated to a high level, DVGs can interfere with full-length viral genome production by stealing essential viral elements from full-length viruses (21, 22). The deletion type of DVGs is generated widely during replication of most positive-sense RNA viruses (23, 24) and influenza (25). The interference activity has been reported for influenza viruses (26) and multiple non-SARS-CoV-2 coronaviruses (CoVs), such as SARS-CoV (27), mouse hepatitis virus (MHV) (28), bovine CoV (29), avian infectious bronchitis virus (IBV) (30), transmissible gastroenteritis virus (31), and Middle East respiratory syndrome-CoV (MERS-CoV) (17). In addition to interference activity, DVGs from influenza A virus have strong IFN stimulation (32) and are reported to promote viral persistence *in vitro* (26, 33, 34). More importantly, DVGs are largely observed in nasal samples from patients positive for influenza, and their abundance is negatively correlated with patient disease severity, indicating the critical roles of DVGs in host responses and clinical outcome (35). The current approach to identify DVGs from SARS-CoV-2 infection is through short-read and long-read next-generation sequencing (NGS). Several algorithms, such as DI-tector (36), iral Opensource DVG Key Algorithm (VODKA) (37), and virus recombination mapper (ViReMa) (38), and metasearch tool DVGfinder (39) have been developed to specifically detect the reads containing the recombination sites of DVGs. Using these approaches, DVGs are documented in SARS-CoV-2-infected Vero E6 cells (40, 41) and in nasal samples of COVID-19 patients (42). Long-read NGS, such as full-length iso-seq and Nanopore direct transcriptome sequencing (RNA-seq), further confirmed that substantial TRS-independent deletions identified from short-read NGS are from SARS-CoV-2 genomes and maintain two genomic ends (17, 43). Additionally, identical deletions are found in various transcripts encoding distinct sgmRNAs (43), strongly suggesting that even deletions in sgmRNAs are likely to have originated from viral genomes since deletions existing in the viral genome can be used as the template to generate a set of sgmRNAs with the same deletions during transcription.

Despite DVGs playing such an important role in viral pathogenesis, their function in SARS-CoV-2 biology is less known. Recent reports show that synthetic SARS-CoV-2 DVGs, named therapeutic interfering particles (TIPs), exhibit substantial reduction on viral load across different viral variants when delivered in hamsters (40) and mice (42) before or shortly after infection, demonstrating the potential use of SARS-CoV-2 DVGs as a new class of antiviral intervention by interfering with genomic replication. No reports have identified the role of DVGs, especially naturally generating ones, in IFN responses and viral persistence for SARS-CoV-2 infection so far. Interestingly, a COVID-19 cohort study (43) indicated that the abundance of TRS-independent deletions (>20 nucleotides [nt]) is significantly higher in symptomatic patients than that in asymptomatic patients, suggesting a potential role of DVGs in modulating host responses and symptom development in COVID-19 patients.

As our interest lies with the generation of natural DVGs, in relation to viral pathogenesis, we used a pipeline based on ViReMa combined with sequence filtering via RStudio to specifically identify TRS-independent DVGs with deletion lengths larger than 100 nt. We required deletions longer than 100 nt to distinguish them from the small deletions often observed in Vero E6 infection/passaging or in variance of concerns. We examined both the number of total DVG reads, named total DVGs, and the number of unique DVG junctions, named unique DVGs, to estimate the total DVG amount and the initial DVG production, respectively, during infection. We identified total/unique DVGs with various amounts and degrees of junction frequency from multiple NGS data sets that either were publicly available or were from our own infections. Interestingly, we found DVG junctions consistently clustered in several genomic hot spots among different NGS data sets and that secondary structures within the viral genome are likely to guide the recombination. Functionally, we found that with similar infection levels, samples with more total amounts of DVGs had enhanced type I/III IFN responses than samples with less or no DVGs, indicating the potential IFN stimulation of SARS-CoV-2 DVGs. In support, the analysis of single-cell RNA-seq of infected primary human lung epithelial (PHLE) cells showed an earlier primary IFN expression (IFNB1 and IFNL1) in DVG^+^ cells than in DVG^−^ cells. Finally, we applied our DVG analysis to several published NGS data sets from nasal samples of COVID-19 patients. We found persistent DVG reads in one immunosuppressive patient and higher DVG abundance in symptomatic patients than that in asymptomatic patients. Taken together, our analyses demonstrate critical roles of DVGs in modulating host IFN responses, viral persistence, and clinic outcome for SARS-CoV-2 infection.

## RESULTS

### DVGs are produced ubiquitously during SARS-CoV-2 infection both *in vitro* and in patients.

**FIG 1:**
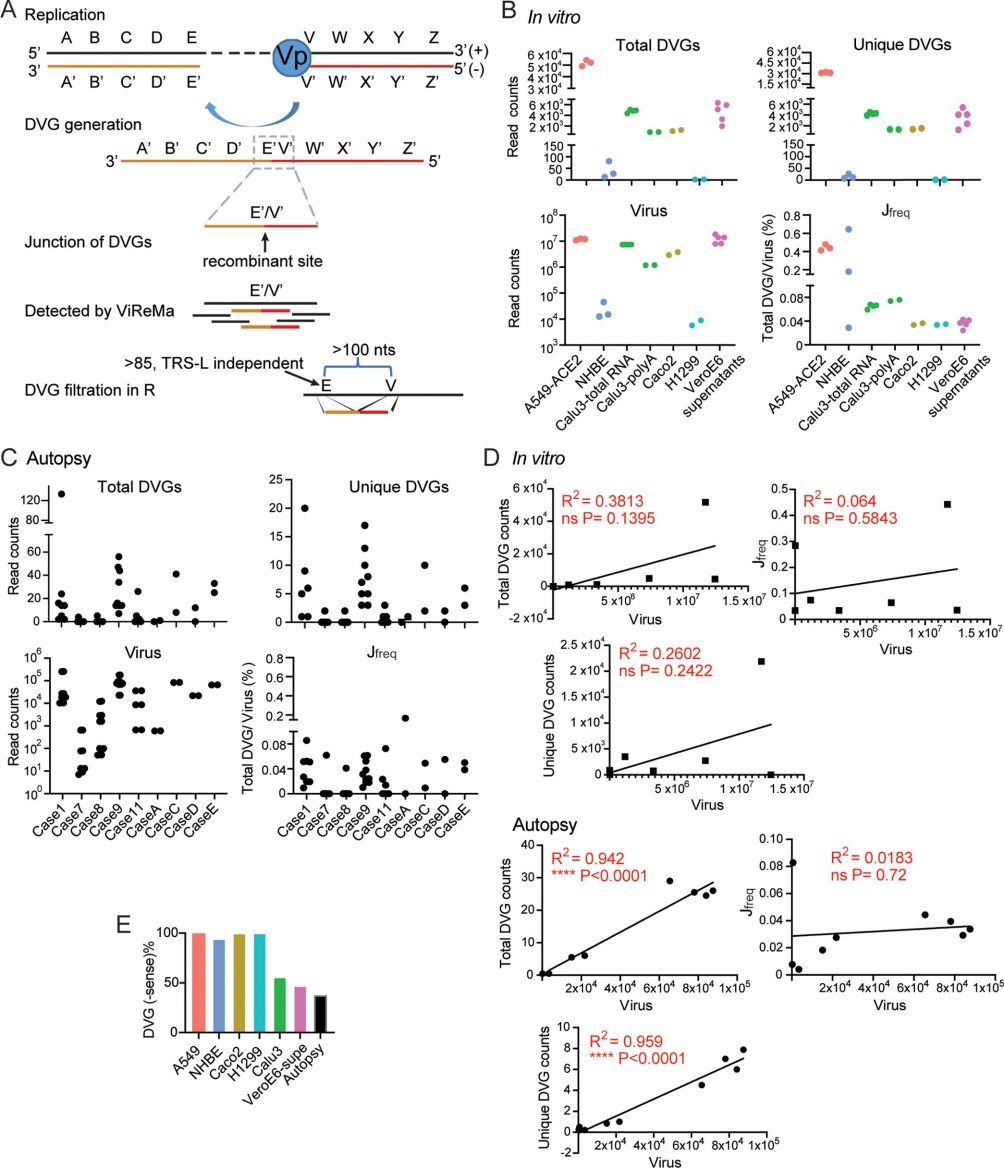
DVGs were generated ubiquitously in SARS-CoV-2 *in vitro* infections and autopsy tissues of COVID-19 patients. (A) Schematic representation of DVG generation from a positive-sense viral genome and the general principle of ViReMa identification of deletion DVGs. The V’ site represents the breakpoint and the E’ site represents the rejoin point of the viral polymerase in the formation of DVGs. The gray dashed box marks the recombinant site that distinguishes DVGs from full-length viral genomes, which are identified by ViReMa and further filtered using two criteria shown in the graph. (B) The respective total and unique DVG read counts, viral read counts, and J_freq_ percentages were graphed for each of the *in vitro* samples, including the infected cells and supernatants. (C) The respective total and unique DVG read counts, viral read counts, and J_freq_ percentages were graphed for autopsy lung tissues of 9 DVG^+^ COVID-19 patients. Each case represents one patient, and different dots represent RNA-seq from different locations of the same lung tissues. (D) The correlations between total DVG counts, unique DVG counts, J_freq_, and viral read counts were plotted for *in vitro* and autopsy samples. ****, *P* < 0.0001 by Pearson’s correlation. (E) The percentages of −sense DVGs among total DVGs in *in vitro* and autopsy samples were shown.

To examine whether DVGs can be detected universally during SARS-CoV-2 infections, we used the virus recombination mapper (ViReMa) pipeline combined with R filtering (see Text S1 in the supplemental material) to specifically map the DVG recombinant sites (Fig. 1A) in multiple next-generation sequencing (NGS) data sets. As reported previously, ViReMa can agnostically detect RNA recombination events and report these junction positions in BED files. Reported junction positions include sgmRNAs, of which their junctions contain leader transcriptional-regulatory signal (TRS-L; within the first 85 nt of leader) and other recombinant RNAs with their jumping positions that are far away from TRS-L. We defined our targeted DVGs as TRS-L-independent RNA species bearing deletions larger than 100 nt (Fig. 1A). Using these criteria, we first examined DVGs in 4 publicly available *in vitro*-infected NGS data sets with various cell types, multiplicities of infection (MOIs), viral stocks, and sample origins (see Table S1 in the supplemental material). We found that DVGs can be detected in all examined data sets ranging from several counts to several thousand counts for both total and unique amount (Fig. 1B). As the MOIs and sequencing depth varied significantly among different data sets, we normalized DVG levels by junction frequency (J_freq_), a ratio of total DVG counts over virus counts, where virus counts were the total amount of reads fully aligned to the reference viral genome. We observed two groups of J_freq_, namely, <0.1% and 0.1% to 1%. A549-ACE2-infected cells have the highest J_freq_, whereas infections in PHLE cells varied. In addition, either total RNA or polyA-enriched RNA were used for NGS for Calu3-total RNA and Calu3-polyA, respectively. Both samples had very similar J_freq_ values, suggesting J_freq_ is robust to different library preparation methods. Interestingly, we detected DVGs, although with low J_freq_, in the supernatants collected from infected Vero E6 cells, suggesting that certain DVG species generated within infected cells, potentially the DVGs containing packaging signals, were able to be packaged into virions and released into supernatants. As no RNase treatment was indicated in this NGS, we cannot exclude that nonpackaging DVGs leaked from dying cells to supernatants.

10.1128/mbio.00250-23.7 TABLE S1 Summary of all samples from published data sets. Download Table S1, DOCX file, 0.02 MB. Copyright © 2023 Zhou et al. 2023 Zhou et al. https://creativecommons.org/licenses/by/4.0/ This content is distributed under the terms of the Creative Commons Attribution 4.0 International license.

10.1128/mbio.00250-23.10 TEXT S1 Pipeline scripts used for the identification of DVGs by ViReMa, postfiltration in R, and analyses of host responses associated with DVGs. Download Text S1, PDF file, 1.2 MB. Copyright © 2023 Zhou et al. 2023 Zhou et al. https://creativecommons.org/licenses/by/4.0/ This content is distributed under the terms of the Creative Commons Attribution 4.0 International license.

We then examined DVGs in autopsy tissues from patients who unfortunately died from COVID-19 complications (GSE150316). We analyzed lung, heart, jejunum, liver, and kidney samples collected from 19 cases, and DVGs were observed in only lung tissues in 9 cases (Fig. 1C). Their total and unique DVG counts were close to the levels observed in infections in PHLE cells but were much less than infections in cell lines, such as A549-ACE2, Vero E6, Calu3, and Caco2. J_freq_ values from autopsy tissues were mostly less than 0.1%, which was comparable with the lower range of J_freq_s observed from *in vitro* infections. Next, we sought to examine the relationship among total DVGs, unique DVGs (DVG production), and viral counts. The numbers of total DVGs and unique DVGs were positively correlated with each other for both autopsy tissues and *in vitro* infections (see Fig. S1A and B in the supplemental material). Interestingly, we observed strong positive correlations between total DVGs versus virus counts and unique DVGs versus viral counts for autopsy tissues but not for *in vitro* infections (Fig. 1D). In addition, J_freq_ was not significantly correlated with virus replication level. It is noted that both negative-sense (−sense) and positive-sense (+sense) DVGs were detected in all NGS libraries (Fig. 1E). As all NGS libraries used in this study were not derived from strand-directed sequencing, we could not obtain DVG orientation in the original RNA samples, which is especially the case for Vero E6 supernatants, where ~40% of the identified DVGs in this library were −sense. Together with the previous reports in nasal specimens of COVID-19 patients (42) and our own analysis, we concluded that DVGs are generated ubiquitously during SARS-CoV-2 infection *in vitro* and in patients.

10.1128/mbio.00250-23.1 FIG S1 Association between total DVG read counts with unique DVG read counts. The correlations between total and unique DVG read counts were plotted for *in vitro* samples (A); autopsy samples (B); PHLE cells (C); 1 dpi, 2 dpi, and 3 dpi single-cell samples (D); asymptomatic and symptomatic samples (E); and longitudinal time point samples from an immunosuppressive patient (F). Download FIG S1, TIF file, 1.8 MB. Copyright © 2023 Zhou et al. 2023 Zhou et al. https://creativecommons.org/licenses/by/4.0/ This content is distributed under the terms of the Creative Commons Attribution 4.0 International license.

### Recombination sites of SARS-CoV-2 DVGs were clustered in certain genomic hot spots.

**FIG 2:**
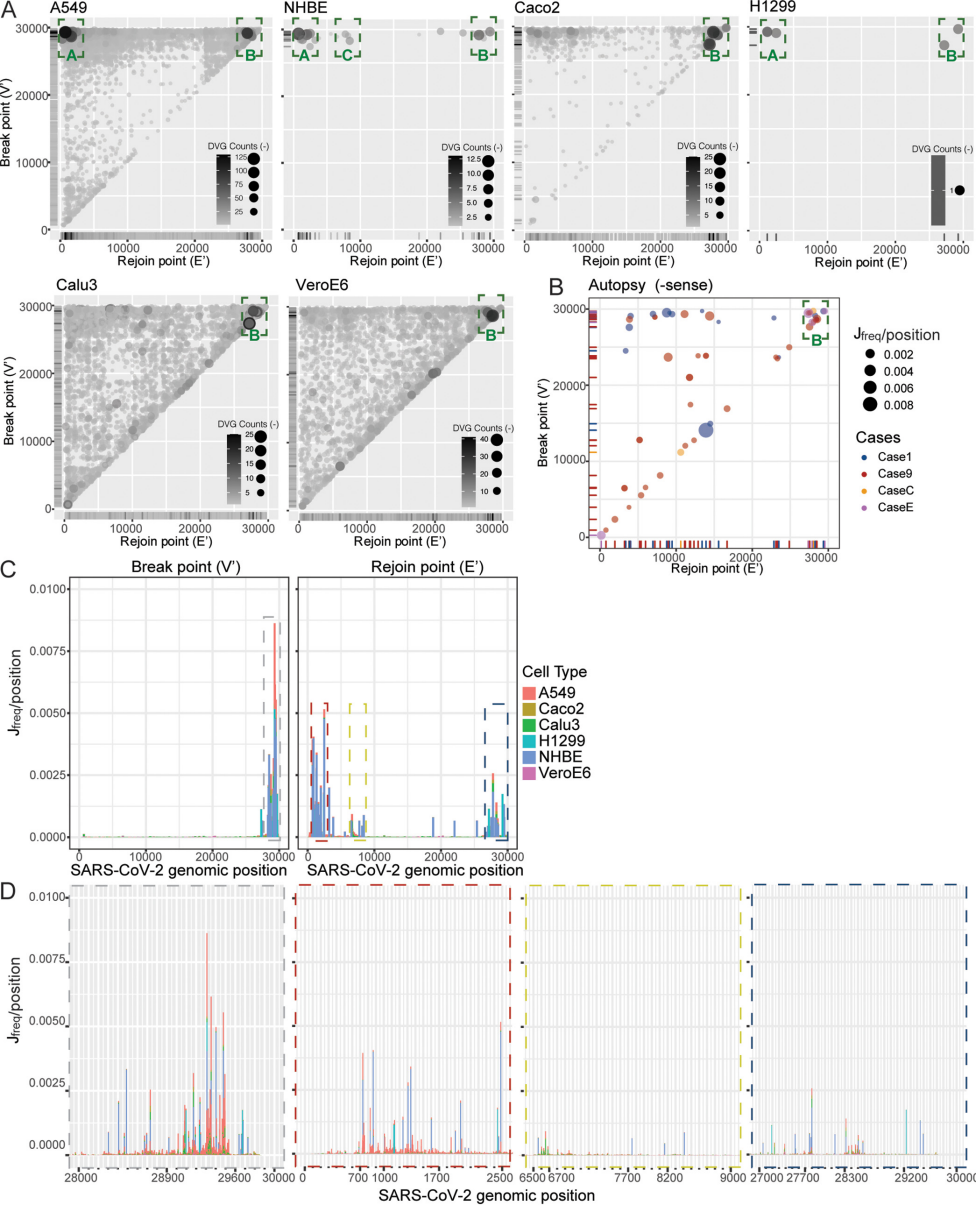
Four genomic hot spots were identified for DVG formation. Break point (V’) and rejoin point (E’) distributions for −sense DVGs from *in vitro* samples (A) and autopsy samples (B). Circle size and color intensity indicated the DVG counts for *in vitro* infection and J_freq_ for autopsy samples. The green dashed boxes represented hot spots clustered with DVG junctions. (C) Breakpoint (V’) and rejoin point (E’) distributions by J_freq_ per position for all *in vitro* samples. The dashed boxes indicated hot spots with high concentrations of break or rejoin points. The width of each bar represented 300 nt. (D) Detailed positions of 4 identified hot spots are clustered with DVG breakpoints and rejoin points. The color of the dashed outline around each graph indicated the corresponding hot spot with the same color in C. The width of each bar represented 10 nt.

To characterize positions of the recombination sites of DVGs, we graphed the actual junction positions of all identified DVGs from *in vitro* infections from different cells and DVG^+^ autopsy tissues. As both +sense and −sense DVGs were identified, we examined their distributions separately and first analyzed the junction positions of the −sense DVGs. Interestingly, we found that their generation was clustered in three conserved genomic hot spots, indicated as junction areas A, B, and C (green boxes in Fig. 2A and B). Among them, area B was observed in all infections and area A was observed largely in infected cells but was absent in the supernatants from infected Vero E6 cells. As DVGs that formed in junction area A contained the largest deletion compared with B and C, it is possible that DVGs within area A lack the package signal and thus were less efficiently released into supernatants. To further identify the genomic hot spots for DVG break and rejoin points, we graphed their locations separately based on the junction frequency per DVG position. We identified one major hot spot for breakpoint, corresponding to genomic positions 28200 to 29750 (highlighted in gray dashed box in Fig. 2C, details in Fig. 2D). Additionally, three major rejoin hot spots were identified, including 700 to 2500 (red box), 6500 to 8200 (yellow box), and 27000 to 29400 (blue box). When comparing the distribution between −sense and +sense DVGs, we observed that rejoin points (V) of +sense DVGs shared the same hot spots with breakpoint (V’) of −sense DVGs (see Fig. S2A and B in the supplemental material versus Fig. 2A and B). This finding suggests that the junction positions of −sense and +sense DVGs are correlated, likely resulting from their self-replication. Finally, we examined whether common DVGs can be detected from different infections or different autopsy tissues. We only identified common DVGs from different *in vitro* infections within the same RNA-seq data set (likely used the same viral stock for infections, representative ones are in Table S2 in the supplemental material). We did not find any common DVGs from different autopsy tissues. Taken together, our analysis from multiple NGS data sets indicated that SARS-CoV-2 DVGs are not generated randomly, rather they are formed at specific genomic regions.

10.1128/mbio.00250-23.2 FIG S2 Additional analysis for junction distribution of DVGs identified in bulk RNA-seq and scRNA-seq. (A and B) The distribution of +sense DVG junctions in SARS-CoV-2-infected Calu3 cells, Vero E6 supernatants, and autopsy samples used in Fig. 1, as more than half of DVGs identified in those samples were +sense. Circle size and color intensity in A indicated DVG read counts (+sense) from Calu3 and Vero E6, whereas the size and color of circles in B indicated the J_freq_ at that position and lung cases in autopsy samples, respectively. The green dashed boxes represented genomic hotspots for DVG junctions. (C to F) Graphed DVGs of NGS used in Fig. 4. Junction distributions for identified −sense (C) and +sense (D) DVGs from infected PHLE cells of different age groups were graphed as scatterplots. Circle color represented harvest time postinfection or patient age group. (E) The location distribution of the breakpoint and rejoin point of −sense DVGs were plotted separately as a bar graph. The dashed boxes indicated hotspots with high concentrations of breakpoints or rejoin points. The width of each bar represented 300 nucleotides. (F) Detailed positions of identified hot spots clustered with −sense DVG breakpoints and rejoin points. The color of the dashed outline around each graph indicated the corresponding hotspot with the same color in E. The width of each bar represented 10 nucleotides. (G) Represented DVG junction distribution from scRNA-seq used in Fig. 5 and 6. Breakpoint (E) and rejoin point (V) distributions of +sense DVGs were graphed at different time points postinfection. Circle size represented cell count per position, and circle color represents the length of deletions in DVGs. Download FIG S2, TIF file, 7.5 MB. Copyright © 2023 Zhou et al. 2023 Zhou et al. https://creativecommons.org/licenses/by/4.0/ This content is distributed under the terms of the Creative Commons Attribution 4.0 International license.

10.1128/mbio.00250-23.8 TABLE S2 Common DVGs identified from *in vitro* infections. Download Table S2, DOCX file, 0.03 MB. Copyright © 2023 Zhou et al. 2023 Zhou et al. https://creativecommons.org/licenses/by/4.0/ This content is distributed under the terms of the Creative Commons Attribution 4.0 International license.

### Validation of DVG junctions identified by ViReMa.

**FIG 3:**
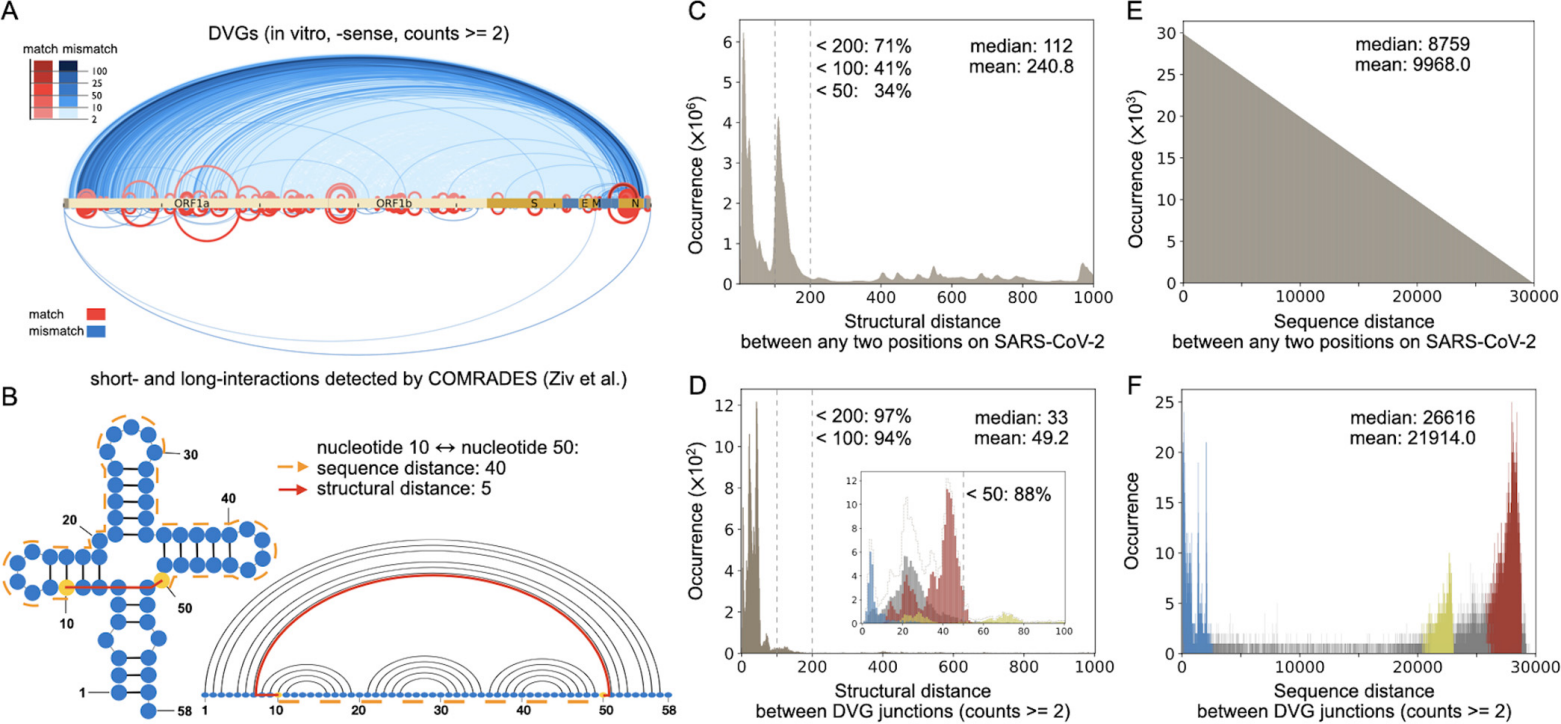
The correlation between DVGs and secondary structures. (A) Comparison between DVG junction positions (top, *in vitro*, −sense DVGs) and chimeric reads from COMRADES (bottom) along the full-length SARS-CoV-2 genome (45). The red arches represented DVG positions that match COMRADES cross-links, and the blue arches represented positions that do not match cross-links. (B) Example that compared sequence distance and structural distance. The structural distance between nucleotides 10 to 50 is only 5 (red solid path that includes a connection across a base pair), while the sequence distance is 40 (orange dashed path). (C and D) The distribution of all structural distances between any two positions in SARS-CoV-2 (C) and between SARS-CoV-2 DVG junction positions (D). The percentages of distances less than 50, 100, and 200 were indicated. (E and F) As a negative control, the distribution of all sequence distances between any two positions in SARS-CoV-2 (E) and between SARS-CoV-2 DVG junction positions (F). The mean and median distances of all distributions were annotated in C to F. In D and F, the blue, yellow, and red bars corresponded to three hot spots annotated in Fig. 2C, while the gray bars were out of the range of these detected hot spots. The inset in D distinguished the structural distance distributions of three hot spots and the rest up to a structural distance of 100. The dashed contour in the inset represented the sum of all distributions for the same structural distance, and it was with the same shape as the major figure in D. In both C and E, the total occurrence of all distances equals the number of any two positions along SARS-CoV-2, and in D and F, the total occurrence of all distances is the same as the number of DVG data points (with counts 2 or above).

**FIG 4:**
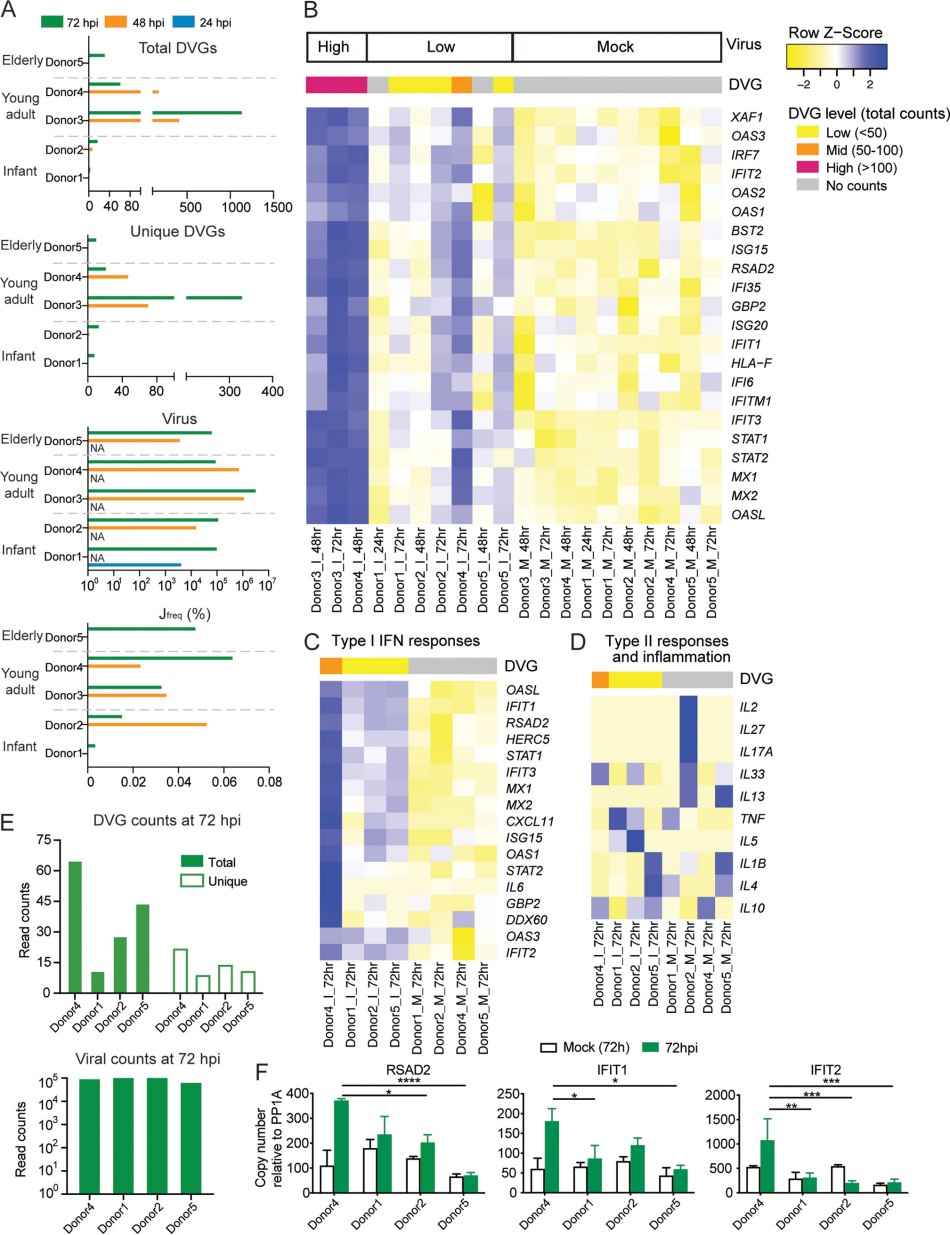
DVGs influence type I/III interferon responses in infected PHLE cells. PHLE cells of donors from different age groups were infected with SARS-CoV-2 at an MOI of 5. Samples were harvested at designated time points postinfection. (A) Viral read counts, total and unique DVG read counts, and J_freq_ were graphed for all samples and grouped by donor age group and time points. NA indicated that the samples were not available for RNA-seq and thus no data were collected. (B) Differential expression levels of genes related to type I interferon responses were graphed as a heatmap for all infected samples. Samples were grouped by viral infection level. The following DVG levels of each sample were indicated by different color codes on top of the heatmap: low DVG level (<50 total read counts), mid (50 to 100), and high (>100). (C and D) Four infected samples at 72 hpi with a similar level of viral counts were selected to compare their IFN responses (C) and other gene expression unrelated to type I/III IFN responses (D). (E) Viral read counts and total and unique DVG read counts for the selected 4 infected samples at 72 hpi were graphed. (F) The expression level of RSAD2, IFIT1, and IFIT2 from those 4 selected samples at 72 hpi were further confirmed by qPCR. ****, *P* < 0.0001; ***, *P* < 0.001; **, *P* < 0.01; *, *P* < 0.05; by two-way analysis of variance (ANOVA) followed by Turkey’s multiple-comparison test.

For our analyses, we did not identify any DVG junction reads in any mock samples (all mock samples are not infected with any viruses), suggesting the noise or potential artifact of our pipeline causing false-positive DVGs is close to zero. To further validate the DVG junction reads in our study, we randomly selected 15 unique DVG species (out of a total of over 200 DVG reads) from analyzed NGS data sets used in Fig. 1 to 4 (both top hits and DVGs only with 1 count, visualized as sushi plot in IGV shown on the left in Fig. S3A in the supplemental material) and extracted their read sequences from fastaq files. Extracted read sequences were then searched using BLAST+ for verification of their orientation and junction positions. All blasted reads were junction reads with correct orientation and with their junction positions either exactly matching or several nucleotides away from the ViReMa-identified junction sites (Fig. S3A, right). When further examining those ones with different junction positions by manual alignment, we found that the mismatches of positions are due to the ambiguity region in the junction region as reported before (38) (highlighted in yellow in Fig. S3A). Sometimes we observed that the nucleotides immediately upstream of the breakpoint were identical to the nucleotides immediately downstream of the rejoin point, which lead to the ambiguity of the exact junction sites. We think these errors can be tolerated, as they still accurately identify DVG junction reads with inaccurate recombination sites off by only a small number of nucleotides. Based on this finding, we think that the majority of ViReMa-identified postfiltering reads are true junction reads, even ones with only 1 count, and thus, we decided to include them for the following analyses. Finally, to experimentally verify the DVG hot spot identified by ViReMa, we infected Vero E6 cells with the SARS-CoV-2 HongKong strain at an MOI of 1. Infected cells were harvested at 72 hours postinfection (hpi), their RNAs were extracted, and reverse transcriptase PCR (RT-PCR) was conducted, specifically targeting DVGs formed at hot spot A. Although our infection is different from that for NGS data sets used in Fig. 1 and 2, we think that the hot spots identified from all these publicly available data sets should be conserved, and thus, DVGs formed at those hotpots are expected to be detected in our infection. We used one primer at the 3′ UTR (SC-R1) and the other primer within nsp1 (539F) to detect potential deletions. Only deleted genomes can be detected in this case, as the full-length viral genome will be too long for amplification. We observed two prominent bands in the infected cells but not in the mock cells (Fig. S3B). We cut those two bands for further cloning followed by Sanger sequencing. Sequencing results indicated that both bands contained deletions with their specific junction at 724 to 29406 and 828 to 29058, of which both were within the hot spot A identified by ViReMa (Fig. S3C).

10.1128/mbio.00250-23.3 FIG S3 Validation of DVG junctions identified by ViReMa. (A) We used BLAST+ followed by manual alignment to the reference genome (MT020881) to confirm DVG reads identified by ViReMa. For each sample, we used igvtools (Integrative Genomics Viewer, IGV 2.15.4) to visualize DVG reads from bam/sam files (raw aligned files from ViReMa). Then we randomly selected unique DVG species in each sample and extracted their original read sequences from corresponding fastq or bam files, which were then subjected to a search via BLAST+ (NCBI) for junctions and were manually aligned against our reference genome to further confirm their sense and junction positions. Both junction tracks as sushi plots (left) and manual alignments of their corresponding read sequences to the reference genome (right) were shown. Red arches in sushi plots represent the read sequence with positive rightward (5′ to 3′) orientation relative to the reference. Blue arches in sushi plots represent the read sequence with negative leftward (complementary-reverse) orientation relative to the reference. The color of the arrows matched the color of corresponding DVG read sequence. Mismatches in the alignments were shaded gray, and the ambiguous regions of DVG junctions were marked by yellow. (B and C) Vero E6 cells were infected with SARS-CoV-2 at an MOI of 1 and harvested at 72 hpi followed by RNA extraction and RT-PCR specifically targeting DVGs formed at hot spot A. (B) The representative gel images of DVG-specific RT-PCR. Two prominent bands in B were cloned and further confirmed as deletions by Sanger sequencing with their actual sequences showing in C. Download FIG S3, TIF file, 3.7 MB. Copyright © 2023 Zhou et al. 2023 Zhou et al. https://creativecommons.org/licenses/by/4.0/ This content is distributed under the terms of the Creative Commons Attribution 4.0 International license.

### The RNA structural distance between SARS-CoV-2 DVG junction positions is shorter than any two random SARS-CoV-2 genomic positions.

Ziv et al. (44) developed COMRADES, which can probe RNA base pairing inside cells, and applied it to detect short- and long-range interactions along the full-length SARS-CoV-2 genome (45). Interestingly, the positions of SARS-CoV-2 DVG junctions correlated well with the pairings found by COMRADES (red arches in Fig. 3A), which suggests a role of RNA secondary structures in the formation of DVGs. The paired bases bring distant nucleotides in the primary sequence close and make it possible for the breaking and rejoining actions to occur around those close pairs. To further study the relationship between DVG junctions and the identified secondary structure within the SARS-CoV-2 genome, we calculated the structural distance between DVG junction positions, which is the shortest distance between two nucleotides by traversing the backbone and base pairs (red solid path in Fig. 3B) (46). We further extended this definition to allow competing base pairs from alternative secondary structures since many RNAs are known to populate multiple conformations in equilibrium and data in Ziv et al. (44) included alternative conformations of SARS-CoV-2.

We first analyzed the distribution of all structural distances between any two nucleotides in SARS-CoV-2, where 41% of the distances were under 100 (Fig. 3C) with a long tail up to 1,200. The median distance of the distribution was 112. However, for the structural distances between only SARS-CoV-2 DVG junction positions (counts, ≥2), the peak of the distribution shifted to the left with a smaller median value of 33, and the vast majority (94%) of distances were less than 100 (Fig. 3D). Therefore, the structural distances between DVG junction positions were substantially shorter than the distances between any two random positions, which indicated a strong correlation between secondary structures and DVG formation. Moreover, we observed that the larger the cutoff value for DVG counts, the greater the proportion of distances under 100 and the smaller the mean distance (see Fig. S4A in the supplemental material). For a negative control, we also evaluated the sequence distance, which is the distance between nucleotides based only on their positions along the primary sequence; in fact, it is a special case of structural distance without any secondary structure. We analyzed the sequence distance between any two nucleotides in SARS-CoV-2 and that between SARS-CoV-2 DVG junction positions (Fig. 3E and F). The distribution of sequence distances between any two nucleotides on SARS-CoV-2 was a triangular distribution. Most of the distances between DVG junctions were clustered similarly as the hot spots observed previously (Fig. 2C versus Fig. 3F), which is completely different from the distribution of structural distances of DVG junctions that had its peak on the left (Fig. 3C and D). For a positive control, we calculated the structural distance for all known sgmRNAs of the virus using the same approach. As expected, 89% viral sgmRNAs had the structural distance under 100 with a median value of 42 (Fig. S4B to F), which is also substantially shorter than the structural distances between any two random positions in the viral genome.

10.1128/mbio.00250-23.4 FIG S4 Additional analysis of structural distance for DVG junctions and sgmRNA junctions. (A) Additional analysis of structural distance (left) and sequence distance (right) for DVG junctions with more abundance. The first row showed the distributions over all pairs of positions, and the next rows represented distributions over DVG junctions with different cutoff values for counts (2, 5, 10). As the cutoff value increased, a greater proportion of distances are under 100, and the mean values get smaller. (B to F) Structure distance analysis of junction positions of SARS-CoV-2 TRS-L-dependent sgmRNAs. (B) Comparison between TRS-L-dependent sgmRNA junction positions (top, *in vitro*) and chimeric reads from COMRADES (bottom) along the full-length SARS-CoV-2 genome. The red arches represented sgmRNA junction positions that match COMRADES cross-links, and the blue arches represented positions that do not match crosslinks. The distributions of all structural distances between any two positions in SARS-CoV-2 genome (C) and between SARS-CoV-2 TRS-L dependent sgmRNA junction positions (D) were graphed. The percentage of distances less than 50, 100, and 200 were indicated. As a negative control, the distributions of all sequence distances between any two positions in SARS-CoV-2 (E) and between SARS-CoV-2 TRS-L-dependent sgmRNA junction positions (F) were graphed. Download FIG S4, TIF file, 5.2 MB. Copyright © 2023 Zhou et al. 2023 Zhou et al. https://creativecommons.org/licenses/by/4.0/ This content is distributed under the terms of the Creative Commons Attribution 4.0 International license.

### SARS-CoV-2 DVGs specifically enhanced type I/III IFN responses.

To understand the dynamics of SARS-CoV-2 DVGs during infection and how that affects host responses and viral replication, we infected primary human lung epithelial (PHLE) cells from donors of different age groups with the SARS-CoV-2 HongKong strain at an MOI of 5. Mock and infected cells were harvested at different time points postinfection (hpi) followed by bulk RNA-seq-ViReMa analysis. We observed DVGs as early as 48 hpi in cells from infants and younger adults, whereas in the elderly sample, we did not detect DVGs until 72 hpi (Fig. 4A), suggesting that DVG generation may be delayed in the elderly, who are more likely to display severe symptoms when infected. We observed the same genomic hot spots for DVG junction regardless of their age groups and time points (Fig. S2C to G). Strikingly, those hot spots were consistent with the ones identified from different cell lines (Fig. 2), autopsy lung tissues (Fig. 2), and the following single-cell RNA-seq analysis (Fig. S2G). Again, we observed that V (rejoin point of +sense DVGs) and V’ (breakpoint of −sense DVGs) shared the same hot spots and E (breakpoint of +sense DVGs) and E’ (rejoin point of −sense DVGs) shared the same hot spots (Fig. S2C versus Fig. S2D), indicating that our identified recombination sites were likely from DVGs capable of replication. No DVGs were identified from any mock samples.

As total and unique DVGs were again strongly positively correlated (Fig. S1C), samples with more total DVGs always had more unique DVGs. In order to examine the role of DVGs in host responses, we grouped our infected samples based on their total DVG counts and viral counts (criteria for DVG grouping shown in Fig. 4B). Three samples (Donor3_I_48hr, Donor3_I_72hr, and Donor4_I_48hr) with significantly high viral counts and high total DVG counts were categorized as “high group” (marked as pink in Fig. 4B and Fig. S5A in the supplemental material). When we compared this group with the rest infected samples, one cluster of genes (pink cluster shown on the left in Fig. S5A) were identified as upregulated in the high group. Gene Ontology (GO) enrichment analysis of this cluster was highly enriched in genes involved in type I IFN antiviral responses (Fig. S5B). A heatmap focusing on type I/III IFN-related genes confirmed that samples in the high group had enhanced gene expression compared with the rest of samples and that all mock samples had a basal level of expression (Fig. 4B). In order to test if the IFN stimulation is specific to DVGs, we selected 4 samples at 72 hpi with similar levels of viral counts but different levels of total DVGs and unique DVGs (Fig. 4E) to compare their type I/III IFN responses. We observed that the sample with more DVGs exhibited an enhanced expression of antiviral genes, such as IFIT1, IFIT3, and OASL, compared with samples with fewer DVGs (Fig. 4C), but this enhancement was not observed for genes in other pathways, such as type II responses and inflammation (Fig. 4D). This observation was further confirmed by quantitative PCR (qPCR) targeting selected interferon-stimulated genes (ISGs) (Fig. 4F). Together, these data, for the first time, suggest that SARS-CoV-2 DVGs enhance IFN production, as observed previously in other RNA viruses (32).

10.1128/mbio.00250-23.5 FIG S5 IFN stimulatory ability of DVGs during SARS-CoV-2 infection. (A) Differentially expressed genes between high-virus and low-virus groups were graphed as a heatmap for all samples in Fig. 4. The pink cluster shows the genes upregulated in the high-virus group, and the orange cluster shows the genes downregulated in the high-virus group. The virus group and the DVG level of each sample were both indicated on top of the heatmap. (B) Gene Ontology analysis of genes that were upregulated in the high-virus group (pink cluster shown in A) were graphed in R (GOplot). Circle size represented the number of genes in each pathway. Gene ratio represented the ratio of the number of genes in that pathway to the number of genes in the entire cluster. (C to D) Gene Ontology analysis of genes that were downregulated (C) and upregulated (D) in DVG^+^ cells relative to DVG^−^ cells at 3 dpi in scRNA-seq. Circle size represented the number of genes in each pathway. Gene ratio represented the ratio of the number of genes in that pathway to the number of genes in the entire cluster. (E) The average expression levels of selected genes related to type I interferon responses were graphed as a heatmap for the mock sample, the DVG^−^ group, and the DVG^+^ group within moderately infected cells at 2 dpi and 3 dpi (including cells both with and without gene expression). Download FIG S5, TIF file, 3.9 MB. Copyright © 2023 Zhou et al. 2023 Zhou et al. https://creativecommons.org/licenses/by/4.0/ This content is distributed under the terms of the Creative Commons Attribution 4.0 International license.

### Primary IFNs were expressed earlier in DVG^+^ cells with moderate infection.

**FIG 5:**
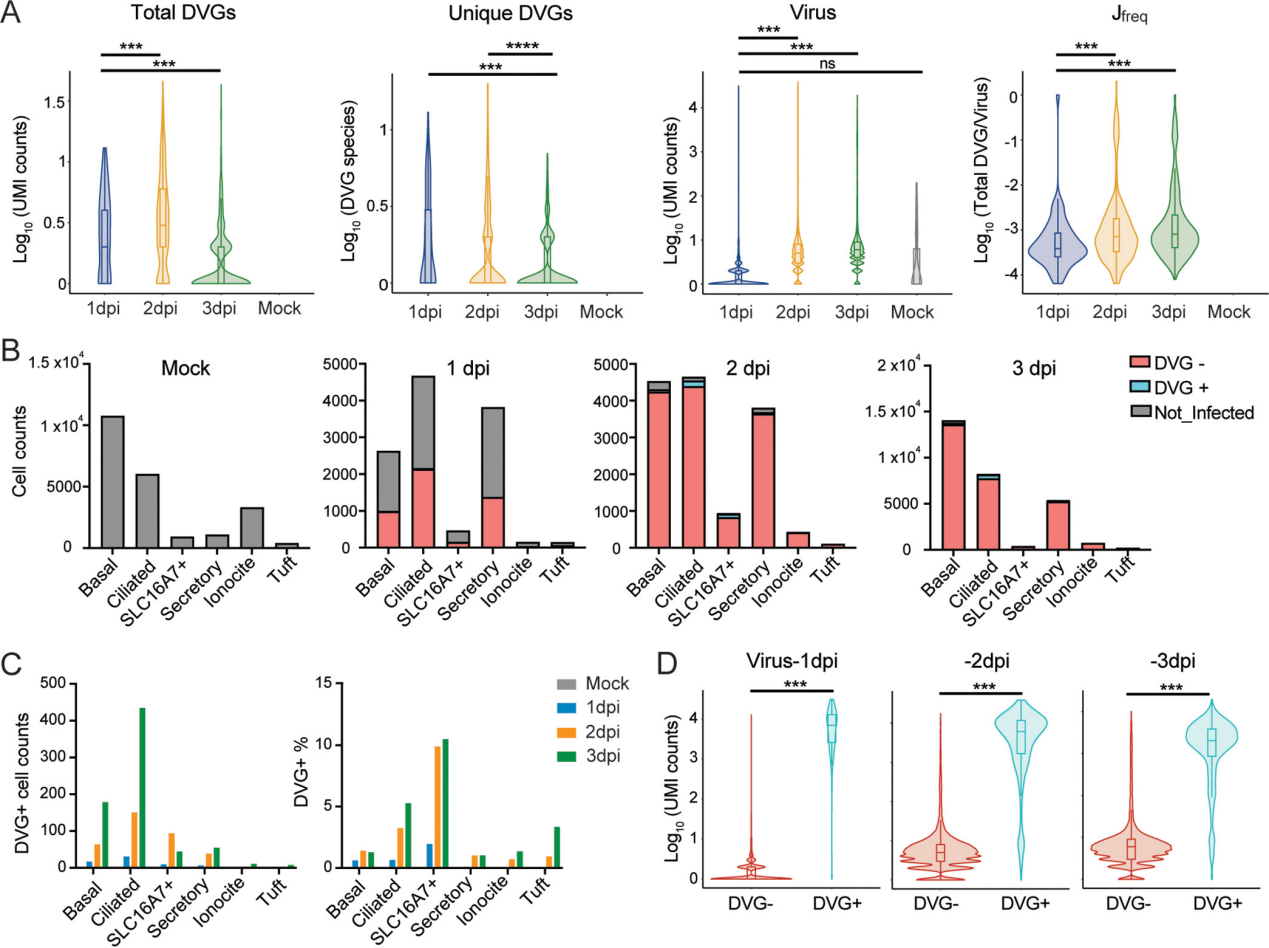
DVG generation in infected PHLE cells from the single-cell level. (A) Violin plots of log-transformed viral UMI counts; total and unique DVG UMI counts; and J_freq_ for 1 dpi, 2 dpi, 3 dpi, and mock samples. (B) Bar plots of cell counts of uninfected cells, DVG^−^ infected cells, and DVG^+^ cells within different cell types for mock, 1 dpi, 2 dpi, 3 dpi samples. Infected cells were cells with viral UMI over 1, and DVG^+^ cells were the ones with DVG UMI over 1. All DVG^+^ cells had at least 1 viral UMI. (C) Bar plots of DVG^+^ cell counts and DVG^+^ percentages per cell type for mock, 1 dpi, 2 dpi, and 3 dpi samples. (D) Violin plots of log-transformed viral counts for DVG^+^ and DVG^−^ virus-positive cells. ***, *P* < 0.01; ****, *P* < 0.001; by two-sided Wilcoxon signed-rank test.

**FIG 6:**
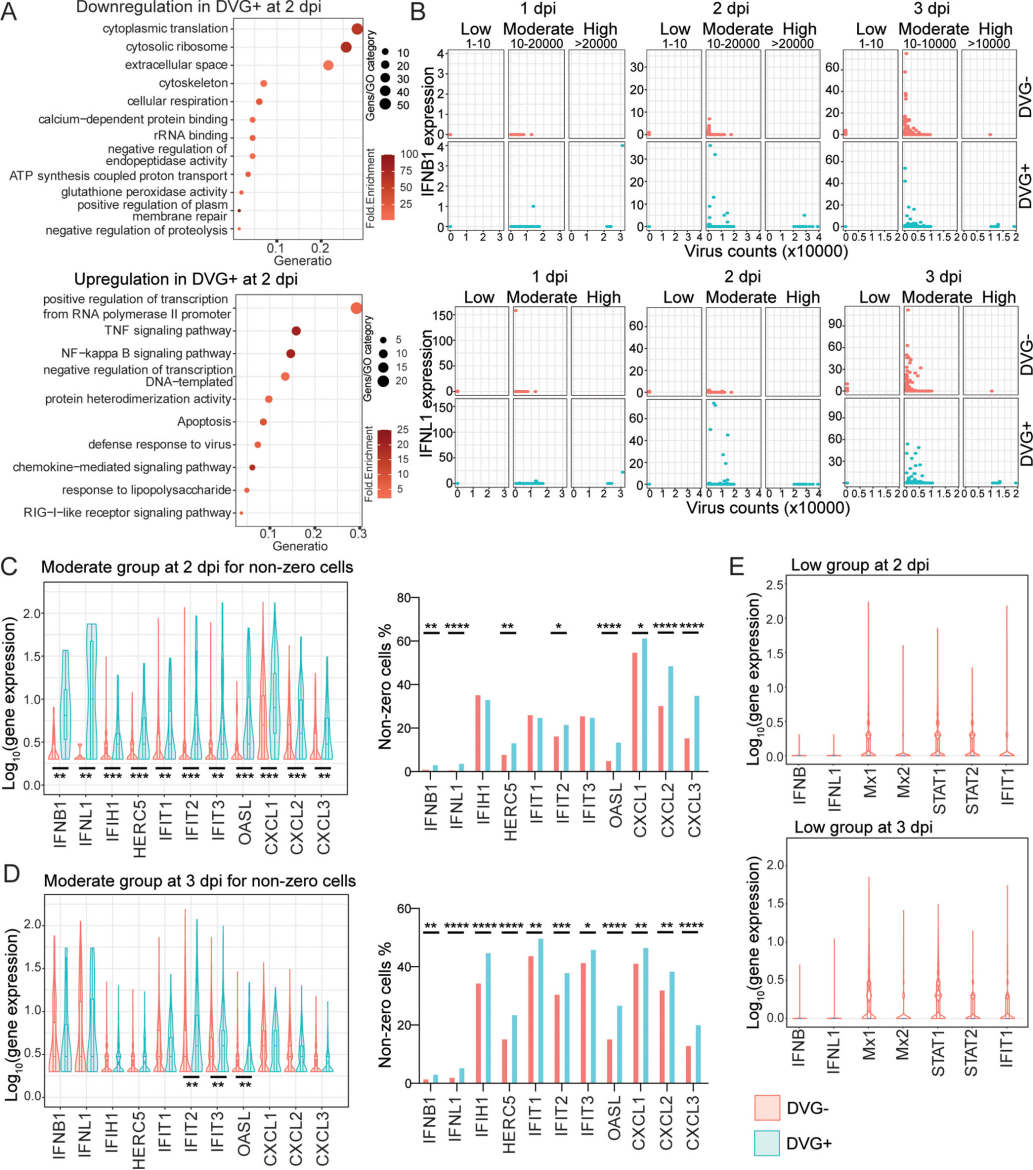
DVG^+^ cells expressed primary IFNs earlier than DVG^−^ cells. (A) Gene Ontology analysis of genes that were downregulated (top) and upregulated (bottom) in DVG^+^ cells relative to DVG^−^ cells at 2 dpi. Circle size represented the number of genes in each pathway. Gene ratio represented the ratio of number of genes in that pathway to the number of genes in the entire cluster. (B) Gene expression of IFNB1 and IFNL1 (*y* axis) was correlated with viral UMI level (*x* axis) within each virus count group. Virus groups with their counts criteria were indicated on top of the graph. Each dot represented individual cells, and they were colored based on their presence of DVGs. (C and D) In the moderate virus group, the expression level of IFNB, IFNL1, selected ISGs, and chemokines for nonzero (gene counts, >0) cells and the percentage of nonzero cells within DVG^+^ and DVG^−^ groups were compared and graphed at 2 dpi (C) and 3 dpi (D). Two-sided Wilcoxon signed-rank test and Fisher’s exact test were used to compare the gene expression level and nonzero cell percentage between DVG^−^ and DVG^+^ groups, respectively; ****, *P* < 0.0001; ***, *P* < 0.001; **, *P* < 0.01; *, *P* < 0.05. (E) Expression level of IFNB, IFNL1, and selected ISGs for DVG^−^ cells within the low virus group at 2 dpi and 3 dpi were graphed as violin plots.

To understand DVG generation and their host responses at the single-cell level, we obtained one single-cell RNA-seq data set using adult PHLE cells with infection at an MOI of 0.01 (GSE166766). Consistent with the previous observations, viral counts, total DVG counts, and J_freq_ at 2 dpi were all significantly increased compared with those at 1 dpi, but they were not significantly different from those at 3 dpi (Fig. 5A), whereas unique DVGs at 3 dpi dropped compared with those at 1 dpi and 2 dpi. Again, a positive correlation between the numbers of total DVGs and unique DVGs was observed in DVG^+^ cells for all time points (Fig. S1D). Note that 51 cells (~0.2% of total mock population) containing viral counts ranging from 1 to 198 were identified in the mock sample. As no DVG counts were identified from those cells, they were excluded from the following DVG analysis. Major cell types enriched with DVGs were ciliated cells, basal cells, and SLC16A7+ cells (red in Fig. 5B, grouping of cell types was based on the markers used in the original publication [47]). Among these three cell types, ciliated cells had the most DVG^+^ cells, whereas SLC16A7+ cells had the highest percentage of DVG^+^ cells (Fig. 5C). All DVG^+^ cells contained at least 1 viral count (virus-positive cells), and total viral counts were significantly higher in DVG^+^ cells than those in DVG^−^ cells at all three time points (Fig. 5D). Only about 1% of virus-positive cells at 1 dpi (*n* = 60) were DVG^+^. Therefore, we focused on the DVG^+^ population at 2 dpi (*n* = 348) and 3 dpi (*n* = 725) to analyze their host responses. Differential expression tests were then performed using three different methods in Seurat (MAST, Wilcox, and DEseq2) between DVG^+^ and DVG^−^ groups within virus-positive cells. Significantly more genes were identified as downregulated in DVG^+^ cells than genes that were upregulated at both time points (adj_pvalue, <0.01; log fold change [FC], >0.25), and similar enriched pathways were observed from GO analysis. Specifically, the ribosomal cytoplasmic translation (host protein synthesis) was largely inhibited in DVG^+^ cells, possibly due to their higher level of viral replication (more expression of NSP1) than DVG^−^ cells (2 dpi in Fig. 6A, top; 3 dpi in Fig. S5C). However, pathways, such as transcription from RNA polymerase II promoter, tumor necrosis factor (TNF), NF-κB, and apoptosis, were significantly enriched in the upregulated genes. Importantly, defense against virus and chemokines were also observed in the upregulation list, consistent with the results from bulk RNA-seq (2 dpi in Fig. 6A, bottom; 3 dpi in Fig. S5D). Next, we specifically examined the expression level of representative genes related to type I/III IFN pathways between DVG^−^ and DVG^+^ viral-positive cells, including two primary IFNs (IFNB1 and IFNL1), ISGs, and chemokines selected from the differentially expressed gene list. To better control viral loads, we further categorized virus-positive cells (cells with virus count, ≥1) based on their viral counts as three groups, as follows: low (viral counts, ≤10), moderate (viral counts, >10 to <20,000 for 1 dpi and 2 dpi; viral counts, >10 to <10,000 for 3 dpi), and high (viral counts, ≥20,000 at 1 dpi and 2 dpi; viral counts, ≥10,000 at 3 dpi). DVGs were identified mostly in moderate (~12%) and high groups (>84%), and an extremely small percentage (<0.2%) of low infected cells generated DVGs. Two primary IFNs were predominantly expressed only in the moderate viral group regardless of DVG presence. However, DVG^+^ cells expressed two primary IFNs 1 day earlier than DVG^−^ cells (2 dpi versus 3 dpi, moderate group in Fig. 6B), suggesting a role of DVGs in stimulating primary IFNs early. In support, ISGs showed a similar trend and the mock showed a basal level of IFN responses when we compared the average expression of all cells from mock, DVG^−^/DVG^+^ in the moderate virus group at either 2 dpi or 3 dpi (Fig. S5E). As IFN-related genes are zero-inflated, we also performed comparisons for both the expression level of cells expressing interested genes (gene counts >0, named as nonzero cells) and their percentages within DVG^+^ and DVG^−^ groups. Briefly, the average expression of ISGs (nonzero cells) was all significantly enhanced in DVG^+^ cells within the moderate group at 2 dpi, but this enhancement was partially lost at 3 dpi despite a higher percentage of DVG^+^ cells expressing IFNs and ISGs at 3 dpi than that of DVG^−^ cells (Fig. 6C and D). Different from the moderate group, the high viral group had a minimal expression of all IFN-related genes, further confirming that IFN pathways were suppressed in highly infected cells (Fig. 6A and B). The low viral group predominantly expressed ISGs rather than two primary IFNs at all time points (Fig. 6E), suggesting they are the secondary responders to initial type I/III IFN production. Taken together, our analysis strongly suggests that DVG^+^ cells with moderate infection were the first responders to viral infection, quickly expressing primary IFNs and subsequently alerting neighboring cells to express ISGs.

### Symptomatic COVID-19 patients had larger amount of and higher J_freq_ of SARS-CoV-2 DVGs than asymptomatic patients.

**FIG 7:**
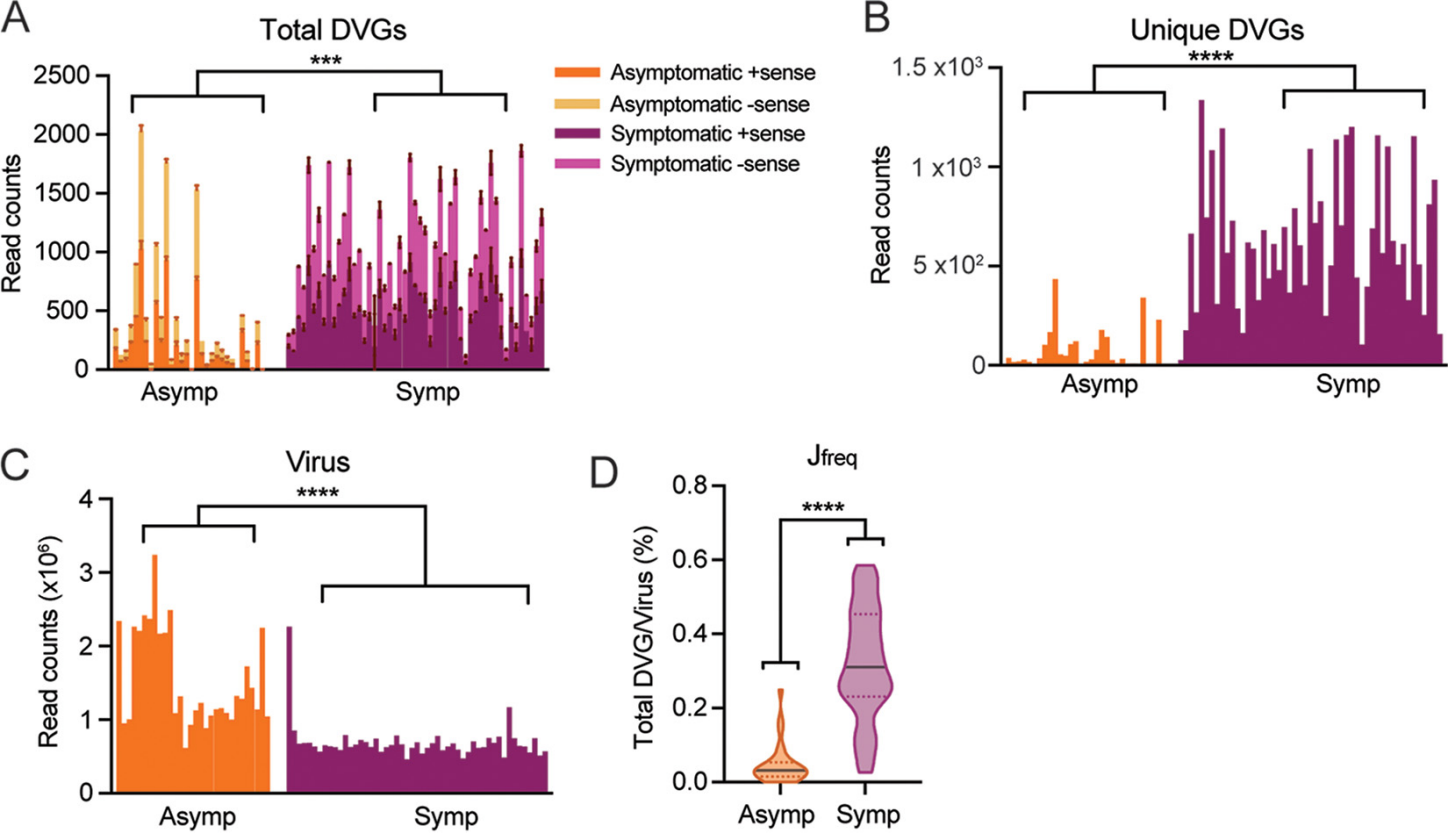
Symptomatic COVID-19 patients had a larger amount of and larger J_freq_ of SARS-CoV-2 DVGs than asymptomatic patients. Samples of various collection methods, including nasopharyngeal (*n* = 42), anterior nasal (*n* = 35), and oropharyngeal (*n* = 5), were used from NGS data set PRJNA690577. Symptomatic samples (*n* = 51) were collected from patients presented at the hospital with symptoms consistent with COVID-19, while asymptomatic samples (*n* = 30) were collected from patients who did not have symptoms consistent with COVID-19 and were found through contact tracing and workforce screening. Total DVG read counts (A), unique DVG read counts (B), viral read counts (C), and J_freq_ (D) percentages were calculated and graphed for all symptomatic and asymptomatic samples. ****, *P* < 0.0001; ***, *P* < 0.001; by two-sided Mann-Whitney U test.

As SARS-CoV-2 DVGs can stimulate the early expression of primary IFNs, the question of whether DVG generation is associated with COVID-19 disease severity was asked. We identified a publicly available NGS data set (PRJNA690577) investigating sgmRNAs and their protein expression from symptomatic versus asymptomatic COVID-19 patients where authors also indicated more deletions with lengths of over 20 nt more in symptomatic patients than those in asymptomatic patients (43). To better examine the DVG (larger deletions) level between two patient groups, we applied our criteria to this data set and found increased total DVG counts (both −sense and +sense, Fig. 7A), increased unique DVG counts (Fig. 7B), and subsequently higher J_freq_ (Fig. 7D) in symptomatic individuals than those in asymptomatic patients on average. Additionally, our method also confirmed the original finding that the read counts for genomic RNA were significantly lower in symptomatic patients than those in asymptomatic patients (Fig. 7C). Interestingly, we observed the positive correlation between total DVGs and unique DVGs only for symptomatic patients but not for asymptomatic patients (Fig. S1E). These data imply the potential role of DVGs in COVID-19 symptom development.

### High DVG J_freq_ was observed in one COVID-19-persistent patient.

**FIG 8:**
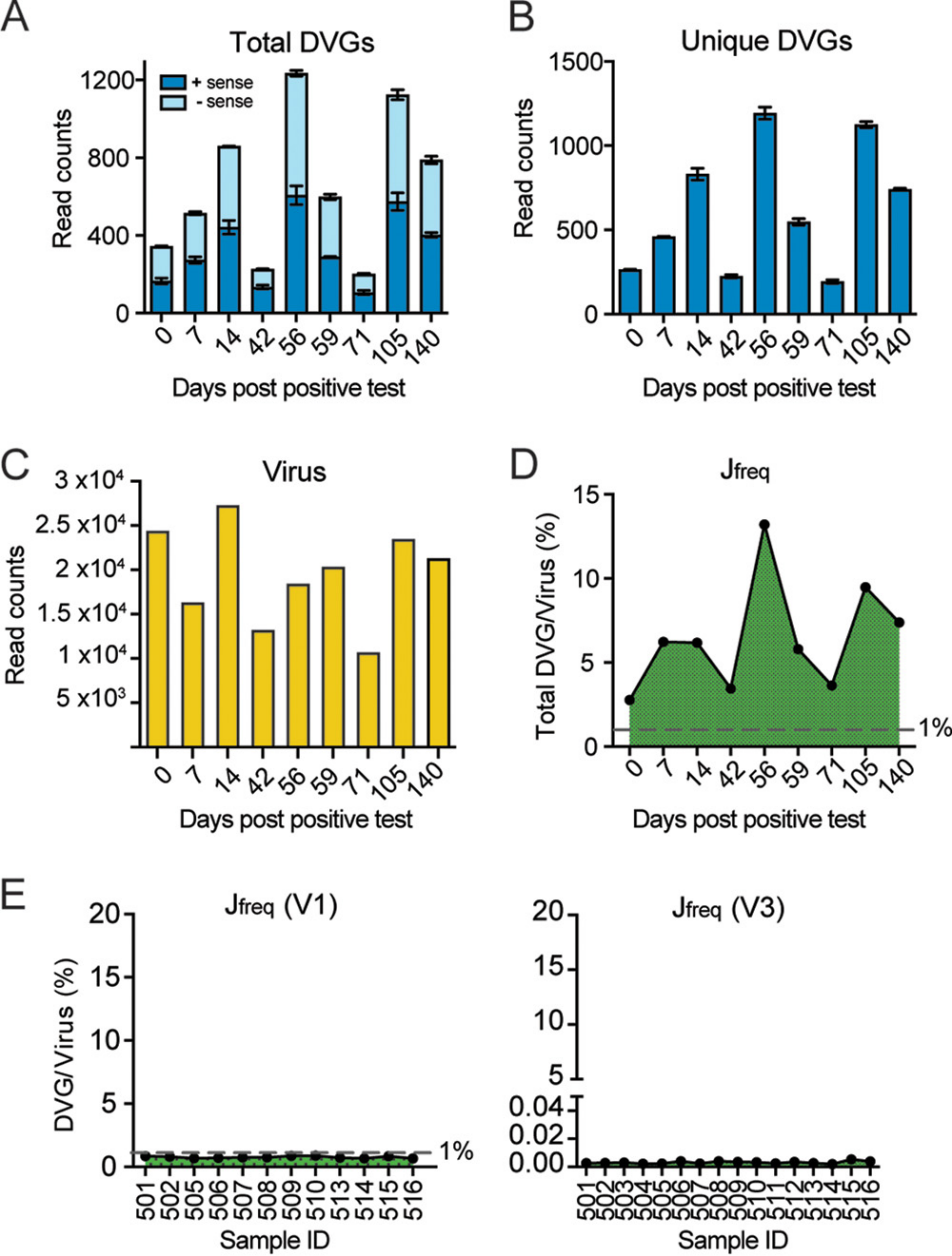
High DVG J_freq_ was observed in one SARS-CoV-2-persistent patient. Nasal samples were collected from one immunosuppressive patient with persistent viral infection at 9 different time points after the patient first tested positive for COVID-19. DVGs were identified from the NGS data set (ERP132087/PRJEB47786) of the nasal samples from this patient. Total DVG read counts (A), unique DVG read counts (B), viral read counts (C), and J_freq_ (D) percentages were calculated and graphed for samples at each time point. (E) J_freq_ of samples in another NGS data set (PRJNA707211) utilizing the same amplification and sequencing methods demonstrated a much smaller J_freq_ than that of the SARS-CoV-2-persistent patient, which was comparable to J_freq_ levels found SARS-CoV-2-infected *in vitro* and autopsy samples. As this NGS is paired end, R1 and R2 were treated as duplicates and error bars stand for mean ± SD.

SARS-CoV-2 can develop persistent infections in immunosuppressive patients (48, 49), and DVGs have been reported to facilitate viral persistence (50). To examine whether DVGs are associated with persistent SARS-CoV-2 infection in patients, we identified one NGS data set, where nasal samples were taken at nine time points from one immunosuppressive patient who was infected with SARS-CoV-2 and was positive for virus up to 140 days since the first hospital admission (PRJEB47786). We detected DVGs in all nine time points, but the amount of total/unique DVGs was not always correlated with total virus counts (Fig. 8A to C). More interestingly, the J_freq_ of DVGs from the samples in this patient was much higher than the number we observed in *in vitro* infections and autopsy tissues (Fig. 8D versus Fig. 1B, 4A, and 5A), with the highest J_freq_ up to nearly 15% at 56 days post-positive test. We also noticed that in this data set, on average, 93% of total DVGs are unique DVGs, which is much higher than all previous data sets (see Fig. S6H in the supplemental material), and the majority of them had only 1 NGS count. Excluding those DVGs with 1 count led to the significant reduction of the numbers for total/unique DVGs, and consequently, J_freq_ dropped to <1%, which was the same level as that observed in primary cells (Fig. S6G). As this high percentage of unique DVGs was unexpected and the method used in this data set (tiled-PCR using ARTIC V3) was different from previous bulk and single cell RNA-seq, we first tested the accuracy of mapping for all DVG reads identified in this NGS via BLAST+. Consistently, ~97% of ViReMa-identified DVG reads were in agreement with BLAST output (see Table S3 in the supplemental material). To test whether this observation was due to the different approach, we found another NGS data set with nasopharyngeal swab samples of normal COVID-19 patients using tiled-PCR (Artic V1 and V3) followed by Illumina sequencing (PRJNA707211). We found that the J_freq_ of each patient sample was below 1% (Fig. 8E), which is within the range of the J_freq_ observed in Fig. 7 (also used tiled-PCR to test clinical samples). We further confirmed that the high percentage of unique DVGs in this data set was not due to the higher coverage or sequencing depth (Fig. S6H). Together, our data suggest that the exceptionally diverse DVG composition (unusually high percentage of unique DVGs) in this patient was not due to the misalignment or amplification and sequencing methods but rather may be associated with the suppression status of patient’s immune system, persistent viral infection, or oropharyngeal samples.

10.1128/mbio.00250-23.6 FIG S6 Functional analysis using DVGs with counts of >1. Repeat of the same analyses as shown in Fig. 4A (A), Fig. 4E (B), Fig. 6B (C), Fig. 6C (D), Fig. 6D (E), Fig. 7 (F), and Fig. 8 (G) using DVGs with counts more than 1 (DVG, >1). For comparisons in D to E, gene expressions were compared by the two-sided Wilcoxon signed-rank test, and nonzero cell percentages were compared by Fisher’s exact test; ****, *P* < 0.0001; ***, *P* < 0.01; **, *P* < 0.05. In F, total and unique DVG numbers were compared by using the two-sided Mann-Whitney U test; ****, *P* < 0.0001. In G, R1 and R2 were treated as duplicates, and error bars stand for mean ± SD. (H) Shows the percentage of unique DVGs among total DVGs, sequencing coverage, and depth for Fig. 1, 4, 7, and 8. The number of total sequencing reads was used for estimation of sequencing depth, and coverage was calculated as (total reads) × (average read length)/genome size. Both host and virus genomes were used to calculate genome size for Fig. 1 and Fig. 4, as these two NGSs included both host and viral reads. The SARS-CoV-2 genome was used to calculate the genome size for Fig. 7 and Fig. 8, as more than 90% of reads in these two sequences were aligned to the viral genome. Download FIG S6, TIF file, 6.3 MB. Copyright © 2023 Zhou et al. 2023 Zhou et al. https://creativecommons.org/licenses/by/4.0/ This content is distributed under the terms of the Creative Commons Attribution 4.0 International license.

10.1128/mbio.00250-23.9 TABLE S3 Summary of validation of DVGs identified in the immunocompromised patient. Download Table S3, DOCX file, 0.02 MB. Copyright © 2023 Zhou et al. 2023 Zhou et al. https://creativecommons.org/licenses/by/4.0/ This content is distributed under the terms of the Creative Commons Attribution 4.0 International license.

## DISCUSSION

It has been well-documented that DVGs are universally generated across single-stranded RNA viruses, both *in vitro* and *in vivo*, such as respiratory syncytial virus (RSV), measles virus, influenza virus, Ebola virus, dengue virus, CoVs, and many more. For SARS-CoV-2, DVGs result from nonhomologous recombination and have been observed previously in infected Vero cells (40) and nasal samples of COVID-19 patients (42). In Vero cells, SARS-CoV-2 is reported to be more than 10 times more recombinogenic than other CoVs, such as MERS-CoV (17), and junctions of SARS-CoV-2 DVGs are most commonly flanked at U-rich RNA sequences, suggesting a novel mechanism by which viral polymerases use to generate DVGs. Interestingly, recombination is also proposed to be critical for coronavirus diversity and emergence of SARS-CoV-2 and other zoonotic CoVs. To further understand the recombination positions of SARS-CoV-2 DVGs, we expanded DVG analyses to 4 more commonly used cells lines for SARS-CoV-2 studies, primary human lung epithelial (PHLE) cells, and autopsy tissues from patients who died of complications of COVID-19, further confirming that DVGs are produced ubiquitously during SARS-CoV-2 infections. Interestingly, the numbers of total DVGs and unique DVG junctions are always positively correlated in our analyzed NGS data sets (with the exception of asymptomatic patients, Fig. S1), including the single-cell RNA-seq (scRNA-seq) analysis, in which PCR duplicates were identified by unique molecular identifier (UMI) and then eliminated from the downstream analysis. This information further implies that generating more DVG species at the initial production step contributes to a higher overall DVG amount. Additionally, we observed strong positive correlations between total/unique DVGs and viral counts for autopsy tissues, indicating that both total and unique DVG amounts are dependent on viral load or viral infection level. This idea is consistent with our analyses in Fig. 4 to 6, where DVG^+^ cells (DVG-high samples) have higher viral load than DVG^−^ cells (DVG-low samples). But this correlation was not observed for *in vitro* infections (Fig. 1D). We think that this finding is because viral counts for autopsy tissues (all from one study and one RNA-seq) better represented the actual viral load, as indicated in the original publication, whereas *in vitro* infections compared in Fig. 1 were conducted from 3 different labs using different methods for library preparation and having different sequencing depths. Thus, the discrepancy of viral counts among three *in vitro* infections may not reflect the true differences of viral load in those samples. Furthermore, J_freq_ was not significantly correlated with virus counts. While J_freq_ can be used to normalize infection level and sequencing depth, it estimates how frequently viral polymerase generates DVGs during viral infection. We think that it is the intrinsic properties of viral polymerase and/or host factors (for example, mutations/factors changing the recombinogenic property of viral polymerase) that contribute more to this ratio rather than the viral load. It is noted that we used total DVG amount, instead of J_freq_, as the criteria for functional analyses, for two reasons. (i) We found that J_freq_ cannot always represent the true total DVG amount, which is especially true for the single-cell analysis. When we calculated the J_freq_ for the low-virus group with the viral counts less than 10, those cells typically have bizarrely high J_freq_, which is due to their very low viral counts but not due to their DVG counts. In fact, most of those cells only have 1 DVG count. This issue is less problematic for bulk RNA-seq analyses since the total viral counts for infected samples are typically above 10^3^. (ii) We found that the number of total DVGs (likely the unique DVGs as well, as these two parameters are positive correlated in most cases), rather than J_freq_, matters for their IFN stimulation function. Samples with abundant total DVG counts, rather than high J_freq_, have enhanced type I/III IFN responses as shown in Fig. 4. However, DVG-high samples also have a higher viral load than DVG-low samples. To take into consideration the wild-type (WT) virus abundance and specifically look at the DVG function, we chose 4 samples (donor 1, donor 2, donor 4, and donor 5) all at 72 hpi that have similar WT viral abundance but a different total/unique DVG amount (Fig. 4E), and we compared their IFN responses. Consistently, we observed that donor 4 had the most abundant DVGs in both total and unique amounts among the four samples and also had the most greatly induced IFN responses (Fig. 4C); this trend was specific to type I/III IFN responses (Fig. 4D). This result was further verified by qPCR (Fig. 4F), strongly suggesting that SARS-CoV-2 DVGs induced type I/III IFN responses in addition to WT virus.

Importantly, we identified specific genomic hot spots for DVG recombinant sites that are not only consistent in *in vitro* and in patient samples but also shared between +sense and −sense DVGs. These results imply two points, as follows: (i) DVG recombination is not random in SARS-CoV-2 and certain mechanisms are utilized to regulate their production, and (ii) our identified +sense DVGs and −sense DVGs are correlated with each other, likely due to the self-replication in between. Alternatively, certain common mechanisms, such as the secondary RNA structures within viral genomes, result in such correlated junction positions of +sense and −sense DVGs. One limitation of our analyses using short-read NGS is that short reads are <400 bp long, and thus, junction reads are less likely to cover the entire DVG sequence. More analyses from long-read sequencing data are needed to further confirm the full sequences of DVGs. Based on the secondary structures identified by COMRADES cross-linking in the +sense viral genome (44), we calculated the structural distance between two recombination sites of any −sense DVGs and surprisingly found an association between DVG breakpoints and rejoin points with a short structural distance (Fig. 3C and D), as mediated by RNA base pairing. The relatively short structural distance, compared with the sequence length, indicates that DVGs form when the viral polymerase falls off the template during replication and then rejoins the viral template at a position close in space, which can be quite distant in sequence. This relatively short structural distance between two DVG junction sites strongly suggests that the recombination of the viral polymerase complex can be guided by the secondary structures within the +sense viral genome. Unfortunately, none of the NGS data sets used here is derived from strand-specific sequencing, and thus, we cannot obtain the strand situation of DVGs in the original RNA samples. However, as the structures formed within the −sense strand are expected to be different from those in the +sense strand (because folding stability is strand direction dependent and G-U pairs map to A-C mismatches in the complementary strand), we postulate that DVG generation is initiated as −sense by the viral polymerase complex using +sense viral genomes as the template and −sense DVGs are then used as the templates to replicate +sense DVGs. More investigations on the secondary structures in both strands of viral genomes and their role in viral recombination are needed to further test this hypothesis.

The effects of the presence of DVGs on host response and viral replication were additionally explored. It was observed that samples with moderate and large amounts of DVGs exhibited enhanced antiviral responses than samples with small amounts of DVGs (Fig. 4). From scRNA-seq analysis, IFN pathways were suppressed in highly infected cells and primary IFNs were stimulated earlier in moderately infected cells with DVGs than the ones without DVGs (Fig. 6). Note that these two NGS data sets used different MOIs for infection in primary lung cells, but we observed similar IFN stimulation and similar J_freq_. These findings suggest that different MOIs will impact the overall infection level and the total amount of DVGs, with higher MOIs yielding more infected cells and more DVGs. However, MOI has little impact on the J_freq_ of DVGs (consistent with the result in Fig. 1 that no correlation exists between J_freq_ and viral counts), and regardless of the initial MOI for infections, infected cells with enough DVGs accumulated and with a moderate infection level will stimulate a robust type I/III IFN response compared with the infected cells without DVGs. The mechanisms by which DVGs enhance IFN responses are unknown. DVGs from RSV and influenza virus can function as primary triggers to directly stimulate type I IFN production through RIG-I like receptors (50). It was reported previously that SARS-CoV-2 RNAs can be recognized by MDA5 (51, 52), and we showed that the expression of MDA5 (IFIH1) was elevated in DVG^+^ cells at 2 dpi (Fig. 6C). Therefore, it is possible that SARS-CoV-2 DVGs stimulate type I/III IFNs through MDA5. Alternatively, if DVGs do not directly stimulate IFN production, they can suppress the expression of viral-encoding IFN antagonists by large deletions, resulting in an earlier and higher IFN expression in DVG^+^ cells. Indeed, IFN antagonists are encoded in NSP1, NSP3, NSP5, NSP12, NSP13, NSP14, NSP15, ORF3a, ORF3b, ORF6, ORF7a, ORF7b, ORF8, ORF9b, N, and M (51, 53–56), and most of them are within the deletion regions based on our conserved genomic hot spots for DVG recombination sites (Fig. 2A and B). Nevertheless, the higher IFN expression in DVG^+^ samples/cells suggest the critical role of DVGs in modulating host responses and the subsequent disease severity of COVID-19.

To further explore the role of DVGs in COVID-19 severity, we took advantage of one published NGS data set that investigated sgmRNA levels in patients with differing clinical severities (43). They observed a reduction of viral sgmRNAs and viral deletions larger than 20 nt but an increased viral genomic RNA level in nasal samples from asymptomatic patients. We applied our criteria to this data set and found that the abundance and J_freq_ of DVGs containing deletions larger than 100 nt were similarly reduced in asymptomatic patients compared with those of symptomatic patients. The significant difference in DVG production between patients with and without symptoms leads us to posit that the quantity and J_freq_ of DVGs contribute to the heterogeneity of both disease outcomes and the presentation of symptoms in infected individuals, potentially through modulating host immune responses. As sgmRNAs and DVGs were both reduced in the asymptomatic group in this cohort study, we wondered whether sgmRNA production is always positively correlated with DVG generation. To examine this, we quantified TRS-dependent junction reads (recombination sites, <85) from the ViReMa output in infected PHLE cells from different age groups as the estimation of sgmRNAs (data set used in Fig. 4). Interestingly, we did not observe any positive correlation. Specifically, D198 had the least DVG amount among all samples at 72 hpi and had more sgmRNA counts (*n* = 385) than D239 (*n* = 32), which again confirms that DVGs, rather than sgmRNAs, specifically stimulate IFN responses. We wonder why do symptomatic patients generate more DVGs? It is possible that the IFN response induced by DVGs lead to the subsequential expression of cytokines, such as interleukin-6 (IL-6), which is known to be an important mediator for immune-induced fever, as shown in blood monocytes for SARS-CoV-2 infection (57). However, a rapid and controlled immune response will lead to milder symptoms, whereas a prolonged and uncontrolled immune response will lead to severe symptoms and even death (5). Future studies with higher symptom scoring resolution, such as mild/moderate, severe, and death, could elucidate the potential associations of DVG abundance and/or frequency with viral load, IFN responses, and COVID-19 disease severity.

DVGs have been shown to promote viral persistence for various viruses, such as influenza A (26), dengue (58), Japanese encephalitis virus (59), mumps (60), rabies (61), Sendai (62), and measles (63); additionally, worse disease outcome was found to be associated with prolonged DVG detection in RSV (64). We examined the relationship between DVGs and SARS-CoV-2 persistent infection, and we were surprised to find one immunosuppressed patient generating DVGs consistently in every collected time point over a period of 140 days with a very diverse DVG population at each time point. Especially, we noticed that the majority of DVGs had only 1 NGS count, suggesting an exceptionally diverse rather than an enriched DVG population compared with all other analyzed data sets. This observation was not due to a misalignment of the algorithm or the amplification and sequencing methods, but it is likely a biological difference from a compromised immune status or a prolonged viral infection. These data additionally imply that a prolonged DVG presence/production may associate with a prolonged viral infection and a longer length of illness. More longitudinal studies are needed to elucidate the relationship between DVGs and prolonged viral infection especially in immunosuppressed COVID-19 patients.

We used ViReMa to identify DVGs, as this algorithm has been developed and validated to faithfully identify recombination events in a range of plant viruses (65–67), insect viruses (68, 69), arthropod-borne viruses (arboviruses) (70–72), human pathogens (influenza virus [73], reovirus [74], MERS-CoV, and SARS-CoV-2 [17, 75]), and retroviruses (human immunodeficiency virus [HIV]) (76). It has also been validated independently by Alnaji et al. (77) and Boussier et al. (78) who provided a series of condition and parameter optimizations using simulated and experimental data to map RNA recombination events in DVGs of influenza A and B. Based on our own validation (Fig. S3 for standard bulk RNA-seq and Table S3 for tiled-PCR bulk RNA-seq), ~97% of ViReMa-identified reads were in agreement with BLAST results, including the ones with only 1 count, making us confident to include them for all analyses. However, the following two concerns were raised for DVGs with only 1 count: (i) whether DVG junction reads with very few counts are artefactual recombination caused during library prep and are nonexistent in the original RNA sample and (ii) whether DVG species with only 1 count or cells with only 1 DVG count have biological function. scRNA-seq uses UMI to distinguish PCR duplicates and thus can better represent the real situation in original RNA sample. From our scRNA-seq analysis, we observed a number of cells with only 1 count of a DVG junction read, but most of those cells did not express the examined IFN-related genes, strongly suggesting that DVGs with only 1 count per cell do exist in a real viral infection, but they are not enough to stimulate type I/III IFN responses. To make sure our conclusions were not driven by DVGs with 1 NGS count, we repeated our major functional analyses for DVGs with counts of >1, and again, all the conclusions remained valid (Fig. S6). More experiments will be needed to examine the amount of total DVGs or which DVG species are sufficient to stimulate type I/III IFN responses.

Determination of the generation (recombination) and function of DVGs during SARS-CoV-2 infection would facilitate the reduction of the viral recombination events, which greatly contributes to newly emerging CoVs, and elucidates another point of mitigating disease severity from those infected. We showed here that the recombination sites of SARS-CoV-2 DVGs are clustered in several genomic regions, which are likely to be determined by RNA secondary structures formed in between. Furthermore, our studies provide the evidence that DVGs play vital roles in IFN stimulation, prolonged viral replication, and symptom development during SARS-CoV-2 infection, urging for more investigations to further determine the mechanism of DVG generation and their impact on SARS-CoV-2 pathogenesis.

## MATERIALS AND METHODS

### Virus and cell preparation.

The following reagent was deposited by the Centers for Disease Control and Prevention and was obtained through BEI Resources, NIAID, NIH: SARS-related coronavirus 2, isolate USA-WA1/2020, NR-52281. SARS-CoV-2 was propagated using African green monkey kidney epithelial Vero E6 cells (American Type Culture Collection; CRL-1586) in Eagle’s minimum essential medium (Lonza; 12-125Q) supplemented with 2% fetal bovine serum (FBS) (Atlanta Biologicals), 2 mM l-glutamine (Lonza; BE17-605E), and 1% penicillin (100 U/mL) and streptomycin (100 μg/mL); the titer of the virus was also determined with this cell line and medium. Viral stocks were stored at −80°C. All work involving infectious SARS-CoV-2 was performed in the biosafety level 3 (BSL-3) core facility of the University of Rochester, under institutional biosafety committee (IBC) oversight.

### PHLE culture on an air-liquid interface and SARS-CoV-2 infection.

Primary human lung epithelial (PHLE) cells were cultured on an air-liquid interface (ALI) as described previously (79, 80). Briefly, lung tissue issues were digested with a protease cocktail, including collagenase type A from *Clostridium* (2 mg/mL), dispase II (1 mg/mL), elastase (twice crystallized from porcine pancreas; 0.5 mg/mL), and DNase-I from bovine pancreas (2 mg/mL). Cells were then cultured on a collagen-coated transwell plate (Corning; 3470) until each well reached a transepithelial electrical resistance (TEER) measurement of >300 Ω. Cells were then placed on an ALI by removing media from the apical layer of the transwell chamber and continuing to feed cells on the basolateral layer as they differentiated. Cells were differentiated for 4 to 5 weeks at ALI before used in experiments. The apical layer of primary lung cells that had been cultured on an air-liquid interface for about 4 to 5 weeks was inoculated with SARS-CoV-2 (SARS-CoV-2/human/HKG/VM20001061/2020) at an MOI of 5 (titer was determined in Vero E6 cells) in phosphate-buffered saline containing calcium and magnesium (PBS++; Gibco; 14040-133) and incubated at 37°C for 1.5 h. The infectious solution was then removed, and the apical layer was washed with PBS++. Cells were then incubated for 24, 48, or 72 h.

### SARS-CoV-2 inactivation, RNA preparation, and qPCR.

Cells that were harvested at 24 and 72 h postinfection were lysed with SDS lysis buffer (50 mM Tris pH 8.0, 10 mM EDTA, and 1% SDS) and collected with a wide-bore pipette tip. Cells that were harvested at 48 h were first washed by dispensing and aspirating a 37°C HEPES-buffered saline solution (Lonza, CC-5022) and then were trypsinized with 0.025% trypsin-EDTA (Lonza; CC-5012) for 10 min at 37°C. Dissociated cells were aspirated using a wide-bore pipette tip to a tube containing ice-cold trypsin neutralization solution (Lonza; CC5002); this procedure was repeated to maximize cell collection. Cells were then pelleted by centrifugation, resuspended in chilled HEPES, and centrifugally pelleted once more before being resuspended in SDS lysis buffer. All samples were physically lysed with QIAshredder homogenizers (Qiagen; 79656) and stored at −80°C. Homogenized SDS lysates were diluted 1:1 with RNA lysis buffer (Agilent), and RNA was extracted using the Absolutely RNA microprep kit (Agilent) according to the manufacturer’s protocol, including on-column DNase treatment. A total of 250 ng of total RNA from selected samples was used to perform reverse transcription (iScript; Bio-Rad) followed by qPCR to examine the expression level of RSAD2 (forward [F], 5′-TTGGACATTCTCGCTATCTCCT-3′; reverse [R], 5′-AGTGCTTTGATCTGTTCCGTC-3′), IFIT1 (F, 5′-GCGCTGGGTATGCGATCTC-3′; R, 5′-CAGCCTGCCTTAGGGGAAG-3′), and IFIT2 (F, 5′-GACACGGTTAAAGTGTGGAGG-3′; R, 5′-TCCAGACGGTAGCTTGCTATT-3′). The relative copy numbers of all genes were relative to the housekeeping gene PP1A (F, 5′-GGAATGGCAAGACCAGCAAG-3′; R, 5′-CGAGTTGTCCACAGTCAGCAA-3′).

### Bulk RNA sequencing of infected PHLE cells.

RNA concentration was determined with the 1000 spectrophotometer (NanoDrop, Wilmington, DE), and RNA quality was assessed with the bioanalyzer 2100 (Agilent, Santa Clara, CA). Next, 1 ng of total RNA was preamplified with the SMARTer ultra-low input kit v4 (Clontech, Mountain View, CA) per the manufacturer’s recommendations. The quantity and quality of the subsequent cDNA were determined using the Qubit fluorometer (Life Technologies, Carlsbad, CA) and the bioanalyzer 2100 (Agilent). A total of 150 pg of cDNA was used to generate Illumina-compatible sequencing libraries with the NexteraXT library preparation kit (Illumina, San Diego, CA) per the manufacturer’s protocols. The amplified libraries were hybridized to the Illumina flow cell and sequenced using the NovaSeq6000 sequencer (Illumina). Single-end reads of 100 nt were generated for each sample.

### Bulk RNA-seq data processing and DVG identification.

The data sets used for bulk RNA-seq analyses in Fig. 1 and Fig. 2 are publicly available. Their detailed information was listed in Table S1. The RNA-seq used in Fig. 4 is from our own infection following the protocol as demonstrated earlier. For each sample, we first used Bowtie 2 (v2.2.9) (81) to align the reads to the GRCh38 human reference genome. The unmapped reads were then applied to ViReMa (v0.21) to identify recombination junction sites and their corresponding read counts using a SARS-CoV-2 reference genome (GenBank identifier [ID] MT020881.1). A custom filtering script was developed in R to identify junction reads that met our criteria (R v4.1.0 and RStudio v1.4.17) (script in Text S1). We required that the positions of both sites (break and rejoin) of junction reads were larger than 85, as TRS-L is reported to be located with the first 85 nt of the SARS-CoV-2 genome. Additionally, we required deletions longer than 100 nt to distinguish them from the small deletions often observed in Vero E6 infection/passaging or in variance of concerns. We also included all deletions that had one or more reads as identified by ViReMa. The number of viral reads in each bulk RNA-seq sample was quantified using the RSubread Bioconductor package. The junction frequency (J_freq_) was calculated as shown below for each sample.

$${\text{J}}_{\text{freq}}=\frac{\text{DVG count}}{\text{viral read count}}$$

For host transcriptome analysis, raw fastq files were mapped to the human transcriptome (cDNA; Ensembl release 86) using Kallisto (82) with 60 bootstraps per sample. Annotation and summarization of transcripts to genes were carried out in R, using the TxImport package (83). Differentially expressed genes (≥2-fold and ≤ 1% false discovery rate) were identified by linear modeling and Bayesian statistics using the VOOM function in the limma package (84). Gene Ontology (GO) was performed using the Database for Annotation, Visualization and Integrated Discovery (DAVID) (85).

### DVG identification from the scRNA-seq data set.

We used the publicly available data set from Ravindra et al. (47) accessed through the NCBI database (GSE166766). This study consisted of single-cell RNA-seq (scRNA-seq) data from PHLE cells infected with SARS-CoV-2 that were harvested at 1 day postinfection (dpi), 2 dpi, and 3 dpi. We first used Cell Ranger (86) to construct gene expression matrices for each sample. To identify the number of viral transcripts, the SARS-CoV-2 reference sequence was concatenated to the end of the human genome reference as one additional gene (47). The gene expression matrices were then loaded into the Seurat package in R (87), followed by principal-component analysis and cell clustering. Cells were then clustered and annotated based on the gene markers used in the original publication of this data set. To identify DVGs, we first used UMI-tools (88) to associate the cell barcodes and UMIs with each corresponding read name. Similar to the bulk RNA-seq analysis, we used Bowtie 2 (81), ViReMa, and a custom R filtering script for DVG identification (details in Text S1). We then used the filtered ViReMa output to requantify DVG count based on the UMIs associated with each cell barcode. Specifically, we counted the number of unique UMIs with the same cell barcode as the total number of DVG reads per cell and counted the number of unique UMIs with unique junction positions and the same cell barcode as the unique DVG counts per cell. We also calculated J_freq_ for each cell by using DVG UMI/viral UMI per cell barcode. The numbers of DVG UMIs and J_freq_ of each cell barcode were then added to the gene expression matrix created by Cell Ranger. The J_freq_ values were multiplied by 10^3^ so that they would not be left out during the cell clustering and type identification steps. Cells with more than one DVG UMI (virus positive cells) were grouped as DVG^+^ and DVG^−^ based on the presence or absence of DVG UMI, respectively.

### Differentially expressed genes between DVG^+^ and DVG^−^ in scRNA-seq analysis.

The list of differentially expressed genes between the DVG^+^ group and DVG^−^ group was generated with the Seurat function FindMarkers, after normalizing and scaling the data with the Seurat function SCTransform. Three different types of tests were used to create three differential gene expression (DGE) lists for both 2 dpi and 3 dpi, namely, Mast, DESeq2, and the Wilcoxon signed-rank test (default) using the criteria of percentage of cells where the gene was detected (pct) at >0.1, the adj_pval was <0.01, and the log fold change was >0.25. The final DGE list was determined based on common genes that were found in two of the three methods. To identify the pathways enriched in the DGE list, we first divided the DGE list based on their upregulation and downregulation in DVG^+^ group. GO analysis was performed for the upregulated genes and downregulated genes separately through the DAVID functional annotation clustering tool and graphed in R using the code in Text S1. We then specifically focused on interferon responses between DVG^+^ and DVG^−^ groups. Low, medium, and high groups were further categorized based on their amount of viral UMI within virus-positive cells, and the expression of selected IFN-related genes as specifically compared and graphed between DVG^+^ and DVG^−^ cells within each viral groups in R (code in Text S1).

### DVG identification from the tiled-PCR deep sequencing.

The protocol for identifying DVGs in three publicly available data sets that utilize PCR tiling from Artic LoCost (V1 or V3) (https://artic.network) primer sets followed bulk sequencing data processing for DVG identification. The first data set was used to study DVG generation during longitudinal COVID-19 persistence in one immunosuppressed patient (ENA no. ERP132087, NCBI SRA no. PRJEB47786). To rule out the possibility that observations in the immunosuppressed patient are due to the tiled-PCR approach, we analyzed the second data set (PRJNA707211) containing samples from 16 regular COVID-19 patients that were conducted using the same approach of the first data set. The third one was used to study DVGs in a cohort of both asymptomatic and symptomatic COVID-19 patients (NCBI SRA no. PRJNA690577). This method of amplification produced overlapping 400-bp amplicons that are then used to construct respective sequencing libraries from which data processing and subsequent analysis can occur. For the longitudinal study, the Artic V3 amplicons were sequenced as paired-end 300-bp reads on an Illumina MiSeq instrument. The Artic V3 amplicons of the symptomatic cohort study was PCR amplified by five cycles and also sequenced identically.

### DVG specific RT-PCR.

Total RNA was extracted from Vero E6 cells of both mock and infected treatments at 72 hpi by TRIzol (Invitrogen) according to the manufacturer’s instruction. Isolated total RNA was reversed transcribed using the tSuperScript III first-strand synthesis system (Invitrogen). Based on the hot spots identified by the RNA-seq data analysis, we specifically designed one primer pair targeting hot spot A (SC-R1, 5′-ATTAGGGCTCTTCCATATAGGC-3′; 539F, 5′-CTCGAAGGCATTCAGTACGG-3′). RNAs were first reverse transcribed using SC-R1, and copy-back DVGs (cbDVGs) were then amplified using SC-R1 and 539F.

### Validation of ViReMa identified DVGs by BLAST+.

DVG reads were first identified by ViReMa and R filtration. To validate them, DVG read sequences were specifically extracted from fastaq or bam files based on their Read_ID (in the output of ViReMa), followed by a BLAST+ search (command: blastn -query query-seq.fa -db MT020881 -evalue 1e-10 -num_threads 4 -max_target_seqs 3 -outfmt 6 -out query_results.blast). The orientation of reads was first compared between ViReMa output and BLAST output, and no errors were identified. Then junction positions were compared. First, in BLAST output, the aligned length was compared with the read length for each read. If the difference was less than 10 nt, this read was considered a nonjunction read and thus considered wrong. Junction positions were then compared between ViReMa output and BLAST output for remaining reads. If either side (either break or rejoin) of recombination sites was the same between the two outputs, this read was considered an exact match. Otherwise, this read was considered a DVG with ambiguous junctions. If the ambiguous region was less than 10 nt, we still considered this DVG read verified, and if it was larger than 10 nt, we considered the DVG as having nonaccurate junctions. We excluded the latter when calculating the accuracy rate of ViReMa.

### Secondary structure analysis of DVG junction positions.

Our definition of structural distance follows (46). For a given primary sequence and a corresponding secondary structure, we first convert them to a graph where each nucleotide i is a node. We add an edge (i, i + 1) between any two adjacent nucleotides i and i + 1 (gray bonds in Fig. 3B) and an edge (i, j) between any paired bases i and j (black bonds in Fig. 3B) as reported by Ziv et al. (44) from their COMRADES mapping. This graph can model alternative base pairs. For example, if nucleotide i has possible pairs with nucleotides j, k, and l, then node i will connect five edges (i, i − 1), (i, i + 1), (i, j), (i, k), and (i, l). Based on the connected graph, the structural distance between two nucleotides is formalized as the number of edges in the shortest path between them (red solid path in Fig. 3B), which can be solved by classical graph algorithms (89).

The chimeric reads detected by COMRADES from reference 45 consist of only left- and right-side sequences without base-pairing information. For short-range interactions, they extracted a (continuous) subsequence between the 5′ end of the left side and the 3′ end of the right side and used RNAfold (90) to predict structures for that subsequence. For long-range interactions, they utilized RNAduplex (90) to predict interactions between the two (distant) segments, which does not model any intrasegmental base pairs for either segment. Note that alternative base pairs exist in the data. Therefore, we built the graph based on the predicted base pairs data from in Ziv et al. (44) and calculated the structural distance between any two positions using the method described above. Additionally, we chose a cutoff value of 50 for the number of chimeric reads, which leads to a balanced precision and sensitivity evaluated on the known structure (44).

### Statistical analysis.

Pearson’s correlation was performed to identify the association between virus and DVG counts and virus and J_freq_ in the bulk RNA-seq data sets. For the scRNA-seq data set, unpaired two-sided Wilcoxon signed-rank tests were performed to identify the differences in viral load, DVG counts, and J_freq_ among mock, 1 dpi, 2 dpi, and 3 dpi samples. We first log transformed viral UMI counts and the expression level of selected IFN-related genes and then compared between DVG^−^ and DVG^+^ cells for each time point using unpaired two-sided Wilcoxon signed-rank tests.

### Data availability.

Source data for the publicly available NGS data sets described in the manuscript are available as Table S1. All NGS data sets were retrieved with NCBI and ENA accession numbers GSE147507 (91), GSE148729 (92), BioProject PRJNA628043 (93), GSE166766 (47), GSE150316 (94), BioProject PRJNA707211 (75), BioProject PRJNA690577 (43), ERP132087, and PRJEB47786 (95). The data set of infected samples used in Fig. 4 was deposited at GEO (GSE222381) and is available upon request.
