# Supplementary material for: Generation and Functional Analysis of Defective Viral Genomes during SARS-CoV-2 Infection
Source: mBio. 2023 Apr 19;14(3):e00250-23. doi: 10.1128/mbio.00250-23 (PMC10294654; doi:10.1128/mbio.00250-23)
Supplement: TABLE S1 [file mbio.00250-23-s0007.docx]

**Table S1 Summary of all samples from published datasets**

| Cells/Tissues/Sample Types | Infection Type | MOI | Time Points | Dataset# (Sample#) | Sequence Method | Paired or Single end |
| --- | --- | --- | --- | --- | --- | --- |
| A549-ACE2  Fig. 1B, D, & E  Fig. 2 | In vitro (infected cells) | 2 | 24h | GSE147507 (GSM4486160, 4486161, 4486162) | Bulk | Single |
| NHBE  Fig. 1B, D, & E  Fig. 2 | In vitro (infected cells) | 2 | 24h | GSE147507 (GSM4432381, 4432382, 4432383) | Bulk | Single |
| Calu3_total RNA  Fig. 1B, D, & E  Fig. 2 | In vitro (infected cells) | 0.3 | 24h | GSE148729 (GSM4477962, 4477963) | Bulk | Paired |
| Calu3_polyA  Fig. 1B, D, & E  Fig. 2 | In vitro (infected cells) | 0.3 | 24h | GSE148729 (GSM4477910, 4477911) | Bulk | Single |
| Caco2  Fig. 1B, D, & E  Fig. 2 | In vitro (infected cells) | 0.3 | 24h | GSE148729 (GSM4477888, 4477889) | Bulk | Single |
| H1299  Fig. 1B, D, & E  Fig. 2 | In vitro (infected cells) | 0.3 | 24h | GSE148729 (GSM4477868, 4477869) | Bulk | Single |
| Vero E6_S  Fig. 1B, D, & E  Fig. 2 | In vitro (supernatants) | 3 | 48h/  passage | PRJNA628043:  SRP258466 | Bulk | Paired |
| NHBE  Fig. 4  Fig. 5 | In vitro (infected cells) | ~0.01 | 24h, 48h, 72h | GSE166766 | scRNA-seq | Paired |
| Case1  Fig. 1C, D, & E  Fig. 2 | Autopsy lung tissue | - | - | GSE150316 (GSM4546576, 4546577, 4546578, 4546579) | Bulk | Paired |
| Case8  Fig. 1C, D, & E  Fig. 2 | Autopsy lung tissue | - | - | GSE150316 (GSM4698544, 4698545, 4698546, 4698547, 4698548) | Bulk | Paired |
| Case9  Fig. 1C, D, & E  Fig. 2 | Autopsy lung tissue | - | - | GSE150316 (GSM4698549, 4698550, 4698551, 4698552, 4698553) | Bulk | Paired |
| Case11  Fig. 1C, D, & E  Fig. 2 | Autopsy lung tissue | - | - | GSE150316 (GSM4698526, 4698527, 4698528) | Bulk | Paired |
| CaseC  Fig. 1C, D, & E  Fig. 2 | Autopsy lung tissue | - | - | GSE150316 (GSM4698556) | Bulk | Paired |
| CaseD  Fig. 1C, D, & E  Fig. 2 | Autopsy lung tissue | - | - | GSE150316 (GSM4698557) | Bulk | Paired |
| CaseE  Fig. 1C, D, & E  Fig. 2 | Autopsy lung tissue | - | - | GSE150316 (GSM4698558) | Bulk | Paired |
| Longitudinal samples  Fig. 8A, B, C, D | Nasal | - | - | ENA: ERP132087, NCBI SRA: PRJEB47786 | ARTICv3-Bulk | Paired |
| ARTIC samples  Fig. 8E | Nasal | - | - | PRJNA707211 | ARTIC v1&v3-Bulk | Paired |
| Asymptomatic and Symptomatic samples  Fig. 7 | Nasal | - | - | PRJNA690577 | ARTICv3-Bulk | Paired |
