# Supplementary material for: Generation and Functional Analysis of Defective Viral Genomes during SARS-CoV-2 Infection
Source: mBio. 2023 Apr 19;14(3):e00250-23. doi: 10.1128/mbio.00250-23 (PMC10294654; doi:10.1128/mbio.00250-23)

ViReMa, Cellranger and Seurat Pipelines

Terry Zhou, Simone Spandau
13 July, 2022

Contents

Introduction **2**

DVG analysis from bulk RNA-Seq dataset **2**

Bowtie2 2

ViReMa 3

Subread 3

R Filtering Script 3

Making Plots 4

DVG analysis from scRNA-Seq dataset **6**

UMI-Tools 7

Bowtie2 7

ViReMa 7

R Filtering Script 7

Making Plots 12

Cellranger and Seurat **21**

Reference Genome **21**

SARS-CoV2 FASTA and GTF 21

Creating reference package for Cellranger 21

Gene Expression Matrix **22**

Cellranger count 22

Loading matrix into R and creating csv files 22

Celltype Identification 23

Celltype Percents 25

Differential Gene Expression 25

Fig 5 A: GO dotplots 27

Introduction

The purpose of this standard operating procedure is to outline the pipeline used for ViReMa. This document describes the steps needed to identify and analyze defective viral genomes (DVGs) from bulk RNA-Seq and single cell RNA-Seq (scRNA-Seq) data in SARS-CoV-2.

The Bowtie2 and ViReMa scripts were both run on the BlueHive Linux computing cluster supported by the Center for Integrated Research Computing at the University of Rochester.

We used version 2.2.9 of Bowtie2 to map our samples to the human genome. We used the GRCh38 (hg38) human reference genome. We also used UMI-Tools version b1 for our single cell RNA-Seq analysis.

We used version 0.21 of ViReMa to identify the DVG recombinant events and their corresponding counts. Version 0.21 of ViReMa uses version 0.12.9 of Bowtie and Python3 to map each sample to the reference viral genome. We used the SARS-CoV-2 reference genome with GenBank ID MT020881.1.

For the rest of our analysis, we used version 4.1.0 of R and version 1.4.1717 of RStudio. Our analysis used the following packages:

- Rsubread
- tidyverse
- ggplot2
- plotly
- openxlsx
- data.table

DVG analysis from bulk RNA-Seq dataset

The pipeline to identify DVGs from bulk RNA-Seq analysis was as follows:

1. Bowtie2
2. ViReMa
3. Subread
4. R filtering

Bowtie2

We used Bowtie2 to align our sample to the human reference genome (GRCh38 (hg38)). The GRCh38 (hg38) index was downloaded from the Bowtie2 website. The unmapped output sequence served as the viral sequence to be used for ViReMa.

The SLURM script used to run Bowtie2 alignment for single end reads is shown below:

```
module load bowtie2/2.2.9

bowtie2 -x /scratch/tzhou18/hg38index/GRCh38_noalt_as -U
/scratch/tzhou18/sample1.fastq.gz -p 8
--un-gz ./sample1.unmapped.fq.gz --al-gz ./sample1.mapped.fq.gz
```

For paired end samples, properly paired read files were specified using the -1 and -2 options instead of the -U option used for single end reads.

ViReMa

We used ViReMa to identify viral recombinant events.

```
module load bowtie/0.12.9
module load python3

python3 /scratch/tzhou18/ViReMa_0.15/ViReMa_0.21/ViReMa.py
/scratch/tzhou18/sars2_MT020881.fasta /scratch/tzhou18/sample1.unmapped.fq.gz
/scratch/tzhou18/sample1_recombinations.bam --MicroInDel_Length 5 --Aligner_Directory
/software/bowtie/0.12.9 -BED --Output_Dir /scratch/tzhou18/sample1
```

The Virus_Recombination_Results.bed file within the BED_Files folder and the recombinations.bam file were used for the downstream analysis.

Subread

Bioconductor R package Rsubread (v2.6.4) was used to align our RNA-seq data to the viral reference genome to identify the number of viral reads in each sample.

To import each sample into RStudio to run Subread, a tab-delimited file named study_design.txt was created to contain the file names and paths, as shown below:

```
fastq	sample	path
sample1.fastq.gz		sample1  /Users/terryzhou/
sample2.fastq.gz		sample2 /Users/terryzhou/
sample3.fastq.gz		sample3 /Users/terryzhou/
```

The following R script was used to run Rsubread in RStudio.

```
library(Rsubread)

targets <- read.table("study_design.txt",
                      row.names=NULL, header = T, as.is = T)

# Build Index from genome fasta ------------------------------
buildindex(basename="reference_name", reference="reference.fasta")
# make sure the reference.fasta has lines that are less than 1000 bases long

# Align reads ----------------------------------------
reads <- targets$fastq
align(index="reference_name", readfile1=reads, input_format="gzFASTQ",
      output_format="BAM", unique=TRUE, indels=5, nthreads=8)
```

The number of viral reads printed out in the R console, as well as the subread.BAM.summary file, in the Uniquely_mapped_reads row were used as the total counts of viral reads (Fig1B_virus).

R Filtering Script

The following R script was used to filter out recombinations that are not deletions (i.e. insertions, duplica-tions), deletions shorter than 100 nt, and those that had a break point before the 85 nt position. We also separated the identified DVGs into positive and negative sense and analyzed them separately.

Since viral load can affect DVG level, we used the junction frequency (J_{freq}) as a standardized value to quantify DVG level. We calculated J_{freq} by dividing the DVG count by the viral read count. The viral read count was identified via the previous Subread step.

The filtering script is shown below:

```
library(tidyverse)

# import ViReMa output BED file as csv with headers T1 <-
read.csv("2dpi_recombinations.csv")

#  filter out recombinations that are not deletions
T1_final <- filter(T1, Type == "Deletion")

#   calculate  deletion  lengths
T2 <- mutate(T1_final, Deletion_length = abs(Break_Point - Rejoin_Point))
# filter out deletions shorter than 100 nt T2_less <-
filter(T2, Deletion_length > 100)
# filter out deletions with break points before the 85 nt location T2_final <-
filter(T2_less, Break_Point > 85)
# negative sense DVGs have their break points labeled as Rejoin_Point T2_final <-
filter(T2_final, Rejoin_Point > 85)

# calculate ratio of DVGs at that position


# add sample ID name column
T3['ID'] = '2dpi'

#   calculate Jfreq

#  replace the 300000 with actual numerical viral counts
#  obtained in the Subread section

#  separate T3 into positive and negative sense
T3_neg <- subset(T3, strand == "-")
T3_pos <- subset(T3, strand == "+")

write.csv(T3, file = "T3_2dpi.csv", row.names = FALSE)
```

Making Plots

We used the following script to graph plots as shown in Fig. 2 and Fig. S1.

```
library(ggplot2)
# plot negative sense break and rejoin distribution neg.rejoin <-
ggplot(T3_neg, aes(width = 300)) +
   geom_col(aes(x = Rejoin_Point, y = Counts, fill = Deletion_length)) +
   coord_cartesian(ylim = c(0, 10), xlim = c(0, 30000)) + scale_x_continuous(breaks=seq(0,
   30000, 2000)) +
   labs(x = "Rejoin point", y = "Total reads",
           title = "Rejoin point usage distribution (negative sense)", fill = "Deletion
           Length") +
```

```
    theme_bw() +
    theme(legend.position = "right",
              axis.text = element_text(size = 8),
              axis.title = element_text(size = 16))
neg.break <- ggplot(T3_neg, aes(width = 300)) +
    geom_col(aes(x = Break_Point, y = Counts, fill = Deletion_length)) +
    coord_cartesian(ylim = c(0, 10), xlim = c(0, 30000)) + scale_x_continuous(breaks=seq(0,
    30000, 2000)) + labs(x = "Break point", y = "Total reads",

            title = "Break point usage distribution (negative sense)", fill = "Deletion
            Length") +
    theme_bw() +
    theme(legend.position  =  "right",
              axis.text = element_text(size = 8),
              axis.title = element_text(size = 16))

# DVG distribution -- Counts
neg.dist <- ggplot(T3_neg, aes(y = Break_Point, x = Rejoin_Point, size = Counts, color =
                                                                      Deletion_length),
                                    alpha = 0.5) +
    geom_point() +
    labs(y = "Break point (nt)", x = "Rejoin point (nt)", title= "All
            Deletions (negative sense)",
            color = "Deletion Length", size = "Counts") +
    geom_rug(aes(color = Deletion_length)) + theme_bw() +

    xlim(0, 30000)+ ylim(0, 30000)+
    theme(legend.position = "right", axis.text = element_text(size = 8), axis.title =
              element_text(size = 16))
```

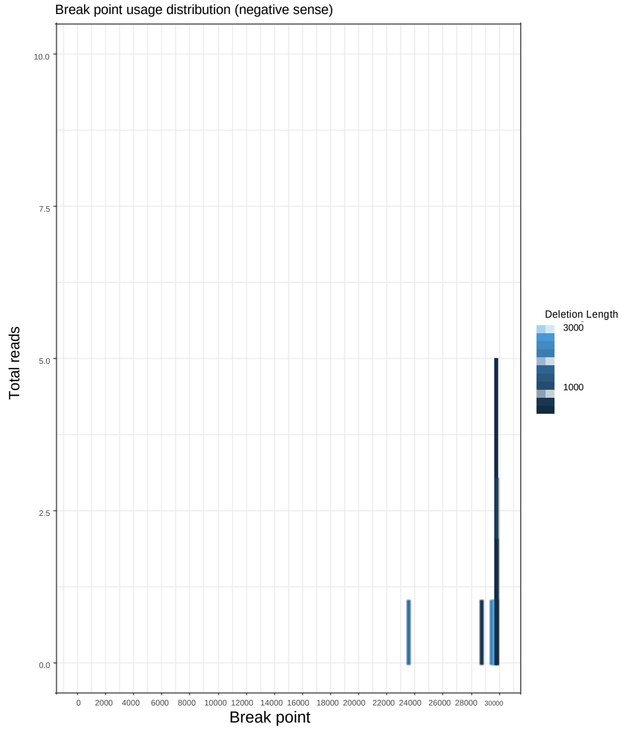

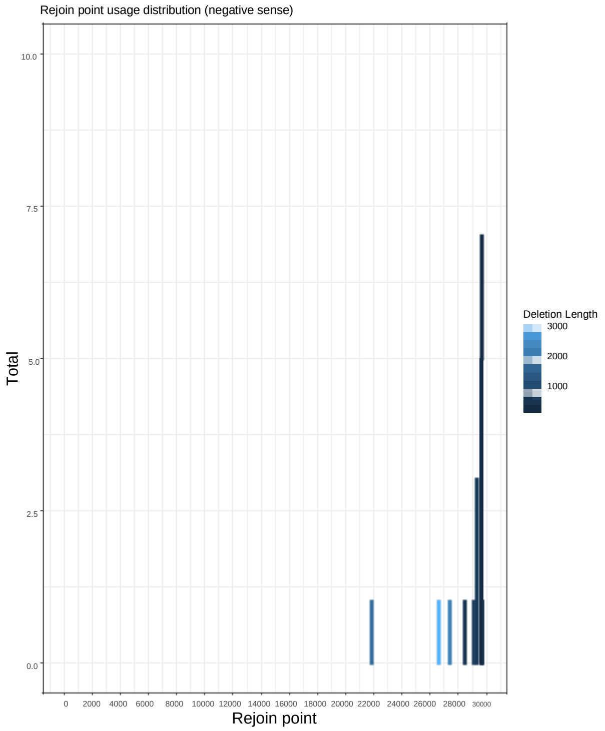

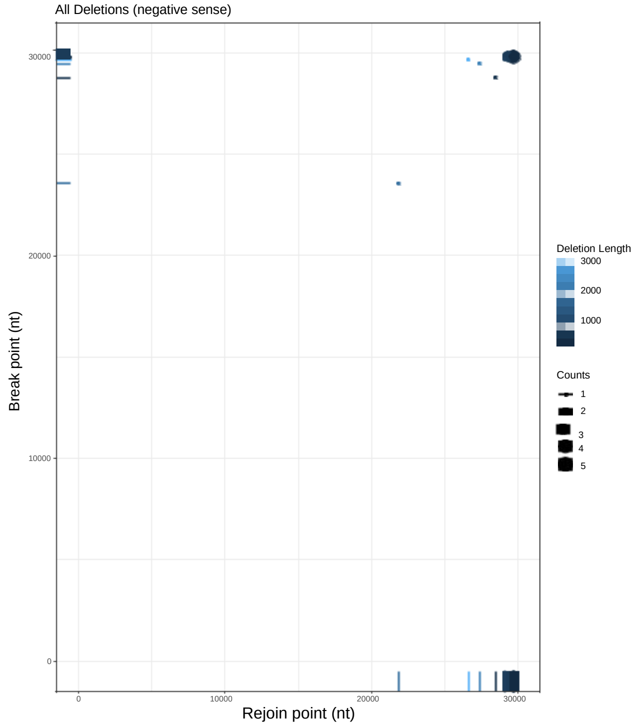

DVG analysis from scRNA-Seq dataset

The pipeline to identify DVGs from scRNA-Seq analysis was as follows:

1. UMI-Tools

2. Bowtie2
3. ViReMa
4. R filtering

UMI-Tools

UMI-Tools was used to associate the cell barcodes and UMIs for each read to the sequence. The cell barcodes and UMIs from the R1 file were combined with the corresponding read in the R2 file.

```
module load umi-tools

umi_tools extract --bc-pattern=CCCCCCCCCCCCCCCCNNNNNNNNNNNN
--extract-method=string
--stdin=1dpi_CoV2_HHT_S2_L001_R1_001.fastq.gz        --stdout=1dpi_out_R1.fastq.gz
--read2-in=1dpi_CoV2_HHT_S2_L001_R2_001.fastq.gz --read2-out=1dpi_out_R2.fastq.gz -L extract.log
```

Bowtie2

As with the bulk RNA-Seq analysis, we are used Bowtie2 to align our sample to the human genome. The unmapped output file was used for ViReMa analysis.

```
module load bowtie2/2.2.9

bowtie2 -x /scratch/tzhou18/hg38index/GRCh38_noalt_as -U
/scratch/tzhou18/1dpi_out_R2.fastq.gz -p 8
--un-gz ./1dpi.unmapped.fq.gz --al-gz ./1dpi.mapped.fq.gz
```

ViReMa

For scRNA-Seq analysis, ViReMa must be run twice in order to link the cell barcodes and UMIs to the identified DVGs. The first run was identical to running ViReMa for bulk RNA-Seq. The second run was as follows:

```
module load bowtie/0.12.9
module load python3

python3 /scratch/tzhou18/ViReMa_0.15/ViReMa_0.21/ViReMa.py
/scratch/tzhou18/sars2_MT020881.fasta 1dpi.unmapped.fq.gz
1dpi_recombinations.bam --MicroInDel_Length 5 --Aligner_Directory
/software/bowtie/0.12.9
-BED --Output_Dir /scratch/tzhou18//1dpi -ReadNamesEntry
```

The Virus_Recombination_Results.bed file within the BED_Files folder and the recombinations.bam file from the first run and the Virus_Recombination_Results.txt file from the second run were used for the following R filtering.

R Filtering Script

The first section of the R filtering script was identical to bulk RNA-seq section.

```
library(ggplot2)
library(tidyverse)
library(openxlsx)
library(data.table)

#  filter ViReMa output
#   this section is the same for both bulk and scRNA
#  import ViReMa output BED file as csv with headers T1 <-
read.csv("3dpi_recombinations.csv")

#  filter out recombinations that are not deletions
T1_final <- filter(T1, Type == "Deletion")

#  calculate  deletion  lengths
T2 <- mutate(T1_final, Deletion_length = abs(Break_Point - Rejoin_Point))
  #  filter out deletions shorter than 100 nt T2_less <-
filter(T2, Deletion_length > 100)
#  filter out deletions with break points before the 85 nt location T2_final <-
filter(T2_less, Break_Point > 85)
#  negative sense DVGs have their break points labeled as Rejoin_Point T2_final <-
filter(T2_final, Rejoin_Point > 85)

#  calculate ratio of DVGs at that position


# add sample ID name column
T3['ID'] = '3dpi'

#  separate T3 into positive and negative sense
T3_neg <- subset(T3, strand == "-", select = Break_Point:ID)
T3_pos <- subset(T3, strand == "+", select = Break_Point:ID)

# save T3 dataframe as csv file
write.csv(T3, file = "T3_3dpi.csv", row.names = FALSE)
```

This next section of the R filtering was unique to scRNA-seq analysis. The input file for this section was the Recombination_Results.txt file from the second run of ViReMa, including all of the read counts, positions, and cell barcodes/UMIs for each DVG.

The viral.reads dataframe, including the number of viral UMIs for each cell and lists the cells in the order, was used for downstream Seurat analysis. This dataframe was retrieved by outputting only the viral reads row from the Cell Ranger gene matrix into a .csv file.

The final dataframes of interest from the following R filtering script were the df1, df2, and df3 dataframes, which included the number of DVG UMIs per cell, the number of DVG UMIs per position and the number of DVG UMIs per combination of cell and position, respectively. These datasets were used for downstream analyses. In addition, the bcmatrix dataframe was added to the matrix in Seurat as a “DVG gene.”

The following script only showed the filtering process for the positive sense DVGs, however the same script was used for the negative sense DVGs.

```
recomb     <-    read.csv("3dpi_Virus_Recombination_Results.txt")
#  give column temporary header name
colnames(recomb) <- c("xx")
```

```
#  remove rows that are @NewLibrary and @EndOfLibrary ind <-
(startsWith(recomb$xx, "@"))
temp.dat <- recomb[!ind, ]
#  dat dataframe has one column with pos/count in odd numbered rows
#   and all of the barcodes in even rows
dat <- as.data.frame(temp.dat)

#  reformat dat dataframe into two columns
#  identify odd rows and making separate lists of odd and even rows row_odd <-
seq_len(nrow(dat)) %% 2
dat.odd <- dat[row_odd == 1, ]
dat.even <- dat[row_odd == 0, ]
#  join odd and even rows into two columns,
#  so each row is pos/count then all cell barcodes
dat1 <- cbind(dat.odd, dat.even)
dat1 <- as.data.frame(dat1)

#  filter based on DVG filtering from T3 above
#  creating new column that combines break/rejoin/counts
T3$PosCount <- paste0(T3$Break_Point, "_to_", T3$Rejoin_Point, "_#_", T3$Counts)
T3_neg$PosCount <- paste0(T3_neg$Break_Point, "_to_",
                                       T3_neg$Rejoin_Point, "_#_", T3_neg$Counts)
T3_pos$PosCount <- paste0(T3_pos$Break_Point, "_to_",
                                       T3_pos$Rejoin_Point, "_#_", T3_pos$Counts)
# filter all
dat1.1 <- recomb %>%
   filter(xx %in% T3$PosCount)
dat1.2 <- dat1 %>%
   filter(dat.odd %in% dat1.1$xx)
#   filtering only positive sense
dat1.1.pos <- recomb %>%
   filter(xx %in% T3_pos$PosCount)
dat1.2.pos <- dat1 %>%
   filter(dat.odd %in% dat1.1.pos$xx)

#   positive sense
#  create new df with each column being a different DVG (position and count)
# each row is a different cell
dat3.pos.umi <- dat1.2.pos %>%
   mutate(id = row_number()) %>%
   separate_rows(dat.even, sep = '_Fuzz=') %>%
   separate(dat.even, c('dat.even'), sep = '_Fuzz=') %>%
   group_by(id) %>%
   mutate(x = row_number()) %>%
   ungroup %>%
   pivot_wider(names_from = dat.odd, values_from=dat.even) %>% select(-
   c(id, x))
# move all cells with values to top
dat4.pos.umi <-
   data.table(dat3.pos.umi)[, lapply(.SD, function(x) x[order(is.na(x))])]
#  remove rows with all na from bottom
#  (but still includes some individual cells with na)
dat5.pos.umi <-
```

```
    dat4.pos.umi[rowSums(is.na(dat4.pos.umi)) != ncol(dat4.pos.umi), ]
# get rid of information in read name that isn't the cell barcode or UMI
dat6.pos.umi <-
    sapply(dat5.pos.umi, function(i) gsub(pattern = "[A-Z0-9:]+:[0-9]+_", replacement =
                                                    "", x = i) )
dat6.pos.umi <- as.data.frame(dat6.pos.umi)
#  get rid of any new lines, tabs, whitespaces
dat7.pos.umi <-
    sapply(dat6.pos.umi, function(i) gsub(pattern = "[\r\n\t\\S+]",
                                                    replacement = "", x = i) )
dat7.pos.umi <- as.data.frame(dat7.pos.umi)
# every cell barcode/ UMI in one column
dat7.pos.umi <- data.frame(newcol = c(t(dat7.pos.umi)))
dat7.pos.umi <- na.omit(dat7.pos.umi)
dat7.pos.umi$newcol <- as.character(dat7.pos.umi$newcol)
dat7.pos.umi <- subset(dat7.pos.umi, newcol != "NA")
dat7.pos.umi <- subset(dat7.pos.umi, newcol != "")
# new dataframe separating barcodes from UMIs
#  whether barcode or umi comes first will depend on the specific sample.
#   change as needed
dat8.pos.umi <-
    separate(dat7.pos.umi, newcol, into = c("barcode", "umi"), sep = "_")
dat8.pos.umi <- na.omit(dat8.pos.umi)
dat8.pos.umi$barcode <- as.character(dat8.pos.umi$barcode)
dat8.pos.umi$umi <- as.character(dat8.pos.umi$umi)
# for each cell, how many unique UMIs are there
dat9.pos.umi1 <- dat8.pos.umi %>%
    group_by(barcode) %>% distinct(umi)
    %>% summarise(unique.umi.count = n())


#   viral reads
#  import table with cell barcodes in order and viral UMI counts viral.reads <-
read.xlsx("3dpi_Barcodes.xlsx")
#  may have to modify these two lines based on how the imported table is formated
viral.reads <- separate(viral.reads, row_names,
                                into = c("barcode", "x"), sep = "-")
viral.reads <- viral.reads[,c(2,4)]
#  final viral reads dataframe should have column 1 be the cell barcode and
# column 2 be the viral UMI counts
colnames(viral.reads) <- c("barcode", "viral.read")

# DVG UMIs per cell
#  merge filtered DVGs with viral reads
#  not including cell barcodes that are in the filtered list,
#   but are not in the viral reads dataframe
df1.pos <- merge(dat9.pos.umi1,  viral.reads,  by = c("barcode"))
# calculate Jfreq
df1.pos$jfreq <- df1.pos$unique.umi.count / df1.pos$viral.read

#  DVG (UMI) per position
#  append positions to each cell barcode/UMI
dat6.pos.umi[] <- Map(paste, names(dat6.pos.umi), dat6.pos.umi, sep = ':')
```

```
 # make dataframe into just one column
xyz     <-      data.frame(x=unlist(dat6.pos.umi))
# separate out the positions
xyz <- separate(xyz, x, into = c("a", "b"), sep = ":")
xyz <- subset(xyz, b != "NA")
xyz <- subset(xyz, b != "\t")
# separate cell barcodes from UMIs
xyz <- separate(xyz, b, into = c("b", "c"), sep = "_")
#  create new column that counts the number of unique UMIs for each position xxyz <- xyz %>%

   group_by(a) %>% distinct(c) %>%
   summarise(unique.umi.count = n())

colnames(xxyz) <- c("pos", "unique.umi.count")
#  count the number of unique cells with DVGs at each position
y <- xyz %>%
   group_by(a) %>%
   distinct(b) %>%
   summarise(cells_per_position = n())
colnames(y) <-        c("pos",      "cells_per_position")
#  add number of cells for each position xxyz<-
merge(xxyz, y, by = c("pos")) xxyz <-
separate(xxyz, pos, sep = "_",
                                into = c("break_point", "to", "rejoin_point", "z", "count")) df2.pos <- xxyz[,
c(1, 3, 6, 7)]
df2.pos$break_point <- as.numeric(df2.pos$break_point)
df2.pos$rejoin_point <-           as.numeric(df2.pos$rejoin_point)
# calculate deletion lengths
df2.pos$deletion_length <- abs(df2.pos$break_point - df2.pos$rejoin_point)

#  DVG (UMI) per position + cell ----
#  merge position and cell columns xyzz <-
xyz %>%
   unite("a", a:b, sep= ":", remove = FALSE)
#  count number of unique UMIs with each position/cell combination xyzzz <- xyzz
%>%
   group_by(a) %>% distinct(c) %>%
   summarise(unique.umi.count = n())

xyzzz <- separate(xyzzz, a, sep = ":", into = c("pos", "barcode")) xyzzz <-
separate(xyzzz, pos, sep = "_",
                                 into = c("break_point", "to", "rejoin_point", "z", "count")) xyzzz <- xyzzz[, c(1,
3, 6, 7)]
#  merge with viral reads
#  not including cell barcodes that are in the filtered list,
#  but are not in the viral reads dataframe
df3.pos <- merge(xyzzz, viral.reads, by = c("barcode"))
# calculate Jfreq
df3.pos$jfreq <- df3.pos$unique.umi.count / df3.pos$viral.read
df3.pos$break_point <- as.numeric(df3.pos$break_point) df3.pos$rejoin_point
<- as.numeric(df3.pos$rejoin_point)
#  at this point you should have 3 dataframes:
# df1 is the DVG UMI count per cell
```

```
#  df2 is the DVG UMI count per break and rejoin positon
#  df3 is the DVG UMI count per combination of cell and position

#   barcodes in order for seurat ----
#  list of all cell  barcodes
barcodes <- viral.reads[, c(1)]
barcodes <- as.data.frame(barcodes)
# all cells with unique DVG UMI count per cell
bc <- rbind(df1.pos[, c(1, 2)])                        #add df1.neg[, c(1, 2)] to rbind if relevant
# get rid of duplicates
bc1 <- aggregate(unique.umi.count ~ barcode, bc, sum)
bclist <- bc1$barcode
counts <- bc1$unique.umi.count
#  create matrix of barcodes and counts with row1=barcodes row2=counts bcmatrix <-
rbind(bclist, counts)
#  make cell barcodes the column names
colnames(bcmatrix) <- bcmatrix[1,]
bcmatrix <- as.data.frame(bcmatrix)
#  get rid of row with cell barcodes bcmatrix
<- bcmatrix[-1,] rownames(bcmatrix) <-
c("counts")
#  list of all cell barcodes
bcs <- barcodes$barcodes
bcs <- as.factor(bcs)
#  identify DVG negative cells nondvg
<- setdiff(bcs, bclist)
#  identify DVG positive cells
posdvg <- bc1$barcode
# set DVG negative cells to DVG UMI count = 0
bcmatrix[nondvg] <- 0
#   order columns to match matrix needed for seurat
#  the order should match the column order of the viral.reads dataframe,
#   which should be in alphabetical order by cell barcode
bcmatrix <- bcmatrix[, order(colnames(bcmatrix))]
write.csv(bcmatrix, "3dpi_dvgmatrix.csv")
```

Making Plots

We used the following code to create exploratory data analysis plots of the positive sense DVGs, however the same code was used to visualize the negative sense DVGs.

```
library(plotly)
#plots for positive sense only
viral.percentile.95.pos <- quantile(df1.pos$viral.read,
                                               probs=seq(0, 1, 0.05))[20]
viral.hist.pos <- ggplot(df1.pos, mapping = aes(x = viral.read)) +
   geom_histogram(color = "black", fill = "pink") + theme_bw() +

   ggtitle("Histogram of Viral Load") +
   xlab("Unique UMIs per Cell")
plot.viral.load.pos <- ggplot(df1.pos, mapping = aes(x = "" , y = viral.read)) + geom_jitter(color =
   "hotpink", size=2, alpha=0.9) +
   theme_bw() +
```

```
  #ylim(0, 30) +
  ggtitle("Viral Load - UMI") +
  ylab("UMI Count per Cell Barcode") +
  theme(axis.title.x = element_blank(), axis.ticks.x = element_blank(), panel.grid.major.x =
          element_blank()) +
  geom_hline(yintercept     =   viral.percentile.95.pos,       color    =    "blue")

dvg.percentile.95.pos                    <- quantile(df1.pos$unique.umi.count,
                                          probs=seq(0, 1, 0.05))[20]
dvg.hist.pos<- ggplot(df1.pos, mapping = aes(x = unique.umi.count)) +
  geom_histogram(color = "black", fill = "pink") +
  theme_bw() +
  ggtitle("Histogram of DVG Count (UMI)") +
  xlab("Unique UMIs per Cell")
dvg.p.pos <- ggplot(df1.pos, mapping = aes(x = "" , y = unique.umi.count)) + geom_jitter(color =
  "hotpink", size=2, alpha=0.9) +
  theme_bw() +
  #ylim(0, 2) +
  ggtitle("DVG - UMI Per Cell Barcode") +
  ylab("UMI Count per Cell Barcode") +
  theme(axis.title.x = element_blank(), axis.ticks.x = element_blank(), panel.grid.major.x =
          element_blank()) +
  geom_hline(yintercept  =  dvg.percentile.95.pos,  color  =  "blue")

jfreq.percentile.95.pos <- quantile(df1.pos$jfreq, probs=seq(0, 1, 0.05))[20] dvg.jfreq.hist.pos <-
ggplot(df1.pos, mapping = aes(x = jfreq)) +
  geom_histogram(color = "black", fill = "pink") + theme_bw() +

  ggtitle("Histogram of Jfreq") +
  xlab("Jfreq")
dvg.ratio.p.pos <- ggplot(df1.pos, mapping = aes(x = "" , y = jfreq)) + geom_jitter(color =
  "hotpink", size=2, alpha=0.9) +
  theme_bw() +
  ggtitle("Jfreq = DVG UMI/ Viral UMI") +
  ylab("UMI Count per Cell Barcode") +
  theme(axis.title.x = element_blank(), axis.ticks.x = element_blank(), panel.grid.major.x =
          element_blank()) +
  geom_hline(yintercept  =  jfreq.percentile.95.pos,  color  =  "blue")

#  break/rejoin distribution graphs ----
#   break  point  distribution
break.pos <- ggplot(df2.pos) +
  geom_col(mapping = aes(x = break_point, y = unique.umi.count, fill =
                                  deletion_length, width = 300)) +
  coord_cartesian(ylim = c(0, 100), xlim = c(0, 30000)) +
  scale_x_continuous(breaks=seq(0, 30000, 2000)) + labs(x = "Break
  point", y = "Total reads",
        title = "Break point usage distribution (negative sense)", fill = "Deletion
        Length") +
  theme_bw() +
  theme(legend.position = "right", axis.text = element_text(size = 8), axis.title =
          element_text(size = 16))
# rejoin point distribution
```

```
rejoin.pos <- ggplot(df2.pos) +
    geom_col(mapping = aes(x = rejoin_point, fill = deletion_length, y =
                                              unique.umi.count, width = 300)) +
    coord_cartesian(ylim = c(0, 100), xlim = c(0, 30000)) +
    scale_x_continuous(breaks=seq(0, 30000, 2000)) + labs(x = "Rejoin
    point", y = "Total reads",
              title = "Rejoin point usage distribution (negative sense)", fill = "Deletion
              Length") +
    theme_bw() +
    theme(legend.position = "right", axis.text = element_text(size = 8), axis.title =
               element_text(size = 16))

dist.pos1 <- ggplot(df2.pos, mapping = aes(y = break_point, x = rejoin_point)) +
    geom_point(mapping = aes(size = unique.umi.count, color = deletion_length),
                     alpha = 0.5) +
    geom_rug(aes(color = deletion_length)) +
    theme_bw() +
    scale_colour_gradient(low = "red", high = "blue") + xlim(0,
    30000) + ylim(0, 30000) +
    labs(x = "Rejoin Point", y = "Break Point",
              title = "DVG Distribution (Positive Sense)",
              color = "Deletion Length", size = "DVG UMI Count") +
    theme(aspect.ratio = 1, legend.position = "right")
dist.pos2 <- ggplot(df2.pos, mapping = aes(y = break_point, x = rejoin_point)) +
    geom_point(mapping = aes(color = cells_per_position), alpha = 0.5) + geom_rug(aes(color =
    deletion_length)) +
    theme_bw() +
    scale_colour_gradient(low = "red", high = "blue") + xlim(0,
    30000) + ylim(0, 30000) +
    labs(x = "Rejoin Point", y = "Break Point", title = "DVG
              Distribution (Positive Sense)", color = "Cells Per
              Position") +
    theme(aspect.ratio = 1, legend.position = "right")
```

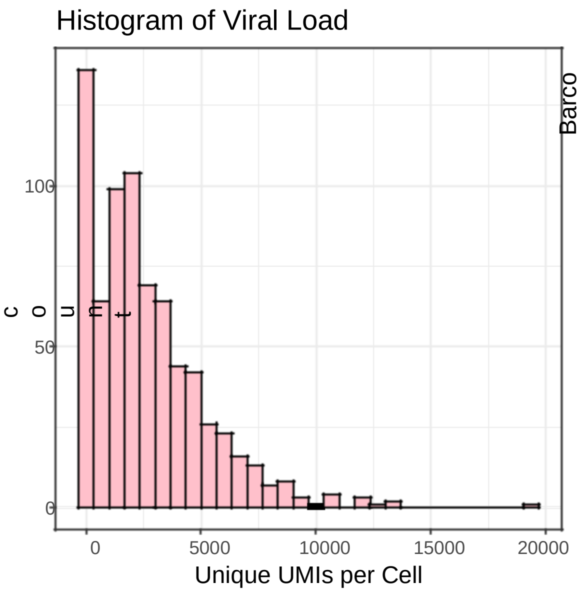

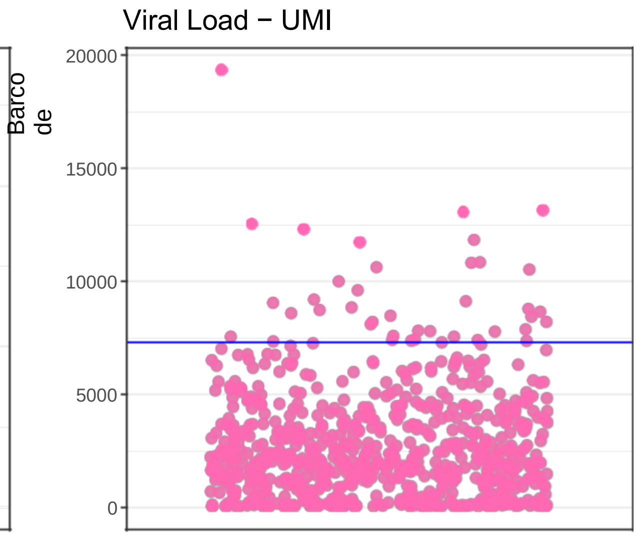

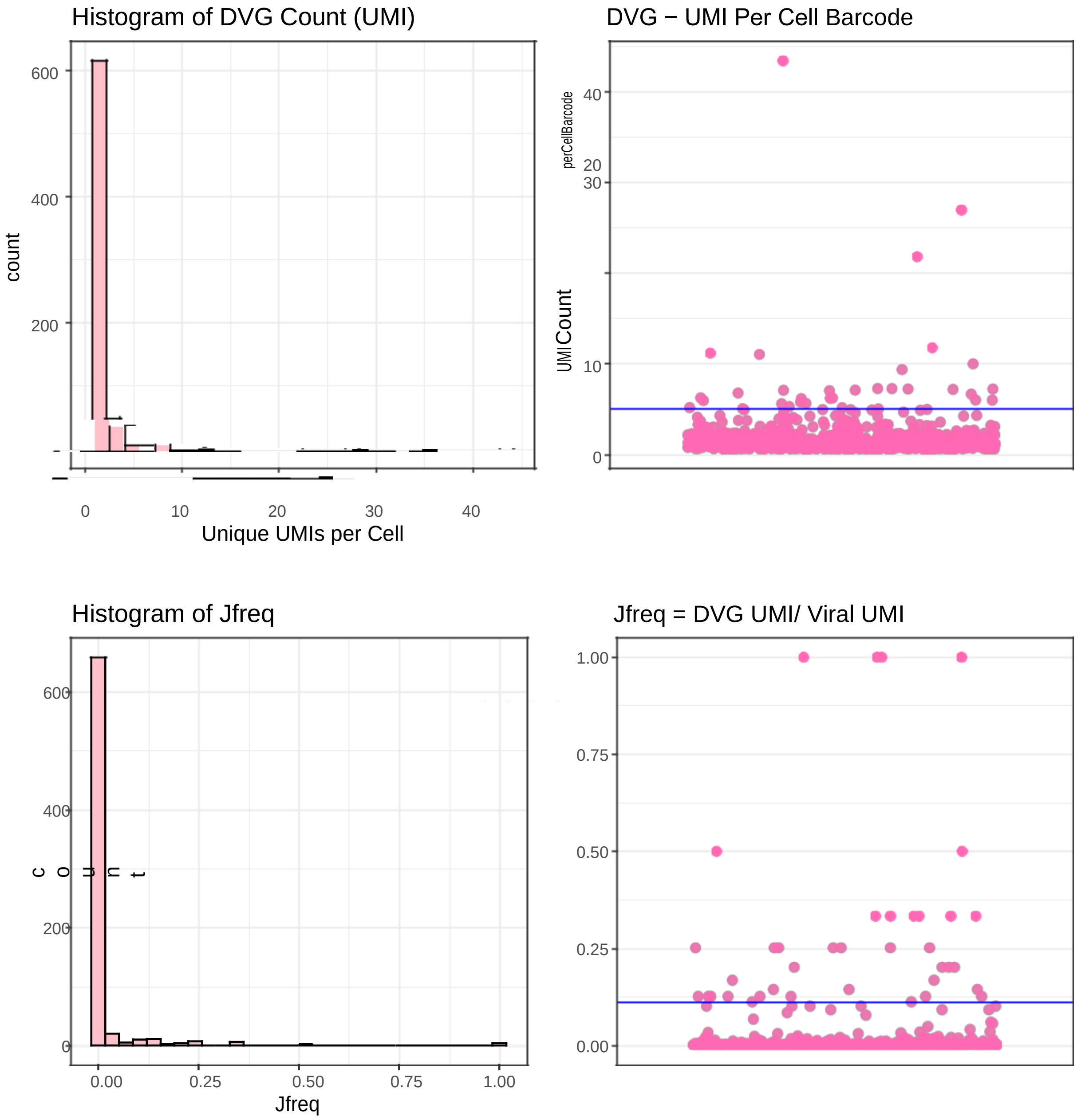

Histogram of DVG Count (UMI)
count
600
400
200
0
10
20
30
40
Unique UMIs per Cell
DVG − UMI Per Cell Barcode
UMICount
perCellBarcode
40
20
30
10
0
Histogram of Jfreq
600
400
200
0
0.00
0.25
0.50
0.75
1.00
Jfreq
Jfreq = DVG UMI/ Viral UMI
1.00
0.75
0.50
0.25
0.00

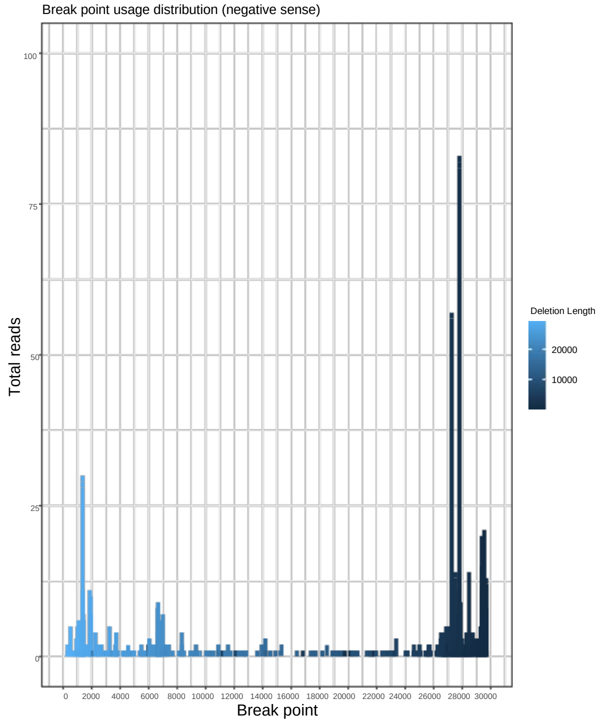

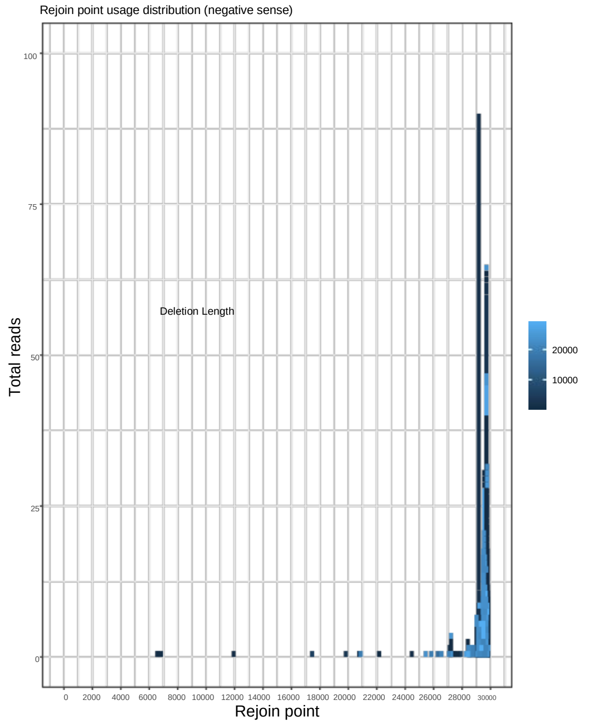

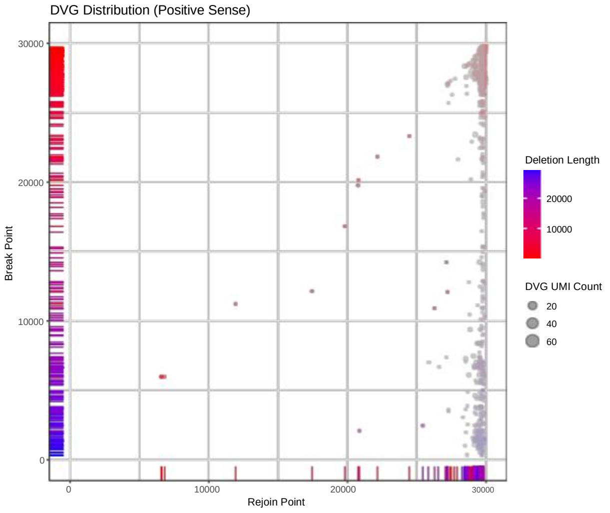

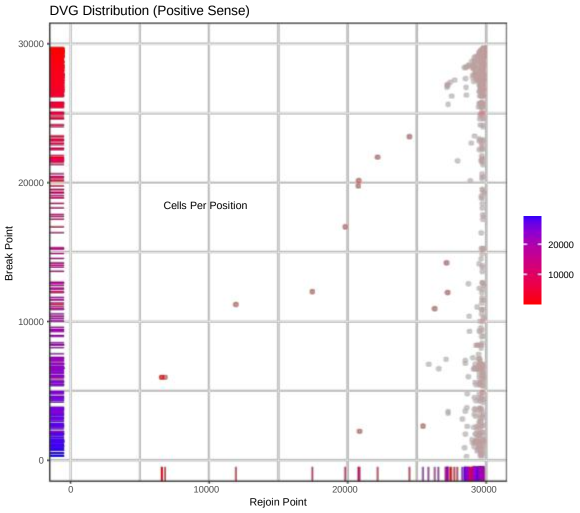

We used the following script to graph plots as shown in Fig. 4 and Fig. 5. The gene expression matrices were imported for each time point and the mock sample. The same script as Fig.4D was used to plot other time points and the same script as plots Fig.5A Fig.5B and Fig.5C was used to plot other time points and viral loads.

```
all <- rbind(genes.1dpi, genes.2dpi, genes.3dpi, mock)
all$virus <-
    ifelse(all$MT020881.1 >= 1 & all$MT020881.1 <= 10, "low",
                 ifelse(all$MT020881.1 > 10 &
                                    all$MT020881.1 <= 15000, "med", "high"))
all$jfreq <- (all$DVG / all$MT020881.1)
all$dvg.presence <- ifelse(all$DVG == 0, "neg", "pos")
all1 <- all[, -13]
all.infect <- subset(all1, MT020881.1 >= 1)
all2 <- all1[, c(1,11:16)]
all2.infect <- subset(all2, MT020881.1 >= 1)

Fig.4A <- ggplot(all2.infect, aes(x = day, y = log10(MT020881.1), color = day)) + geom_violin(width = 1,
    aes(fill = day), alpha = 0.2,) + geom_boxplot(width = 0.1, alpha = 0) +

    theme_classic() +
    scale_color_manual(values=c("dodgerblue4", "#E69F00",
                                    "forestgreen", "gray49")) +
    scale_fill_manual(values=c("dodgerblue4", "#E69F00",
                                    "forestgreen", "gray49")) +
    xlab("Sample") + ylab("log10(Viral UMI Count)") +
    ggtitle("Viral Load Per Sample") + theme(legend.position =
    "none")

Fig.4D  <-  ggplot(subset(all2.infect,  day  ==  "2dpi"),
          aes(x = dvg.presence, y = log10(MT020881.1), color = dvg.presence)) +
    geom_violin(width = 1, aes(fill = dvg.presence), alpha = 0.2,) + geom_boxplot(width = 0.1,
    alpha = 0) + theme_classic() +

    scale_fill_manual(values = c("dodgerblue", "brown3")) +
    scale_color_manual(labels = c("DVG+", "DVG-"),
                              values = c("dodgerblue", "brown3")) +
    xlab("Sample") + ylab("log10(Viral UMI Count)") +
    ggtitle("Viral Load by DVG Presence (2dpi)") +
    theme(legend.position = "none")
```

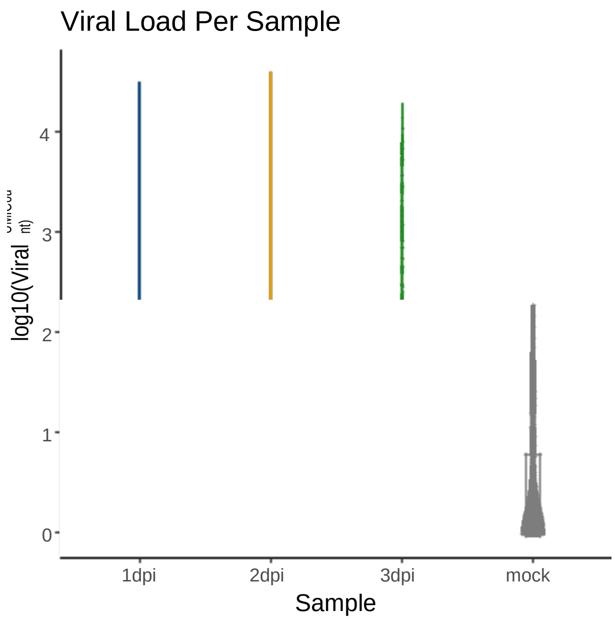

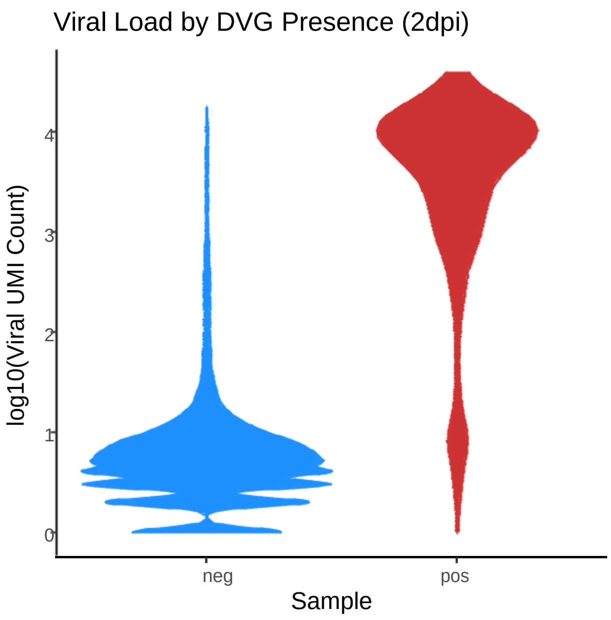


```
all2dpi <- subset(all, day == "3dpi")
all2dpi$virus <- factor(all2dpi$virus, levels = c("low", "med", "high"))
`Fig.5A Fig.5B` <- ggplot(data = all2dpi, mapping = aes(x = MT020881.1, y = MX1)) +
   geom_point(aes(colour = dvg.presence)) +
   facet_grid(vars(dvg.presence),                vars(virus))        +
   xlab("Viral Count") + ylab("MX1 Gene Expression") + ggtitle("MX1 vs. Virus") + theme_bw()


infected.2dpi <- subset(all.infect, day == "2dpi" & MT020881.1 >= 1) infected.2dpi <-
infected.2dpi[, c(1, 5, 8, 9, 10, 2, 7, 6, 4, 11:16)] infected.2dpi.long <-
gather(infected.2dpi, gene, exp, IFNB1:IL6,
                                                   factor_key = TRUE)
Fig.5C <- ggplot(data = subset(infected.2dpi.long, virus == "med")) +
   geom_violin(aes(x = gene, y = log10(exp + 1),
                              group = interaction(gene, dvg.presence),
                              color = dvg.presence, fill = dvg.presence),
                       scale = "width", position = position_dodge(width = 0.9), alpha = 0.2,
                       width = 0.8) +
   geom_boxplot(aes(x = gene, y = log10(exp + 1),
                              group = interaction(gene, dvg.presence),
                              color = dvg.presence),
                         position = position_dodge(width = 0.9),
                         width = 0.2, outlier.shape = NA, alpha = 0) +
   scale_color_manual(values = c("dodgerblue", "brown3"),
                                   labels = c("Negative", "Positive")) +
   scale_fill_manual(values = c("dodgerblue", "brown3"),
                                  labels  =  c("Negative",  "Positive"))  +
   theme_bw() + theme(aspect.ratio = 2/3, legend.position = "bottom") +
   ylim(c(0, 2.25)) +
   xlab("Gene") + ylab("Log10(Gene Expression + 1)") +
   labs(title = "Log Transformed Gene Expression by DVG Presence", subtitle =
         "2dpi, Moderate Viral Load",
         fill = "DVG Presence", color = "DVG Presence")
```

```
dvg.neg <- all2.infect %>%
   group_by(day, virus, dvg.presence) %>%
   summarize(dvg.neg = n()) %>%
   filter(virus == "low" & day != "mock") %>%
   group_by(day, virus) %>%
   mutate(total = sum(dvg.neg), neg.perc = (dvg.neg / total)*100) %>%
   filter(dvg.presence             == "neg")
Fig.5D <- ggplot(dvg.neg) +
   geom_col(aes(x = day, y = neg.perc, fill = day), color = "black") +
   scale_fill_manual(values = c("dodgerblue4", "goldenrod", "forestgreen")) +
   scale_color_manual(values = c("dodgerblue4", "goldenrod", "forestgreen")) +
   geom_text(aes(x = day, y = neg.perc,
                                                   label =paste0(round(neg.perc, 3), "%")), vjust = -0.75) +
   ylim(c(0, 105)) +
   theme_bw() + theme(legend.position = "none") +
   xlab("Day") + ylab("Percent of DVG- Cells") +
   ggtitle("DVG Negative Low Viral Infection Cells")
```

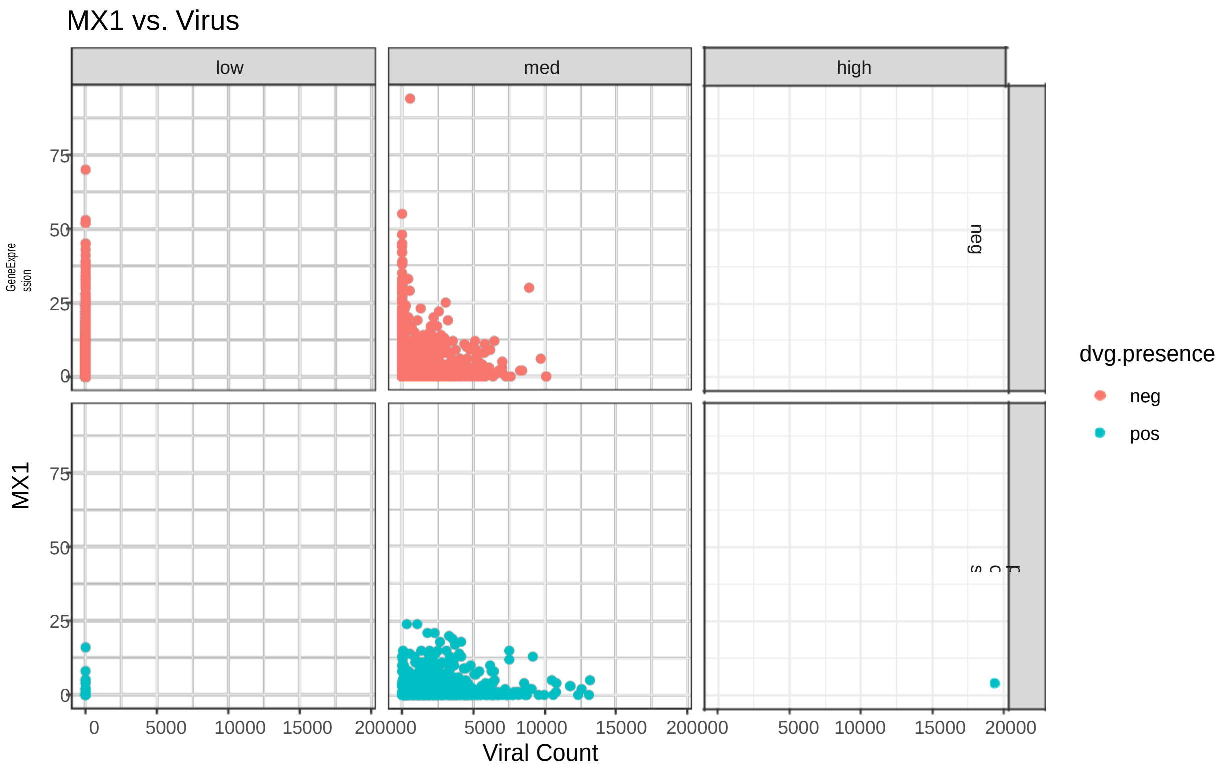

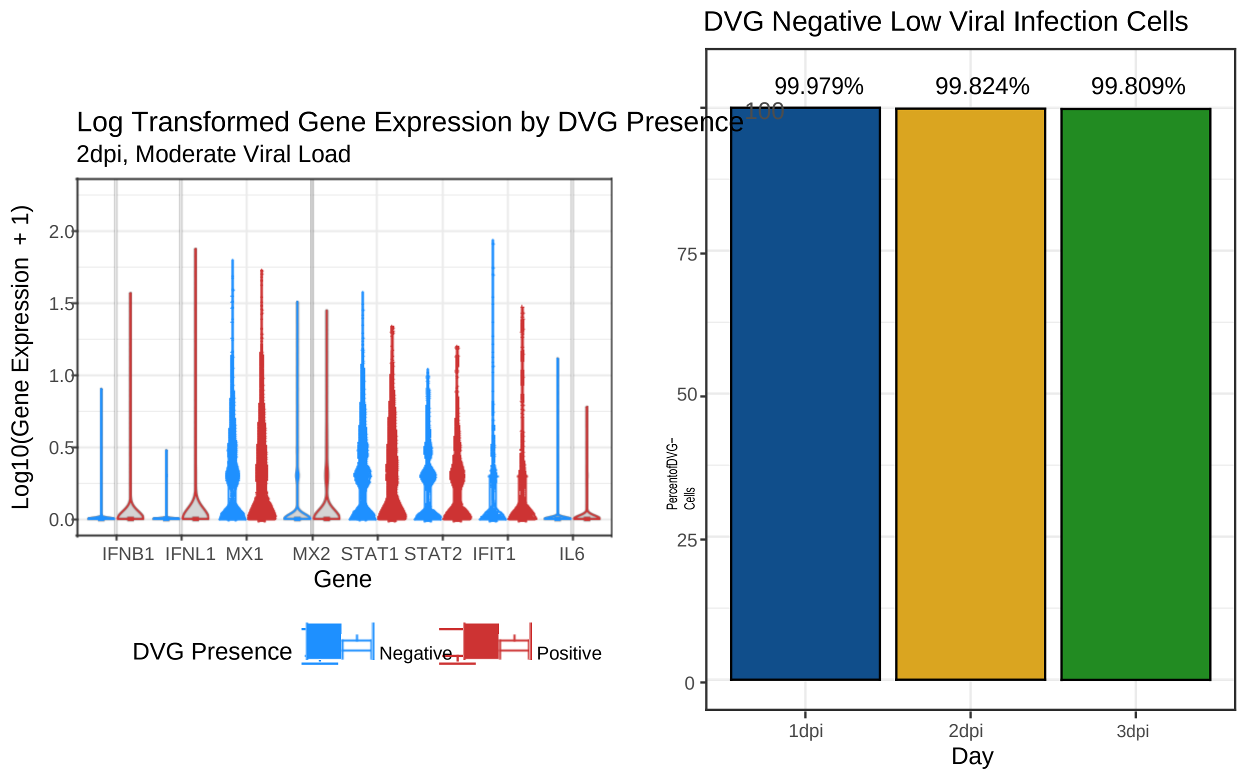

Log Transformed Gene Expression by DVG Presence
2dpi, Moderate Viral Load
Log10(Gene Expression + 1)
2.0
1.5
1.0
0.5
0.0
IFNB1 IFNL1 MX1 MX2 STAT1 STAT2 IFIT1 IL6
Gene
DVG Presence
Negative
Positive
DVG Negative Low Viral Infection Cells
99.979%
99.824%
99.809%
100
75
50
25
0
PercentofDVG- Cells
1dpi
2dpi
3dpi
Day

Cellranger and Seurat

The purpose of this section of the standard operating procedure is to outline the pipeline used for Cellranger and the Seurat R package for DVG counting and their impact on host responses from single cell RNA-seq.

We used version 6.1.2 of Cellranger to generate the gene expression matrices for our single cell RNA-Seq analysis.

This part of the analysis used the following R packages:

- Seurat
- Matrix
- sctransform
- Mast
- DESeq2
- tidyverse
- ggplot2
- dplyr
- data.table

Reference Genome

SARS-CoV2 FASTA and GTF

We dowloaded the SARS-CoV2 genome fasta file. For the MT020881.1 strain, it can be found in the following ncbi link. *https://www.ncbi.nlm.nih.gov/nuccore/MT020881.1?report=fasta*)
We made a custom GTF for the SARS-CoV2 genome such that it was labeled as a 'gene' in the human reference genome to which the covid genome was appended.

```
echo -e 'MT020881.1\tunknown\texon\t1\t(number of bases in genome, i.e. 29882)\t
.\t+\t.\tgene_id "MT020881.1"; transcript_id "MT020881.1"; gene_name "MT020881.1
"; gene_biotype "protein_coding";' > MT020881.1.gtf
```

The resulting gtf file looked like the following with the 'cat MT020881.1.gtf' command.

```
MT020881.1     unknown exon    1       29881     .       +       .       gene_id
 "MT020881.1"; transcript_id "MT020881.1"; gene_name "MT020881.1";
gene_biotype "protein_coding";
```

Creating reference package for Cellranger

We used the following shell script to run the mkref command in cellranger to create the reference package.

```
#!/bin/bash
#SBATCH -J cellrngr_ref
#SBATCH -e /scratch/sspandau/Yan_lab/cellrngr_ref_log.err
#SBATCH -o /scratch/sspandau/Yan_lab/cellrngr_ref_log.out
#SBATCH -t 24:00:00
#SBATCH -c 8
#SBATCH --partition=standard
#SBATCH --mem=24G

module load cellranger/6.1.2
```

```
cellranger mkref --genome=GRCh38_SARS-CoV2 --fasta=/path_to_concatenated_fasta/G
RCh38_SARS-CoV2.fa --genes=/path_to_concatenated_gtf/GRCh38_SARS_CoV2.gtf
```

The '–genome=' argument was for naming the resulting reference package. The '–fasta=' was for inputing the reference fasta, and the '–genes=' was for inputing the corresponding gtf file.

Gene Expression Matrix

Cellranger count

```
#!/bin/bash
#SBATCH -J cellrngrcount
#SBATCH -e /scratch/sspandau/Yan_lab/cellrngrcount.err
#SBATCH -o /scratch/sspandau/Yan_lab/cellrngrcount.out
#SBATCH -t 72:00:00
#SBATCH -c 8
#SBATCH --partition=standard
#SBATCH --mem=200G

module load cellranger/6.1.2

cellranger count --id=sample_name --transcriptome=/gpfs/fs2/scratch/sspandau/Yan
_lab/GRCh38_Covid19/ --fastqs=path/fastq --sample=id_fastqfilename
```

The '–id=' argument was for naming the output folder which contains the gene expression matrix.

The '–transcriptome=' argument was for inputing the path to the reference genome folder that was previously generated. The '–fastqs=' argument was for input the path(s) to the R1 and R2 fastqs of the sample. The '–sample=' argument was for input the sample id, which was the first few characters at the beginning of the R1 and R2 fastq file names.

Loading matrix into R and creating csv files

```
library(Seurat)
library(Matrix)
library(tidyverse)
library(dplyr)
library(data.table)
#read in matrix from cellranger
expression_matrix<- ReadMtx(
  mtx = "pathway/cellranger/outs/filtered_feature_bc_matrix/matrix.mtx.gz",featu
res = "pathway/cellranger/outs/filtered_feature_bc_matrix/features.tsv.gz",
  cells = "pathway/cellranger/outs/filtered_feature_bc_matrix/barcodes.tsv.gz",
)

#convert to data frame
#makes barcodes rows, easier to align dvg matrix
Expression_table <- as.data.frame(t(expression_matrix))
rm("expression_matrix")

#adding dvg matrix from R filter after scRNAseq
ViReMa DVG_UMI<- read.csv("pathway/dvgmatrix.csv")
```

```r
#makes barcodes rows, check to see if dvg matrix is already in this format
befor e using this code
DVG_UMI<-as.data.frame(t(DVG_UMI))

#create column with barcode
Expression_table<-tibble::rownames_to_column(Expression_table, "barcode")

#removes -1 that Seurat added to end of barcodes when matrix loaded into
R Expression_table$barcode <-
     sapply(Expression_table$barcode,function(i) gsub(pattern ="-
                                    1", replacement = "", x = i) )
#merges DVG matrix with Gene expression matrix based on barcode
Expression_DVG<-merge(Expression_table, DVG_Umi, by = "barcode")
#re-adds barcodes as row names
rownames(Expression_DVG)<-Expression_DVG$barcode
#removes barcode columns
Expression_DVG$barcode<- NULL

rm("DVG_UMI")
rm("Expression_table")
Expression_DVG<-as.matrix(Expression_DVG)
#naming DVG row as DVG, the row number below
#may be different depending on the number of features
#can check with this code : rownames(Expression_DVG)
rownames(Expression_DVG)[60667]<-"DVG"

#Target genes
#makes genes the rows
Expression_DVG<-as.data.frame(t(Expression_DVG))
#creates vector with target genes
data_keep_rows<-c("MX1", "MX2", "IL6", "IFIT1", "STAT1", "STAT2", "IFNB1", "IFNL
1", "TNF", "MT020881.1", "DVG")
#creates subset with target genes
Gene_subset<-Expression_DVG[rownames(Expression_DVG) %in% data_keep_rows, ]


#make barcodes the rows
Gene_subest<-as.data.frame(t(Gene_subset))
#add barcodes as column for excel
Gene_subset<-tibble::rownames_to_column(Gene_subset,
"row_names") #export to csv
write.csv(Gene_subset, "pathway/Gene_subset_name", row.names = TRUE)
```

Celltype Identification

```r
library(sctransform)
#Cell Type
Expression_DVG<-as.data.frame(Expression_DVG)
# to check if data frame was in right
format #rownames(Expression_DVG)
#colnames(Expression_DVG)
```

```
#load into seurat as a seurat data object
# min.cells filters out features that don't have counts in however many cells
#min.features filters out cells that don't have a certain number of features
det ected
seurat_Object<-CreateSeuratObject(counts = Expression_DVG, min.cells = 3, min.fe
atures = 200)

#normalize and scale data
seurat_Object <- SCTransform(seurat_Object)

#perform PCA
seurat_Object <- RunPCA(seurat_Object, features = VariableFeatures(object =
seurat_Object))

#Cluster the cells based on PCA and variable features
seurat_Object <- FindNeighbors(seurat_Object, dims = 1:10)
#change resolution based on how many cells were in scRNA data set (i.e. for
 3000 cells, resolution should be between 0.5 and 1.2, the higher the
 resolution, the more clusters)
seurat_Object<- FindClusters(seurat_Object, resolution = 0.2)

#print out gene markers for clusters
all_markers <-FindAllMarkers(seurat_Object, pval.type = "all", direction = "all"
)
markers <- as.data.frame(all_markers %>% group_by(cluster) %>% top_n(n = 10, wt
= avg_log2FC))
top10_markers

#renaming clusters based on cell types
#identify celltypes of each cluster based on top markers in the clusters
#if data set was already published, look in paper for which marker genes were
us ed for prior celltype identification
#can use online CellMarker data base (https://www.researchgate.net/deref/http%3A
%2F%2Fbio-bigdata.hrbmu.edu.cn%2FCellMarker%2F) or PanglaoDB (https://panglaodb.
se/) to look up marker genes and their associated celltypes

#create vector with new ident names (celltypes) in order of cluster number
 (i.e. first cluster is first celltype in vector)
new.cluster.ids.0.2res <- c("SLC16A7+", "Secretory", "Ciliated", "SLC16A7+")
#new.cluster.ids.0.8res <- c("SLC16A7+", "Ciliated", "SLC16A7+", "SLC16A7+",
"SL C16A7+","Secretory", "Unknown", "FOXN4+")

#rename idents (clusters)
names(new.cluster.ids.0.2res) <- levels(seurat_Object)
seurat_Object<-RenameIdents(seurat_Object, new.cluster.ids.0.2res)

#adding cell type to data frame
Celltype<-seurat_Object@assays$RNA@counts
Celltype<-as.data.frame(Celltype)
cell<-data.frame(seurat_Object@active.ident)
cell<-t(cell)
```

```
colnames(cell)=colnames(Celltype)
Celltype<-rbind(Celltype, cell)
rownames(Celltype)

# Creating celltype data frame
#If want matrix to just have covid counts, dvg counts, and cell type
#use following line, numbers may vary based on how many features are present
# Celltype<-Celltype[17626:17627,]
Celltype<-as.data.frame(Celltype)
rownames(Celltype) flipped<-
t(Celltype)
#convert new flipped expression table back to data
frame Celltype<-as.data.frame(flipped)
write.csv(Celltype, "Celltype_PHLE.csv", row.names = TRUE)
```

Celltype Percents

```
#read in csv created at the end of the celltype identification section
containin g celltypes, covid counts, and dvg counts for each cellbarcode
celltype_data<-read.csv("pathway/celltype.csv")
#to calculate number of cells in the
sample num_cells<-nrow(celltype_data)

#for celltype 1
# to calculate celltype percent
num_celltype1<-nrow(celltype_data[celltype_data$seurat_Object.active.ident == "n
ame_of_celltype1"])
percent_celltype1<-100*(num_celltype1/num_cells)
celltype1_subset<-subset(celltype_data, seurat_Object.active.ident == "name_of_c
elltype1")
#to calculate number of uninfected cells for that cell type
num_celltype1_uninfected<-nrow(celltype1_subset[celltype1_subset$MT020881.1 == 0
,])

celltype1_subset_infected<-subset(celltype1_subset, MT020881.1 >
0) #to calculate DVG+ cells
num_DVGpos<-nrow(celltype1_subset_infected[celltype1_subset_infected$DVG > 0,])
#to calculate DVG- cells
num_DVGneg<-nrow(celltype1_subset_infected[celltype1_subset_infected$DVG == 0,])

#Repeat for however many celltypes were in the data and for however many
 samples are being compared
#Add resulting statistics to csv
```

Differential Gene Expression

```
library(Mast)
library(DESeq2)
#subset Seurat object to only contain infected cells
#the infection threshold used below was at least one covid
count Q1<-subset(x = seurat_Object, subset = MT020881.1 > 0)
infectioncounts<- Q1@assays$RNA@counts
```

```
infectioncounts<-as.data.frame(infectioncounts)


#load in dvg status data frame
#the data frame had first column as barcodes (sometimes called row_names), and t
he second column had the DVG status for that cell (i.e. Y for DVG+, N for DVG-,
No_inf for uninfected cells)
dvg_status<-read.csv("dvg_status_PHLE.csv")
colnames(dvg_status)

#align gene matrix barcodes with dvg barcodes
infectioncounts<-as.data.frame(t(infectioncounts))
#name barcode column the same as it was named in the DVG status csv
infectioncounts<-tibble::rownames_to_column(infectioncounts, "row_names or barco
de")
#merge used to filter cells in expression matrix that were not present in dvg
st atus file
infectioncounts<-merge(infectioncounts, dvg_status, by = "row_names or barcode")
# makes barcodes the rownames instead of a column
rownames(infectioncounts)<-infectioncounts$row_names
#rownames(infectioncounts)

#merge used to remove cells in dvg status file that were not present in
 filtered expression matrix
dvg_status<-merge(dvg_status, infectioncounts, by = "row_names")
#returns dvg status data frame with just the barcodes and dvg
status dvg_status<-dvg_status[,c(1,2)]
#fix colnames after adjusting number of cells to matrix cells
colnames(dvg_status)<-c("row_names", "dvg_status")
#colnames(dvg_status)
rownames(dvg_status)<-dvg_status$row_names
ncol(infectioncounts)
#remove row_names and dvg status from expression matrix so that expression
matri x only has numerical data
infectioncounts<-infectioncounts[,-c(1,
29575)] colnames(infectioncounts)
# recreate seurat object with infectioncounts
#made features the rows and cell barcodes the columns such
that #it was in the correct format for seurat object
infectioncounts<-t(infectioncounts)
seurat_infected<- CreateSeuratObject(counts = infectioncounts)
#add dvg status as meta data
seurat_infected<-AddMetaData(seurat_infected, dvg_status$dvg_status, col.name =
'dvg_status')
#reset idents to be the dvg status meta data
seurat_infected<-SetIdent(seurat_infected, value = seurat_infected@meta.data$dvg
_status)

#use the following code to check if dvg status was correctly added as meta
data to seurat object
  #VlnPlot(seurat_infected, features = "MTND1P23", split.by = 'dvg_status', split.
```

```
plot = TRUE)

#standardization and normalization
seurat_infected<-SCTransform(seurat_infected)
#Find marker genes
list_mast<-FindMarkers(seurat_infected, ident.1 = "Y", ident.2 = "N", test.use =
 "Mast")
#Mast was DGE method for 0-inflated expression matrix
#DESeq2 and wilcox are other DGE methods that were used
list_mast<-as.data.frame(list_mast)
list_mast<-tibble::rownames_to_column(list_mast, "row_names")

#to calculate average expression for the two idents (DVG+ and DVG- cells)
#log normalize data
seurat_infected<-NormalizeData(object = seurat_infected)
#calculate average expression for the genes found in the DGE list above
avg_E <-AverageExpression(seurat_infected, features = list_mast$row_names)
avg_E<-as.data.frame(avg_E)
avg_E<-tibble::rownames_to_column(avg_E, "row_names")
#adds average expression to DGE list data frame such that resulting csv
contains p-vals, adjusted p-vals for differentially expressed genes, the the
percentage for which each genes were present in the two idents, and the average
expression for each gene in the two idents
list_mast_avgE<-merge(list_mast, avg_E, by = "row_names")
write.csv(list_mast_avgE, 'DGE_list_mast.csv')
```

Fig 5 A: GO dotplots

```
#for merging the 3 DGE lists from the three methods, Mast, DESeq2, and Wilcoxon
rank sum test
#Load in DGE lists
mast<-read.csv("pathway\DGE_list_mast.csv")
deseq2<-read.csv("pathway\DGE_list_DESeq2.csv")
wilcox<-read.csv("pathway\DGE_list_wilcox.csv")
#create merged data sets between each possible pairing of the
lists wilcox_mast<-merge(mast, wilcox, by = "row_names")
wilcox_deseq2<-merge(wilcox, deseq2, by = "row_names")
mast_deseq2<-merge(mast, deseq2, by = "row_names")
#bind newly merged data together
all<-rbind(wilcox_mast, wilcox_deseq2)
all<-rbind(all, mast_deseq2)
#remove any duplicated rows (i.e. genes found in all three)
all_noduplicate<-all[!duplicated(all$row_names), ]
#remove duplicated columns
all_noduplicate<-all_noduplicate[,-c(2:13)]
write.csv(all_noduplicate, "New_DGE_list_commongenes.csv")
```

After submitting DGE list to DAVID functional annotation tool and selecting the top pathways found in each cluster of the DAVID results, we used the following script to generate the GO dotplots.

```
library(ggplot2)
# load in top pathways from DAVID cluster results
```

```r
downreg<-read.csv("pathway/David_cluster_topdownreg_pathways.csv")
#add the pathway names as a factor with the levels being the generatio for
 those pathways
downreg$Description<-factor(downreg$Description, levels=downreg[order(downreg$Ge
neratio,decreasing=F),]$Description)
downreg<-as.data.frame(downreg)
upreg<-read.csv("pathway/David_cluster_upreg_pathways.csv")
upreg$Description<-factor(upreg$Description, levels=upreg[order(upreg$Generatio,
decreasing=F),]$Description)
upreg<-as.data.frame(upreg)

#plot the GO pathway enrichment
ggplot(downreg, #can replace the numbers to the row number of pathway of your
in terest
       aes(x = Generatio, y = Description)) +
  geom_point(aes(size = Genes.per.GO.category, color = Fold.Enrichment)) +
  theme_bw(base_size = 14) + theme(axis.text.y = element_text(size = 5))+
  scale_colour_gradient(limits=c(2, 100), low="coral1", high = "darkred") +
  ylab(NULL) +
  ggtitle("GO pathway enrichment Down Regulated")+theme(plot.title = element_tex
t(size = 15))

ggplot(upreg,
       aes(x = Generatio, y = Description)) +
  geom_point(aes(size = Genes.per.GO.category, color = Fold.Enrichment)) +
  theme_bw(base_size = 14) + theme(axis.text.y = element_text(size = 5))+
  scale_colour_gradient(limits=c(1, 20), low="coral1", high = "darkred") +
  ylab(NULL) +
  ggtitle("GO pathway enrichment Up Regulated")+theme(plot.title = element_text(
size = 15))
```

2dpi GO dotplots

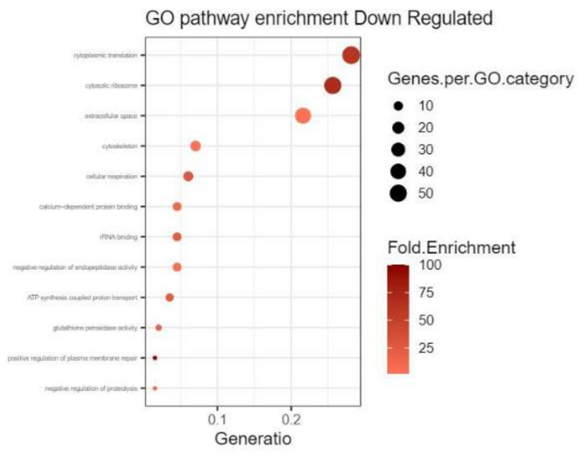

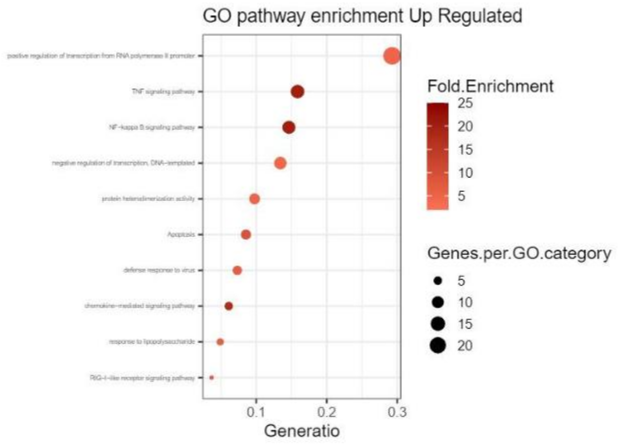

Supplement: TEXT S1 [file mbio.00250-23-s0010.pdf]
