# Supplementary figures and images for: Generation and Functional Analysis of Defective Viral Genomes during SARS-CoV-2 Infection
Source: mBio. 2023 Apr 19;14(3):e00250-23. doi: 10.1128/mbio.00250-23 (PMC10294654; doi:10.1128/mbio.00250-23)

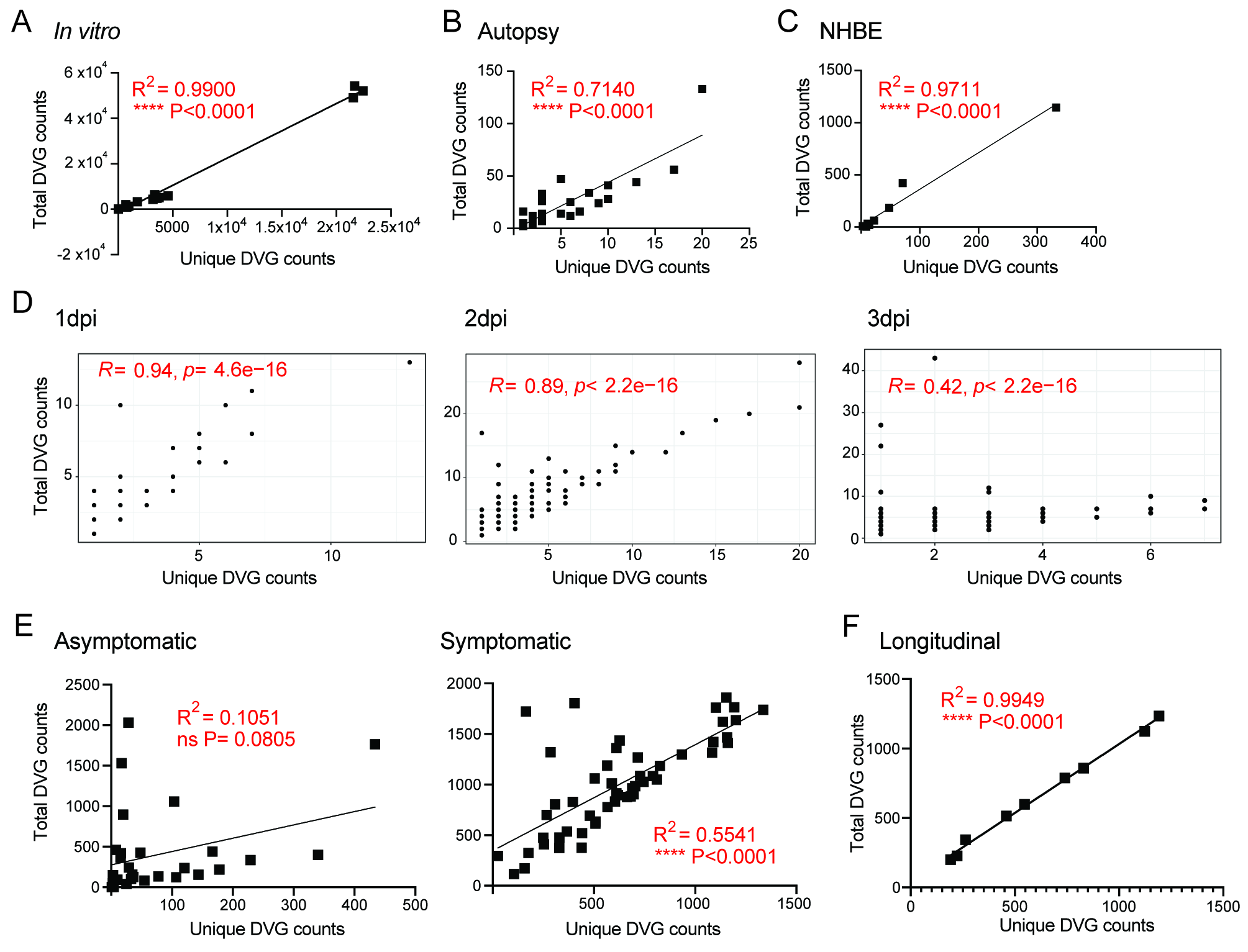

Supplement: FIG S1 [file mbio.00250-23-s0001.tif]

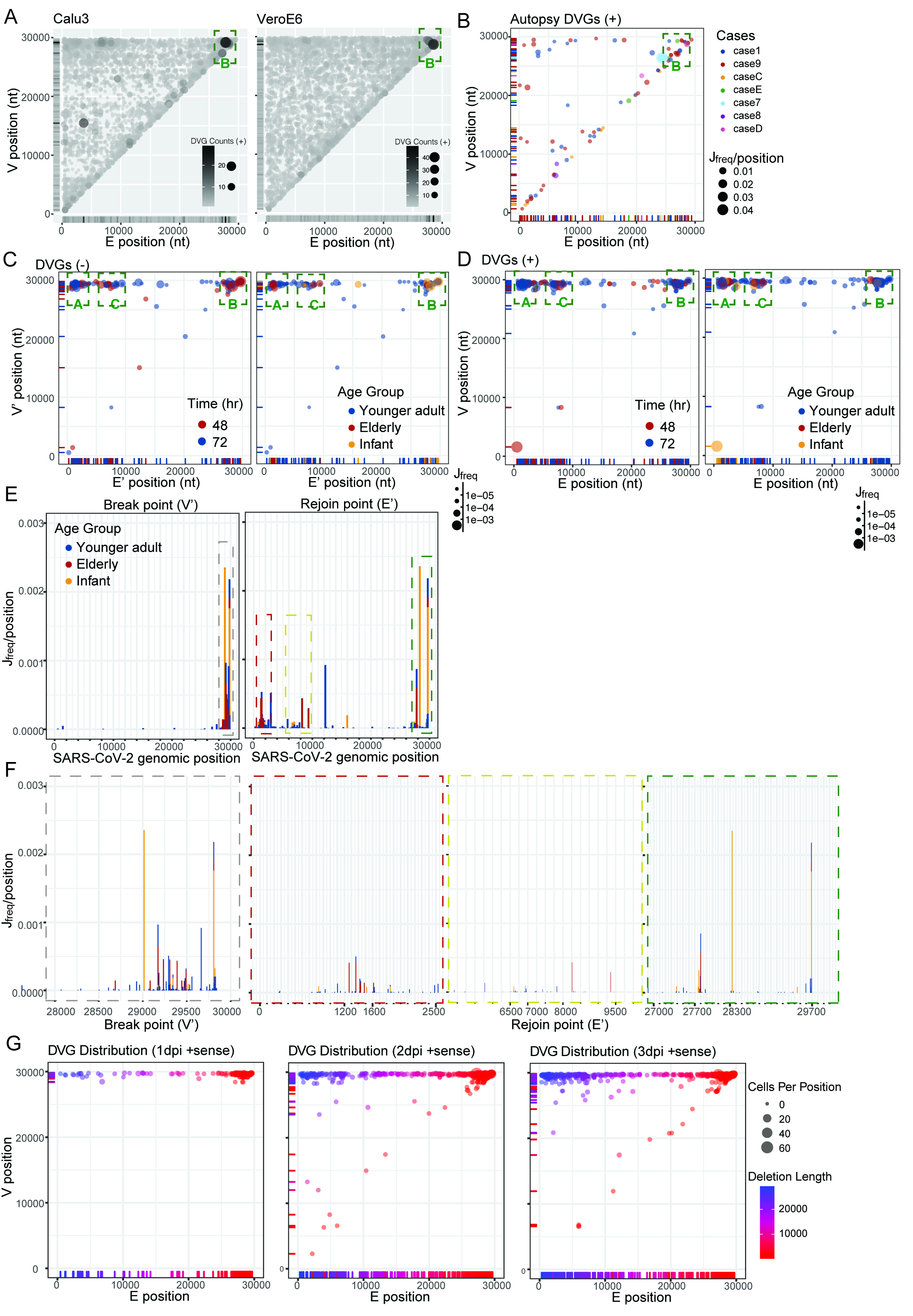

Supplement: FIG S2 [file mbio.00250-23-s0002.tif]

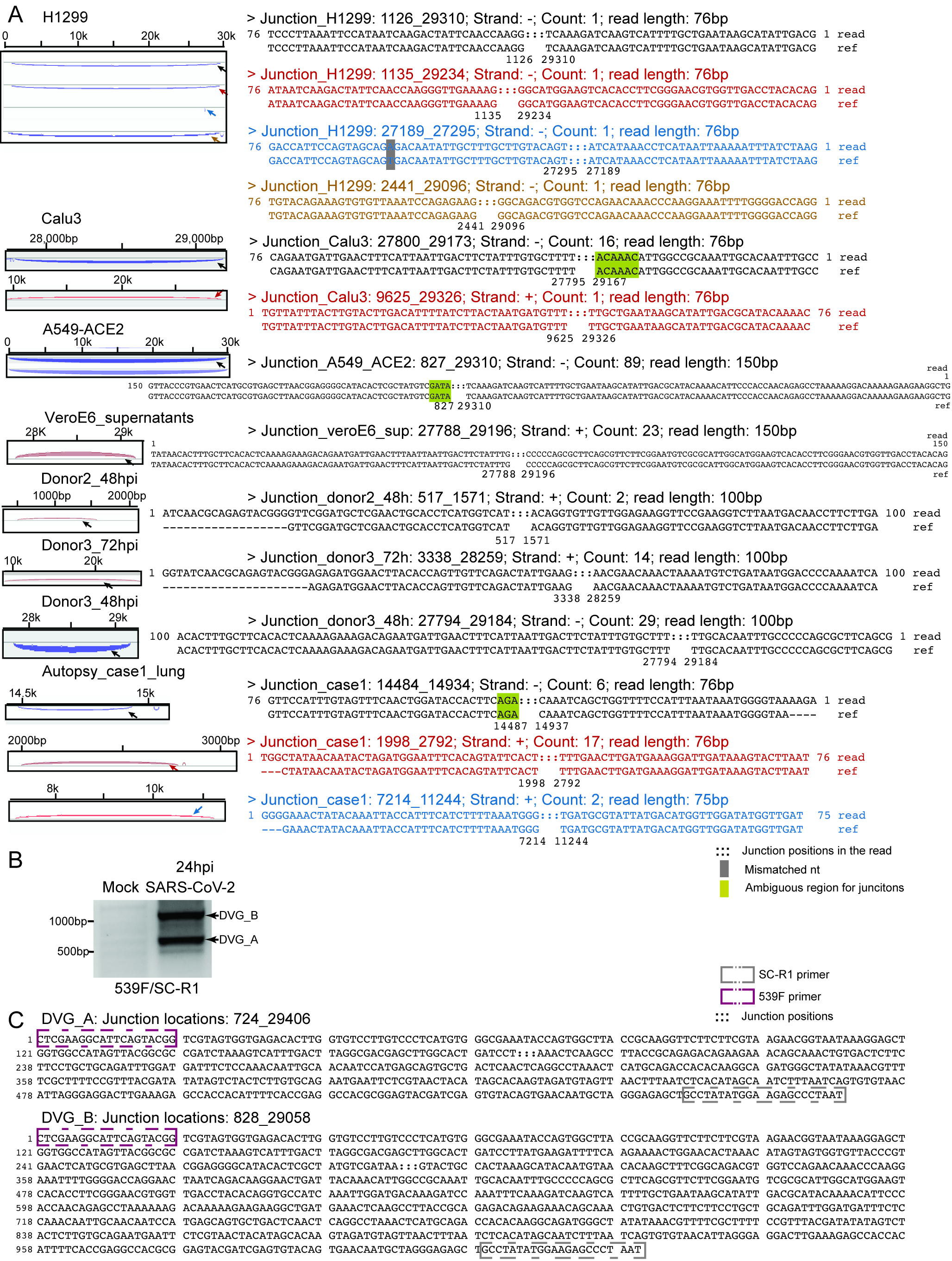

Supplement: FIG S3 [file mbio.00250-23-s0003.tif]

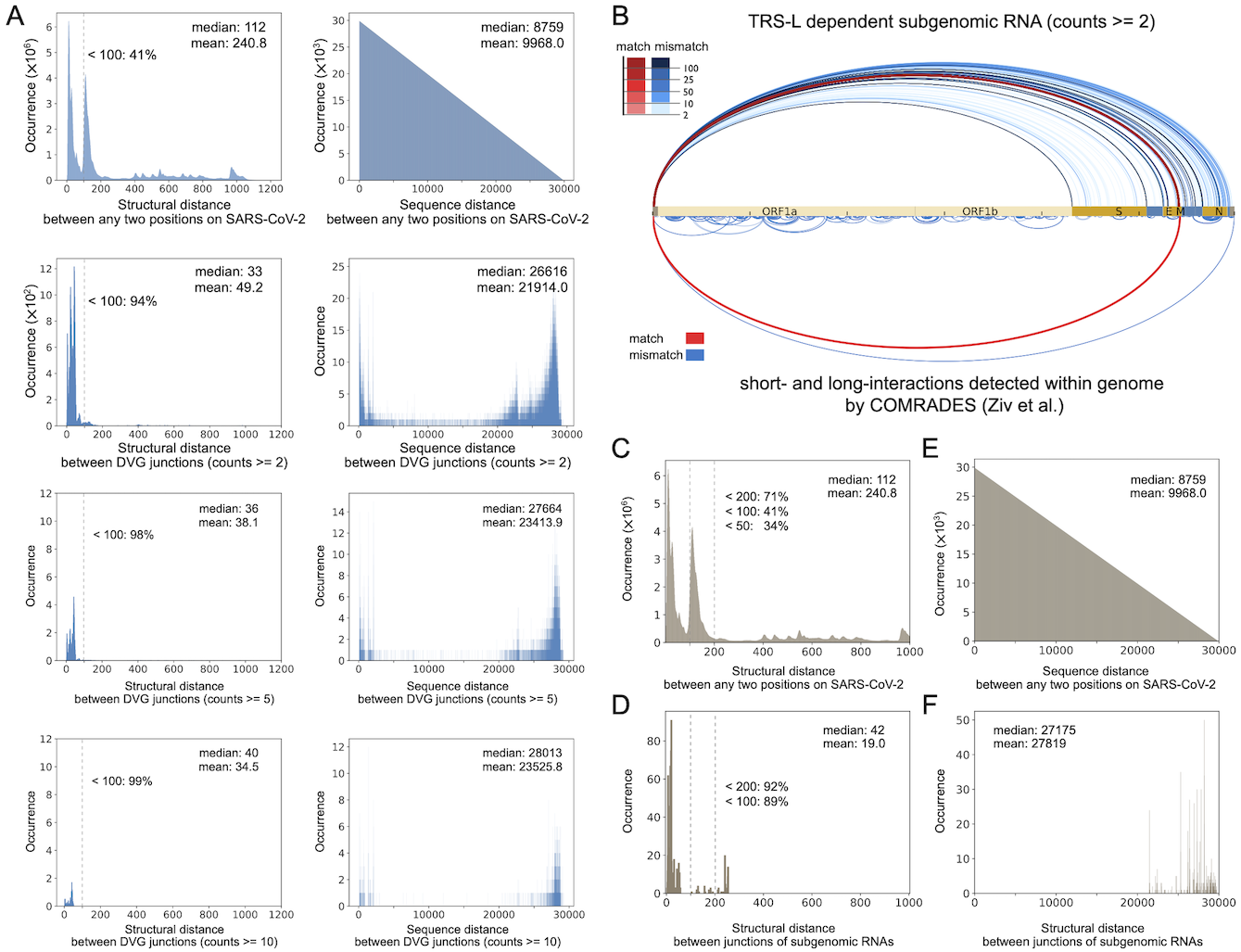

Supplement: FIG S4 [file mbio.00250-23-s0004.tif]

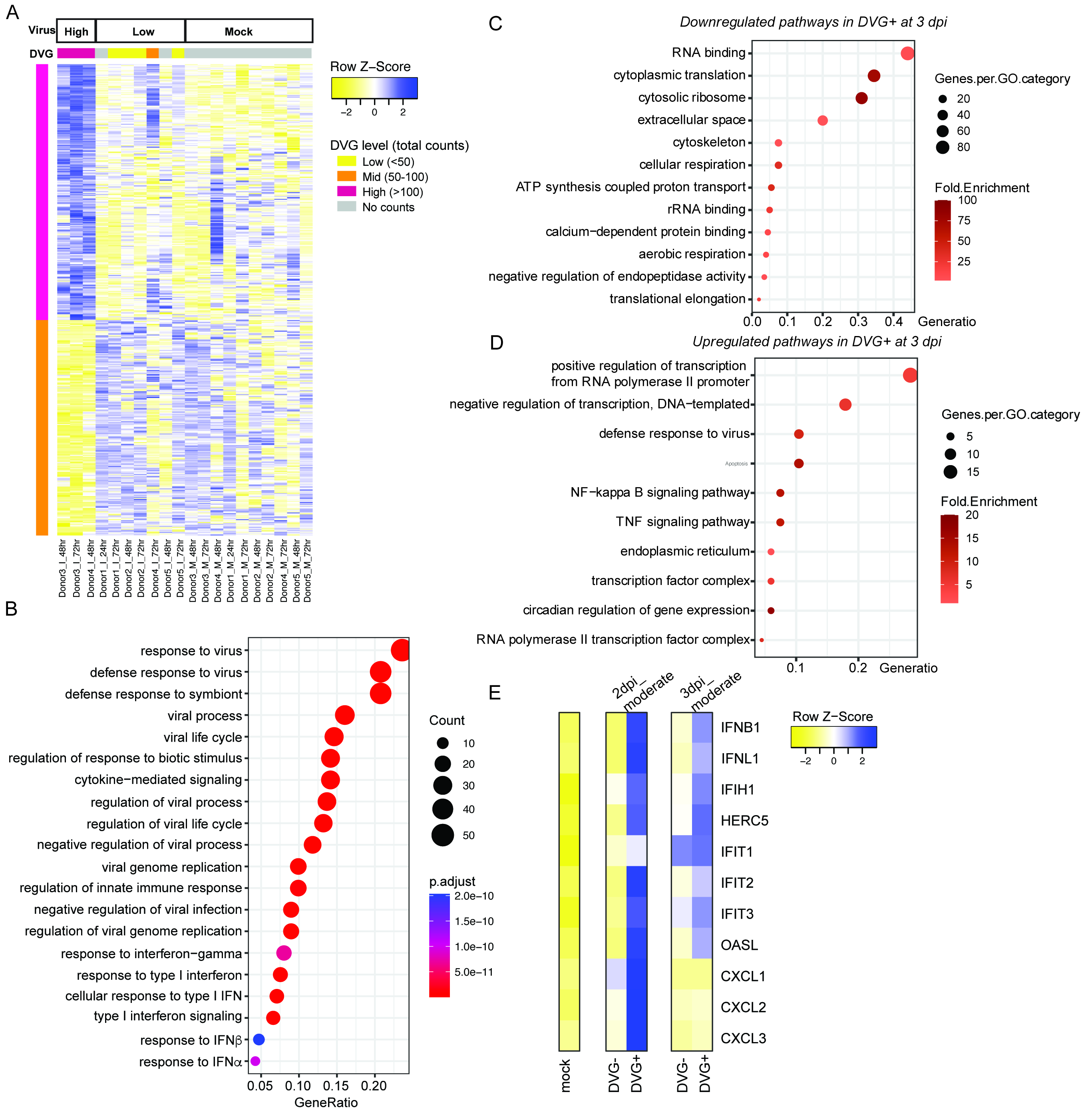

Supplement: FIG S5 [file mbio.00250-23-s0005.tif]

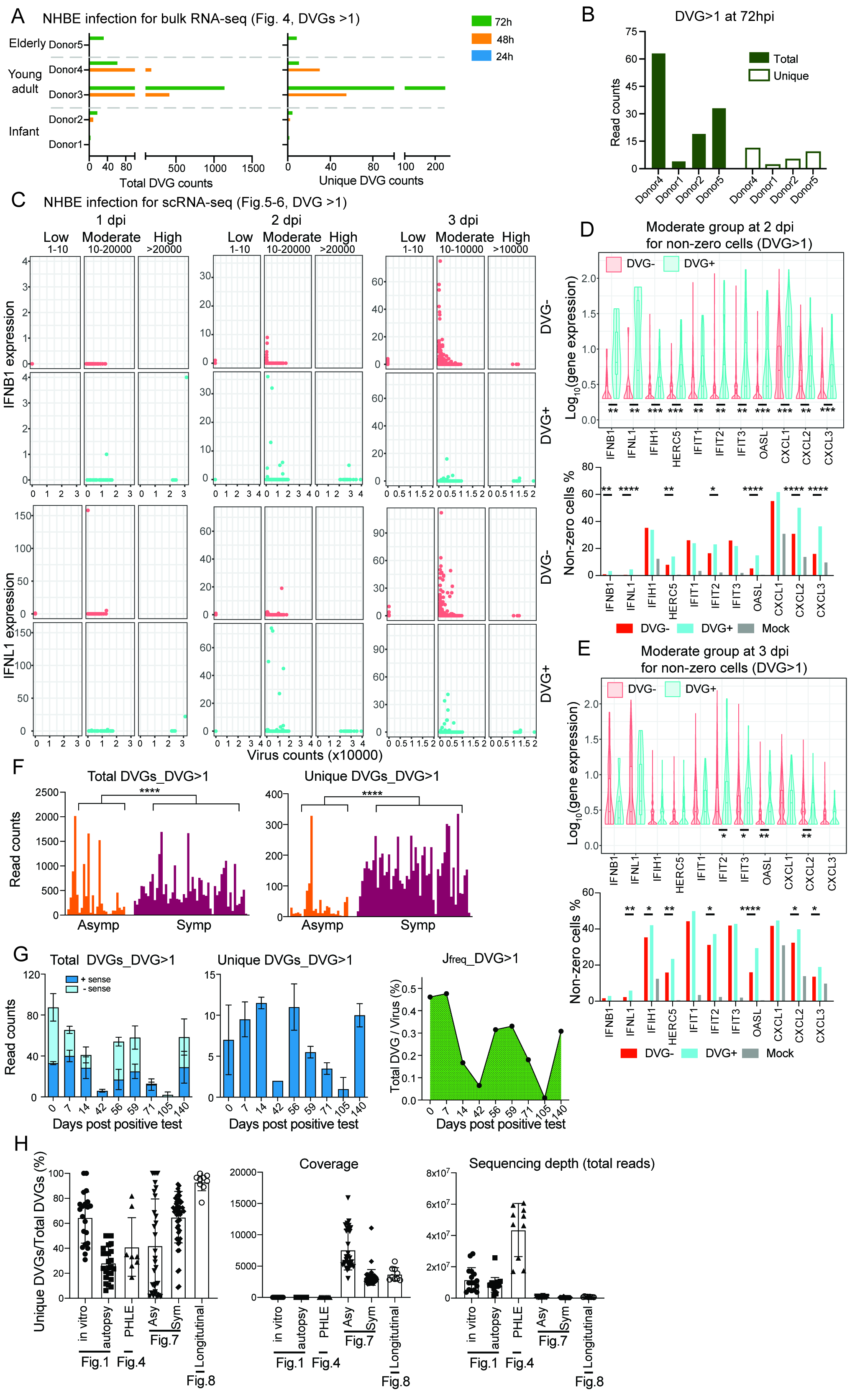

Supplement: FIG S6 [file mbio.00250-23-s0006.tif]
