# Supplementary material for: Generation and Functional Analysis of Defective Viral Genomes during SARS-CoV-2 Infection
Source: mBio. 2023 Apr 19;14(3):e00250-23. doi: 10.1128/mbio.00250-23 (PMC10294654; doi:10.1128/mbio.00250-23)
Supplement: TABLE S2 [file mbio.00250-23-s0008.docx]

**Table S2 Common DVGs identified from *in vitro* infections**

| **GSE147507** | | | | | |
| --- | --- | --- | --- | --- | --- |
| Break Point | | Rejoin Point | Counts | Strand | ID |
| 28691 | | 1620 | 109 | - | A549-ACE2_r1 |
| 28691 | | 1620 | 49 | - | A549-ACE2_r3 |
| 29173 | | 27800 | 8 | - | NHBE_r2 |
| 29173 | | 27800 | 104 | - | A549-ACE2_r1 |
| 29173 | | 27800 | 92 | - | A549-ACE2_r2 |
| 29173 | | 27800 | 63 | - | A549-ACE2_r3 |
| 29307 | | 731 | 129 | - | A549-ACE2_r1 |
| 29307 | | 731 | 115 | - | A549-ACE2_r2 |
| 29307 | | 731 | 96 | - | A549-ACE2_r3 |
| 29308 | | 733 | 52 | - | A549-ACE2_r1 |
| 29308 | | 733 | 57 | - | A549-ACE2_r2 |
| 29308 | | 733 | 46 | - | A549-ACE2_r3 |
| 29310 | | 747 | 76 | - | A549-ACE2_r1 |
| 29310 | | 747 | 109 | - | A549-ACE2_r2 |
| 29310 | | 747 | 48 | - | A549-ACE2_r3 |
| 29310 | | 755 | 55 | - | A549-ACE2_r1 |
| 29310 | | 755 | 56 | - | A549-ACE2_r2 |
| 29310 | | 755 | 54 | - | A549-ACE2_r3 |
| 29310 | | 827 | 89 | - | A549-ACE2_r1 |
| 29310 | | 827 | 99 | - | A549-ACE2_r2 |
| 29310 | | 827 | 49 | - | A549-ACE2_r3 |
| 29350 | | 824 | 66 | - | A549-ACE2_r1 |
| 29350 | | 824 | 110 | - | A549-ACE2_r2 |
| 29350 | | 824 | 92 | - | A549-ACE2_r3 |
| 29353 | | 734 | 13 | - | NHBE_r2 |
| 29353 | | 734 | 104 | - | A549-ACE2_r1 |
| 29353 | | 734 | 52 | - | A549-ACE2_r3 |
| 29353 | | 735 | 64 | - | A549-ACE2_r1 |
| 29353 | | 735 | 68 | - | A549-ACE2_r3 |
| 29477 | | 730 | 54 | - | A549-ACE2_r2 |
| 29477 | | 730 | 68 | - | A549-ACE2_r3 |
| **GSE148729** | | | | | |
| 27234 | | 27344 | 14 | + | calu3_totalRNA_AR2 |
| 27234 | | 27344 | 13 | + | calu3_totalRNA_BR2 |
| 27341 | | 27231 | 11 | - | calu3_polyA_A |
| 27341 | | 27231 | 9 | - | calu3_polyA_B |
| 27341 | | 27231 | 25 | - | calu3_totalRNA_AR1 |
| 27341 | | 27231 | 25 | - | calu3_totalRNA_BR1 |
| 27341 | | 27231 | 11 | - | caco2_polyA_A |
| 27341 | | 27231 | 24 | - | caco2_polyA_B |
| 27794 | | 29175 | 19 | + | calu3_totalRNA_AR2 |
| 27794 | | 29175 | 10 | + | calu3_totalRNA_BR2 |
| 27794 | | 29176 | 12 | + | calu3_totalRNA_BR2 |
| 27795 | | 29175 | 12 | + | calu3_totalRNA_AR2 |
| 27796 | | 29195 | 8 | + | calu3_totalRNA_BR2 |
| 27802 | | 29175 | 29 | + | calu3_totalRNA_AR2 |
| 27802 | | 29175 | 28 | + | calu3_totalRNA_BR2 |
| 27965 | | 27231 | 3 | - | calu3_polyA_A |
| 27965 | | 27231 | 9 | - | calu3_totalRNA_AR1 |
| 27965 | | 27231 | 9 | - | caco2_polyA_B |
| 28318 | | 29123 | 8 | + | calu3_totalRNA_AR2 |
| 28318 | | 29123 | 13 | + | calu3_totalRNA_BR2 |
| 28319 | | 29017 | 12 | + | calu3_totalRNA_AR2 |
| 28319 | | 29017 | 7 | + | calu3_totalRNA_BR2 |
| 28408 | | 29017 | 11 | + | calu3_totalRNA_AR2 |
| 28408 | | 29017 | 9 | + | calu3_totalRNA_BR2 |
| 28673 | | 28505 | 9 | - | calu3_totalRNA_BR1 |
| 28673 | | 28505 | 5 | - | caco2_polyA_A |
| 28729 | | 28464 | 13 | - | caco2_polyA_A |
| 28729 | | 28464 | 6 | - | caco2_polyA_B |
| 28731 | | 28465 | 13 | - | caco2_polyA_A |
| 28731 | | 28465 | 8 | - | caco2_polyA_B |
| 28731 | | 28495 | 8 | - | caco2_polyA_A |
| 28731 | | 28495 | 5 | - | caco2_polyA_B |
| 29084 | | 28318 | 12 | - | calu3_totalRNA_AR1 |
| 29084 | | 28318 | 11 | - | calu3_totalRNA_BR1 |
| 29084 | | 28318 | 6 | - | caco2_polyA_A |
| 29084 | | 28318 | 8 | - | caco2_polyA_B |
| 29164 | | 27800 | 4 | - | calu3_polyA_A |
| 29164 | | 27800 | 6 | - | caco2_polyA_A |
| 29173 | | 27792 | 8 | - | calu3_polyA_B |
| 29173 | | 27793 | 6 | - | calu3_polyA_B |
| 29173 | | 27793 | 6 | - | caco2_polyA_A |
| 29173 | | 27800 | 16 | - | calu3_polyA_A |
| 29173 | | 27800 | 15 | - | calu3_polyA_B |
| 29173 | | 27800 | 16 | - | calu3_totalRNA_AR1 |
| 29173 | | 27800 | 14 | - | calu3_totalRNA_BR1 |
| 29173 | | 27800 | 25 | - | caco2_polyA_A |
| 29173 | | 27800 | 12 | - | caco2_polyA_B |
| 29173 | | 27801 | 3 | - | calu3_polyA_A |
| 29173 | | 27801 | 6 | - | caco2_polyA_A |
| 29343 | | 6653 | 3 | - | calu3_polyA_A |
| 29343 | | 6655 | 5 | - | caco2_polyA_B |
| 29345 | | 6635 | 3 | - | calu3_polyA_A |
| 29353 | | 6603 | 3 | - | calu3_polyA_B |
| 29353 | | 6653 | 7 | - | calu3_totalRNA_AR1 |
| 29481 | | 6683 | 3 | - | calu3_polyA_B |
| 29481 | | 6683 | 9 | - | calu3_totalRNA_BR1 |
| 29493 | | 6653 | 3 | - | calu3_polyA_A |
| 29494 | | 6653 | 4 | - | calu3_polyA_A |
| 29494 | | 6653 | 3 | - | calu3_polyA_B |
| 29495 | | 6655 | 10 | - | calu3_totalRNA_BR1 |
| 29520 | | 6883 | 3 | - | calu3_polyA_B |
| 29520 | | 6883 | 11 | - | calu3_totalRNA_BR1 |
| 29805 | | 29686 | 8 | - | caco2_polyA_A |
| 29805 | | 29686 | 9 | - | caco2_polyA_B |
| **SRP258466** | | | | | |
| Break Point | Rejoin Point | | Counts | Strand | ID |
| 5981 | 6566 | | 8 | + | veroE6_L8 |
| 5981 | 6566 | | 3 | + | veroE6_s5p1 |
| 5981 | 6566 | | 9 | + | veroE6_s5p2 |
| 5981 | 6566 | | 5 | + | veroE6_s5p3 |
| 20272 | 20387 | | 8 | + | veroE6_L8 |
| 20272 | 20387 | | 5 | + | veroE6_s5p1 |
| 20272 | 20387 | | 6 | + | veroE6_s5p2 |
| 20272 | 20387 | | 9 | + | veroE6_s5p3 |
| 27386 | 29473 | | 13 | + | veroE6_L8 |
| 27386 | 29473 | | 2 | + | veroE6_s5p1 |
| 27386 | 29473 | | 17 | + | veroE6_s5p2 |
| 27386 | 29472/29473 | | 13 | + | veroE6_s5p3 |
| 27788 | 29196 | | 23 | + | veroE6_L8 |
| 27788 | 29196 | | 8 | + | veroE6_s5p1 |
| 27788 | 29196 | | 5 | + | veroE6_s5p2 |
| 27788 | 29196 | | 26 | + | veroE6_s5p3 |
| 27794 | 29175 | | 7 | + | veroE6_s5p1 |
| 27794 | 29175 | | 2 | + | veroE6_s5p2 |
| 27794 | 29175 | | 9 | + | veroE6_s5p3 |
| 27794 | 29175 | | 4 | + | veroE6_L8 |
| 27802 | 29175 | | 14 | + | veroE6_L8 |
| 27802 | 29175 | | 6 | + | veroE6_ s5p1 |
| 27802 | 29175 | | 5 | + | veroE6_ s5p2 |
| 27802 | 29175 | | 11 | + | veroE6_s5p3 |
| 28508 | 28676 | | 22 | + | veroE6_L8 |
| 28508 | 28676 | | 7 | + | veroE6_s5p1 |
| 28508 | 28676 | | 11 | + | veroE6_s5p2 |
| 28508 | 28676 | | 22 | + | veroE6_s5p3 |
| **PHLE cells in vitro infections (own infection)** | | | | | |
| Break Point | | Rejoin Point | Counts | Strand | ID |
| 1363 | | 29345 | 6 | + | D231_I_72hr_R1 |
| 1363 | | 29353 | 4 | + | D231_I_72hr_R1 |
| 1369 | | 29353 | 1 | + | D283_I_72hr_R1 |
| 1624 | | 29337 | 15 | + | D231_I_72hr_R1 |
| 1624 | | 29339 | 1 | + | D231_I_72hr_R1 |
| 27382 | | 29472 | 10 | + | D231_I_72hr_R1 |
| 27382 | | 29473 | 7 | + | D231_I_72hr_R1 |
| 27385 | | 29473 | 14 | + | D231_I_48hr_R1 |
| 27385 | | 29479 | 9 | + | D231_I_72hr_R1 |
| 27385 | | 29472 | 7 | + | D231_I_72hr_R1 |
| 27385 | | 29473 | 3 | + | D231_I_72hr_R1 |
| 27385 | | 29473 | 2 | + | D239_I_48hr_R1 |
| 27386 | | 29473 | 12 | + | D231_I_72hr_R1 |
| 27386 | | 29476 | 4 | + | D231_I_72hr_R1 |
| 27386 | | 29474 | 1 | + | D231_I_72hr_R1 |
| 27386 | | 29473 | 9 | + | D239_I_48hr_R1 |
| 27793 | | 29166 | 4 | + | D231_I_48hr_R1 |
| 27794 | | 29167 | 3 | + | D231_I_72hr_R1 |
| 27794 | | 29166 | 2 | + | D231_I_72hr_R1 |
| 27795 | | 29166 | 11 | + | D231_I_72hr_R1 |
| 27793 | | 29176 | 1 | + | D231_I_72hr_R1 |
| 27794 | | 29175 | 5 | + | D231_I_72hr_R1 |
| 27795 | | 29176 | 2 | + | D231_I_72hr_R1 |
| 27795 | | 29175 | 2 | + | D231_I_72hr_R1 |
| 27795 | | 29175 | 22 | + | D239_I_72hr_R1 |
| 27798 | | 29176 | 7 | + | D231_I_72hr_R1 |
| 27800 | | 29174 | 1 | + | D239_I_48hr_R1 |
| 27801 | | 29175 | 4 | + | D231_I_72hr_R1 |
| 27802 | | 29175 | 21 | + | D231_I_48hr_R1 |
| 27802 | | 29166 | 12 | + | D231_I_72hr_R1 |
| 27802 | | 29175 | 9 | + | D231_I_72hr_R1 |
| 27802 | | 29176 | 3 | + | D231_I_72hr_R1 |
| 27802 | | 29175 | 28 | + | D239_I_48hr_R1 |
| 27802 | | 29175 | 1 | + | D283_I_72hr_R1 |
| 27803 | | 29174 | 1 | + | D203_I_72hr_R1 |
| 27803 | | 29175 | 10 | + | D231_I_72hr_R1 |
| 27803 | | 29172 | 3 | + | D231_I_72hr_R1 |
| 29172 | | 27803 | 3 | - | D231_I_72hr_R1 |
| 29173 | | 27802 | 1 | - | D203_I_72hr_R1 |
| 29173 | | 27800 | 10 | - | D231_I_48hr_R1 |
| 29173 | | 27801 | 10 | - | D231_I_72hr_R1 |
| 29173 | | 27800 | 7 | - | D231_I_72hr_R1 |
| 29173 | | 27793 | 4 | - | D231_I_72hr_R1 |
| 29173 | | 27792 | 3 | - | D231_I_72hr_R1 |
| 29173 | | 27800 | 12 | - | D239_I_48hr_R1 |
| 29173 | | 27800 | 4 | - | D283_I_72hr_R1 |
| 29174 | | 27800 | 2 | - | D231_I_72hr_R1 |
| 29175 | | 27797 | 2 | - | D231_I_72hr_R1 |
| 29176 | | 27802 | 2 | - | D231_I_72hr_R1 |
| 29471 | | 27383 | 1 | - | D231_I_48hr_R1 |
| 29472 | | 27382 | 1 | - | D231_I_72hr_R1 |
| 29473 | | 27386 | 3 | - | D231_I_72hr_R1 |
| 29473 | | 27386 | 1 | - | D239_I_48hr_R1 |
| 29474 | | 27389 | 1 | - | D198_I_72hr_R1 |
| 29474 | | 27386 | 1 | - | D231_I_72hr_R1 |
| 29475 | | 27385 | 1 | - | D231_I_72hr_R1 |
| 29695 | | 29814 | 8 | + | D231_I_72hr_R1 |
| 29695 | | 29814 | 4 | + | D239_I_48hr_R1 |
| 29805 | | 29686 | 10 | - | D231_I_72hr_R1 |
| 29807 | | 29688 | 3 | - | D203_I_48hr_R1 |
| 29807 | | 29688 | 6 | - | D231_I_48hr_R1 |
| 29807 | | 29688 | 7 | - | D231_I_72hr_R1 |
| 29807 | | 29688 | 16 | - | D239_I_48hr_R1 |
| 29807 | | 29688 | 1 | - | D283_I_72hr_R1 |
| 29810 | | 29687 | 13 | - | D231_I_48hr_R1 |
| 29812 | | 29686 | 1 | - | D231_I_72hr_R1 |
| 29813 | | 29690 | 4 | - | D231_I_72hr_R1 |
| 29813 | | 29690 | 15 | - | D239_I_48hr_R1 |
| 29814 | | 29693 | 1 | - | D231_I_72hr_R1 |
| 29817 | | 29690 | 1 | - | D231_I_72hr_R1 |
| 29818 | | 29686 | 4 | - | D231_I_48hr_R1 |
