# Supplementary material for: Generation and Functional Analysis of Defective Viral Genomes during SARS-CoV-2 Infection
Source: mBio. 2023 Apr 19;14(3):e00250-23. doi: 10.1128/mbio.00250-23 (PMC10294654; doi:10.1128/mbio.00250-23)
Supplement: TABLE S3 [file mbio.00250-23-s0009.docx]

**Table S3 Summary of validation of DVGs identified in the immunocompromised patient**

| **Time** | **Unique DVGs_n>1 (%)** | **^a^** **Total DVGs** | **^b^ Verified DVGs with matched junctions** | **^c^ Verified DVGs with ambiguity (<10 nts)** | **^d^ Verified DVGs with ambiguity (≥10 nts)** | **^e^ Total verified DVGs** | **^f^Accuracy (%)** |
| --- | --- | --- | --- | --- | --- | --- | --- |
| D0_R1 | 3.79 | 352 | 328 | 16 | 4 | 344 | 97.73 |
| D0_R2 | 1.53 | 335 | 307 | 15 | 3 | 322 | 96.12 |
| D7_R1 | 2.41 | 521 | 474 | 38 | 6 | 512 | 98.27 |
| D7_R2 | 1.74 | 508 | 452 | 38 | 6 | 490 | 96.46 |
| D14_R1 | 1.41 | 885 | 817 | 57 | 4 | 874 | 98.76 |
| D14_R2 | 1.37 | 833 | 740 | 62 | 6 | 802 | 96.28 |
| D42_R1 | 0.87 | 221 | 197 | 19 | 3 | 216 | 97.74 |
| D42_R2 | 0.93 | 233 | 213 | 16 | 2 | 229 | 98.28 |
| D56_R1 | 0.74 | 1258 | 1154 | 92 | 6 | 1246 | 99.05 |
| D56_R2 | 1.11 | 1211 | 1080 | 109 | 2 | 1189 | 98.18 |
| D59_R1 | 0.89 | 610 | 564 | 36 | 3 | 600 | 98.36 |
| D59_R2 | 1.13 | 587 | 539 | 35 | 2 | 574 | 97.79 |
| D71_R1 | 2.01 | 211 | 196 | 12 | 2 | 208 | 98.58 |
| D71_R2 | 1.63 | 191 | 171 | 13 | 1 | 184 | 96.34 |
| D105_R1 | 0.18 | 1138 | 1054 | 69 | 2 | 1123 | 98.68 |
| D105_R2 | 0 | 1110 | 1000 | 100 | 1 | 1100 | 99.10 |
| D140_R1 | 1.22 | 783 | 722 | 52 | 3 | 774 | 98.85 |
| D140_R2 | 1.48 | 793 | 689 | 88 | 2 | 777 | 97.98 |

^a^ Total DVG counts identified by ViReMa post R filtering.

^b^ DVG reads with ViReMa-identified junctions exactly matching BLAST-identified split positions.

^c^ DVG reads with ViReMa-identified junctions different from BLAST-identified split positions by less than 10nts.

^d^ DVG reads with ViReMa-identified junctions different from BLAST-identified split positions by more than 10 nts.

^e, f^ We only consider DVGs with either exact matching junctions or off by less than 10 nts as accurately identified DVGs by ViReMa.
